# Global, regional, and national disease burden and economic costs of cervical cancer (1991–2021): a multidimensional data synthesis analysis

**DOI:** 10.3389/fpubh.2025.1633975

**Published:** 2025-09-11

**Authors:** Yidi Ma, Xiaozhen Lai, Hai Fang

**Affiliations:** ^1^Department of Health Policy and Management, School of Public Health, Peking University, Beijing, China; ^2^China Center for Health Development Studies, Peking University, Beijing, China; ^3^Peking University Health Science Center-Chinese Center for Disease Control and Prevention Joint Center for Vaccine Economics, Peking University, Beijing, China

**Keywords:** cervical cancer, cost of illness, disability-adjusted life-years, epidemiology, global

## Abstract

**Objectives:**

Cervical cancer remains a significant global health concern, particularly in less developed regions. This study aims to assess the global, regional, and national burden of cervical cancer from 1991 to 2021.

**Methods:**

This study synthesizes data from the Global Burden of Disease (GBD) Study 2021, WHO health expenditure databases, and published cost estimates to comprehensively assess the epidemiological and economic burden of cervical cancer from 1991 to 2021. We analyzed age-standardized rates (ASRs) of prevalence (ASPR), incidence (ASIR), mortality (ASMR), and disability-adjusted life years (ASDR) using GBD 2021 data. Temporal trends were quantified via Joinpoint regression-derived average annual percentage changes (AAPCs). Direct medical costs were estimated by integrating GBD incidence data, WHO per-capita health expenditure growth rates, and published treatment costs (2015), reported in both undiscounted and 3% discounted 2021 USD.

**Results:**

In 2021, globally, there were 3,385,000 prevalent cases, 667,000 incident cases, 297,000 deaths, and 7,440,000 DALYs attributed to cervical cancer. Sub-Saharan Africa bore the highest burden, while 12 countries in North Africa and Middle East reported incidence rates below 4 per 100,000. From 1991 to 2021, AAPCs in ASPR, ASIR, ASMR, and ASDR were 0.08%, −0.52%, −1.22%, and −1.21%, respectively. Despite the 2018 elimination initiative, overall incidence and mortality trends showed minimal change. Age-specific incidence notably decreased in individuals over 70, with slower mortality declines in higher age groups. AAPCs positively correlated with 1991 baseline rates and negatively with 2021 socio-demographic index (SDI). Over the same period, the global cumulative direct medical cost of cervical cancer was estimated at USD 9.26 billion (95% UI: 7.95–10.70) without discounting, and USD 7.21 billion (95% UI: 6.14–8.40) when discounted to 2021.

**Conclusion:**

The global cervical cancer prevalence continues to rise, with no country achieving the elimination threshold. High incidence is concentrating in younger ages, while high mortality is shifting to older ages. Sub-Saharan Africa requires targeted interventions to address its disproportionate burden. The substantial economic burden reinforces the urgency for early prevention, equitable treatment access, and sustained health investment.

## Introduction

Cervical cancer is a significant global health issue, leading to high morbidity and mortality among women (1). In 2022, global cervical cancer cases reached 661,000 with 348,000 deaths (2, 3), with an estimated 44.4 million cases during 2020–2069 without further intervention (4). Fortunately, cervical cancer is preventable and curable with actively HPV vaccination, early screening, and effective management of cervical lesions. In 2018, the WHO called for action to eliminate cervical cancer (5), presenting a global strategy in 2020 to reach the 90-70-90 targets: vaccinating 90% of girls against HPV by age 15, screening 70% of women with a high-performance test by ages 35 and 45, and treating 90% of women with cervical disease. Achieving these targets could reduce annual new cases to ≤ 4 per 100,000 women in most countries (6), meeting the 2030 UN Sustainable Development Goal (7).

In 2020, cervical cancer incidence and mortality varied widely across countries, ranging from 2.2 to 84.6 and 1.0 to 55.7 per 100,000 women-years, respectively (8). This discrepancy is partially explained by regional development levels; for example, incidence, mortality, and disability-adjusted life years (DALYs) decreased with increasing Socio-Demographic Index (SDI) levels (9). Globally, the estimated annual percentage change (EAPC) of age-standardized incidence rate (ASIR) and age-standardized mortality rate (ASMR) reduced by 0.38% and 0.93% per year from 1990 to 2019 (10). The above metrics were negatively correlated to Human Development Index (HDI) (11), due to the high-income countries usually possess the resources and ability for well-established programs of vaccination and screening (4). Moreover, effective treatment strategies could improve survival and mortality, influencing prevalence, an indicator of cervical cancer's burden on healthcare systems and society (7). However, previous studies have provided limited information on prevalence (9, 10, 12–15) and transition patterns in different age groups or countries (16).

In addition to epidemiological burden, cervical cancer imposes a substantial economic burden on health systems and families, particularly in low-resource settings. However, global evidence on cervical cancer–related direct medical costs remains limited and fragmented. Most existing cost-effectiveness or budget impact studies focus on single-country analyses or specific vaccine interventions (17), lacking harmonized methodologies to allow for global cross-country comparisons. Systematic estimates of direct treatment costs using consistent frameworks are needed to inform equitable policy decisions.

We hypothesize that cervical cancer burden varies significantly by geography, age, and socio-demographic development, with temporal trends reflecting differential access to prevention and care. Leveraging the Global Burden of Disease (GBD) 2021 framework (18, 19), this study provides the most updated assessment of global cervical cancer epidemiology, reporting 2021 prevalence, incidence, mortality, and DALYs, and analyzing trends from 1991 to 2021 stratified by age and SDI. Additionally, we estimate the direct medical costs across 194 countries (1990–2021) by integrating GBD incidence data, WHO health expenditure trends, and published treatment costs—addressing a critical gap in understanding the disease's financial toll. These findings aim to inform targeted resource allocation and policy prioritization.

## Methods

### Data source

This study integrates multiple complementary data sources to comprehensively assess cervical cancer burden: (1) epidemiological estimates from the Global Burden of Disease study, (2) health expenditure records from WHO databases, and (3) published treatment cost benchmarks. The GBD 2021 study, which provides comparative estimates of disease burden for 371 diseases and injuries across 204 countries and territories from 1990 to 2021, For all final estimates, 95% uncertainty intervals (UIs) were generated as the 2.5th and 97.5th percentile values from 500 posterior draws. Uncertainty was systematically propagated through each computational step of the GBD modeling hierarchy, including data inputs, model selection, and cause-specific mortality corrections. Detailed methodological approaches for GBD 2021 have been published previously (18–20). Countries were categorized into seven super-regions and 21 regions based on the GBD classification. This standardized grouping, developed by the GBD consortium, aggregates countries by geographic continuity, shared disease patterns, and socio-demographic development levels—factors. The GBD study was performed in accordance with the Guidelines for Accurate and Transparent Health Estimates Reporting. The SDI is a composite measure that summarizes a country or region's overall development level based on income per capita, average educational attainment, and total fertility rate (9). It ranges from 0 to 1, with higher values indicating higher development levels. The UHC Service Coverage Index assesses the extent to which a population has access to and utilizes essential health services, such as immunizations, maternal care, and treatment for common diseases (21). Higher scores indicate a larger proportion of the population benefiting from these interventions.

The analysis process and repeatable statistical codes for estimation can be retrieved from the GBD supporting website (https://ghdx.healthdata.org/gbd-2021/code). Cervical cancer was defined as malignant neoplasms of the cervix, identified using the International Classification of Diseases 10th edition diagnostic criteria for cervical cancer (C53).

To estimate the direct economic burden, we used country-level per capita health expenditure data (2000–2022) from the Global Health Expenditure Database (https://apps.who.int/nha/database/country_profile) and combined it with published estimates of 2015 cervical cancer treatment costs. A total of 194 countries were included. Unit costs were projected backward from 2015 using country-specific annual growth rates in health spending.

### Statistical analysis

Age-standardized rates of prevalence (ASPR), ASIR, ASMR and age-standardized rates of DALY (ASDR) were calculated to assess variations in cervical cancer burden across different time periods and geographical locations, ensuring adjustments for differences in age distribution among populations. Uncertainty intervals (UIs) were derived as the 2.5th and 97.5th percentiles of 1,000 posterior distribution draws.

To analyze the temporal trends in ASRs of cervical cancer burden, we calculated the average annual percentage change (AAPC) using a regression model, expressed as ln(*ASR*) = α+β × *calendar year*+ε. The AAPC was estimated along with its 95% confidence interval (CI) using the formula 100 × exp(β)−1. Joinpoint regression analysis (Joinpoint Regression Program, version 5.0.2, National Cancer Institute, USA) was applied to identify significant trend changes in ASRs from 1991 to 2021 globally and within SDI-based regions. This analysis utilizes piecewise linear regression to ascertain adaptive trends through one or more lines segments.

Spearman's rank correlation was employed to quantify the association between AAPCs in cervical cancer burden, the baseline burden in 1991, and the SDI in 2021 at the national level. A LOESS curve with a span parameter of 0.75 was used to smooth the relationship between the SDI and the AAPC. Local weighted scatter plot smoothing regression was used to display more detailed information regarding the estimated annual percentage change in ASR and possible factors. To evaluate the WHO's 2018 cervical cancer elimination initiative, we analyzed ASIR and ASMR trends (2014–2021) across 21 GBD regions using LOESS regression, stratified by SDI. Analyses were performed using R version 4.2.3 (R Foundation for Statistical Computing, Vienna, Austria), with statistical significance set at *P* < 0.05 (two-sided).

The direct economic burden was estimated by multiplying annual GBD incidence estimates with country-specific unit treatment costs projected over time. Treatment cost projections were derived by adjusting 2015 cost baselines using compound annual growth rates in per capita health expenditure. Total annual burden was expressed in 2021 USD and reported under both undiscounted and 3% discounted conditions. We calculated point estimates and 95% uncertainty intervals for each country and each year. Results were aggregated by country and globally.

## Results

### Global burden of cervical cancer in 2021

**Figure 1:**
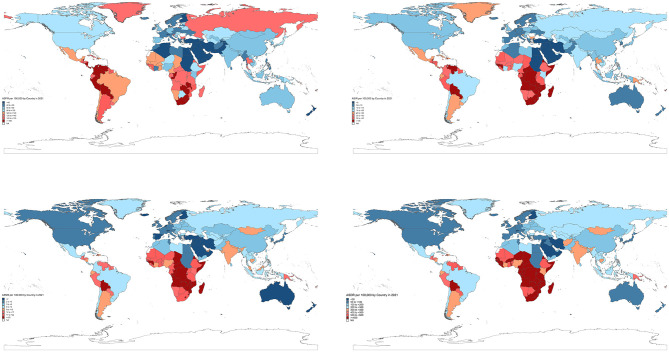
The global disease burden of cervical cancer in 204 countries and territories. **(a)** Age-standardized rates (ASRs) of prevalence, incidence, mortality, and DALYs in 2021. **(b)** Average annual percentage change (AAPC) in ASRs from 1991 to 2021.

**Figure 2:**
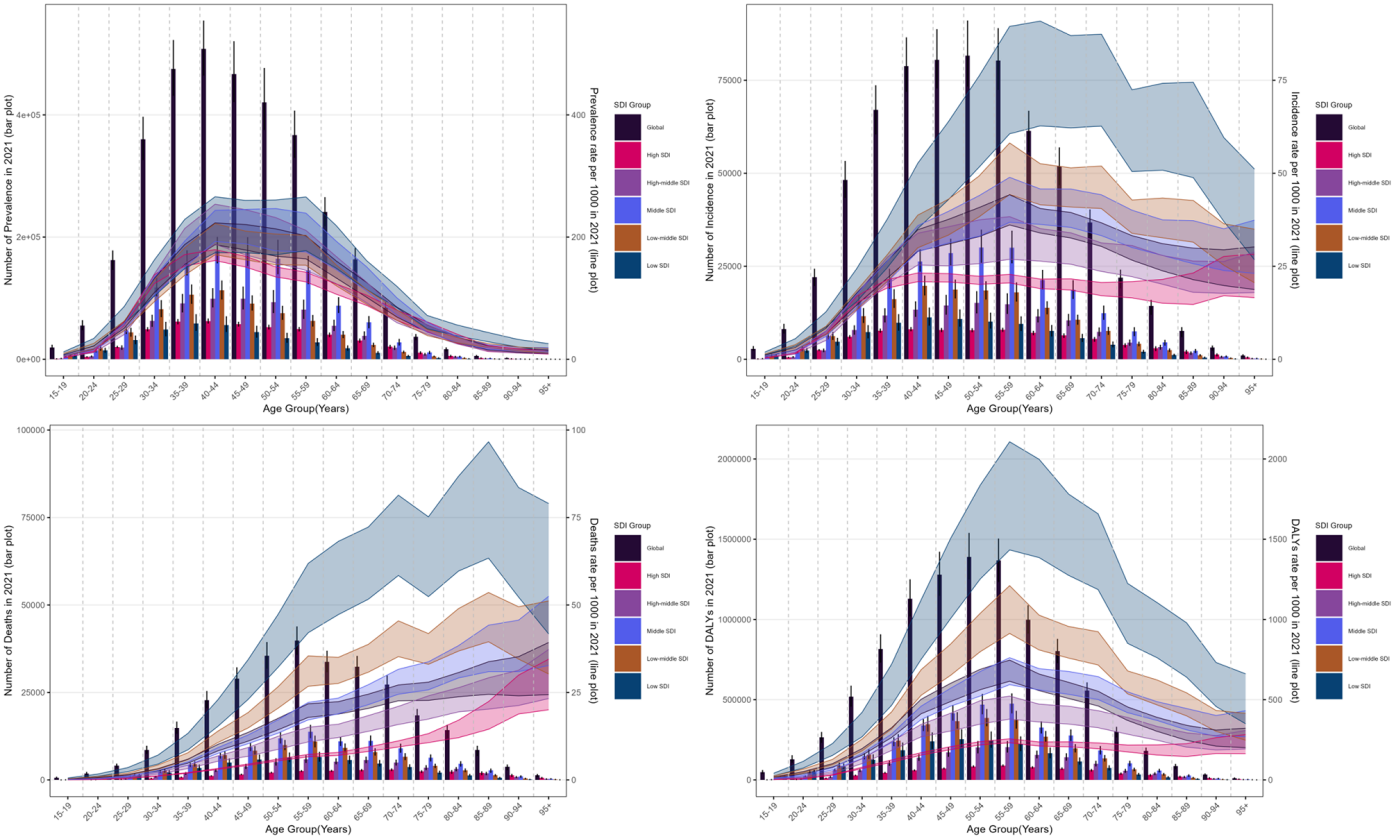
The age distribution of cases and rate of cervical cancer burden by SDI region in 2021. **(a)** Prevalence. **(b)** Incidence. **(c)** Mortality. **(d)** Disability-adjusted life years (DALYs).

Globally, cervical cancer caused 667,000 new cases (95%UI: 613,000 to 726,000) and 297,000 deaths (95%UI: 272,000 to 322,000), resulting in 7,440,000 (95%UI: 6,876,000 to 8,070,000) DALYs. The burden showed an inverse relationship with SDI quintile, with middle SDI resions exhibiting high prevalence rates (ASPR: 83.80; 95% UI: 74.69–93.87 per 100,000). Geographic disparities were pronounced, with Southern Sub-Saharan Africa showing the highest ASIR (42.40; 37.16–47.85) and North Africa/Middle East the lowest (4.72; 4.04–5.50) (Figure 1a, Table 1, Supplementary Table S1). Twelve countries achieved incidence rates below WHO's 4/100,000 threshold, ranging from Palestine (1.09; 0.85–1.36) to Saudi Arabia (3.86; 2.74–5.45). Kiribati demonstrated the highest rates for all indicators (ASIR: 70.03, 95% UI: 50.27–93.83; ASMR: 45.10, 32.22–58.73 per 100,000). In contrast, Palestine showed the lowest ASPR (6.28, 4.83–7.98) while Saudi Arabia and San Marino shared the lowest ASMR (0.89, 0.66–1.17 and 0.89, 0.55–1.39 respectively). Kuwait exhibited the lowest ASDR (26.60, 22.44–31.40). Analysis revealed consistent age-distribution patterns across metrics (Figure 2). Prevalence peaked at 40–59 years, while incidence showed a characteristic rise from age 30, peaking at 55–59 years. Mortality demonstrated a steady age-dependent increase across all regions. Notably, low-SDI regions exhibited delayed incidence peaks (60–64 years) and persistently higher mortality compared to high-SDI regions. DALY rates reached maximum values at 55–59 years before declining.

**Table 1:** Age-standardized rates of cervical cancer per 100,000 population in 2021.

	**ASPR per 100 000 people (95% UI)**	**ASIR per 100 000 people (95% UI)**	**ASMR per 100 000 people (95% UI)**	**ASDR per 100 000 people (95% UI)**
**Characteristic**	79.30 (72.81, 86.58)	15.32 (14.08, 16.68)	6.62 (6.07, 7.18)	226.28 (206.51, 246.86)
**SDI region**				
High SDI	68.67 (66.45, 70.84)	10.30 (9.91, 10.66)	2.62 (2.44, 2.74)	86.41 (82.45, 90.30)
High-middle SDI	79.72 (68.44, 91.34)	13.27 (11.44, 15.16)	4.59 (4.02, 5.20)	152.90 (133.88, 174.32)
Middle SDI	83.80 (74.69, 93.87)	15.94 (14.30, 17.75)	6.72 (6.05, 7.43)	218.95 (197.60, 242.31)
Low-middle SDI	74.93 (66.83, 83.29)	17.79 (15.94, 19.70)	9.71 (8.67, 10.70)	321.36 (287.11, 356.77)
Low SDI	90.16 (75.64, 107.64)	25.47 (21.57, 30.12)	16.36 (13.94, 19.38)	535.11 (454.02, 638.34)
**GBD region**				
High-income Asia Pacific	79.24 (73.58, 85.98)	11.03 (10.25, 11.95)	2.53 (2.28, 2.73)	87.68 (81.63, 94.36)
High-income North America	86.28 (82.71, 89.75)	12.69 (12.19, 13.22)	2.64 (2.50, 2.75)	92.68 (88.88, 96.91)
Western Europe	59.03 (56.44, 61.83)	8.71 (8.25, 9.11)	2.29 (2.11, 2.43)	72.71 (68.94, 76.61)
Australasia	60.80 (54.12, 67.62)	8.51 (7.66, 9.35)	1.85 (1.66, 2.02)	61.68 (56.34, 67.50)
Southern Latin America	132.90 (121.50, 145.38)	22.80 (21.07, 24.73)	8.56 (7.89, 9.28)	296.76 (276.24, 321.05)
Andean Latin America	170.17 (129.85, 216.09)	29.79 (22.83, 37.66)	14.02 (10.85, 17.51)	431.74 (331.78, 543.15)
Tropical Latin America	109.51 (104.02, 115.29)	20.27 (19.16, 21.27)	8.38 (7.82, 8.86)	285.57 (269.19, 299.68)
Central Latin America	154.03 (131.84, 177.48)	28.89 (24.76, 33.10)	9.52 (8.21, 10.86)	315.97 (272.32, 363.77)
Caribbean	141.59 (118.09, 165.87)	27.58 (23.09, 32.63)	12.31 (10.33, 14.68)	433.17 (356.80, 526.53)
Eastern Europe	104.48 (93.46, 115.16)	16.49 (14.80, 18.10)	5.50 (4.96, 6.07)	200.77 (180.38, 221.47)
Central Europe	90.48 (80.59, 100.41)	15.93 (14.45, 17.53)	6.02 (5.53, 6.53)	191.83 (175.35, 209.14)
Central Asia	70.78 (61.74, 79.91)	14.17 (12.38, 15.92)	6.33 (5.62, 7.13)	213.84 (187.54, 241.95)
North Africa and Middle East	19.29 (16.43, 22.81)	4.72 (4.04, 5.50)	2.55 (2.17, 2.93)	80.04 (67.65, 93.68)
South Asia	63.62 (54.88, 73.03)	15.54 (13.47, 17.71)	8.72 (7.57, 9.88)	285.98 (247.23, 325.23)
Southeast Asia	75.11 (63.34, 87.74)	15.17 (12.91, 17.65)	7.45 (6.43, 8.59)	241.92 (207.75, 281.44)
East Asia	79.16 (57.42, 102.70)	13.40 (9.86, 17.38)	4.68 (3.55, 5.98)	151.15 (111.80, 195.41)
Oceania	109.68 (85.14, 162.49)	27.31 (21.47, 39.68)	15.27 (12.10, 22.32)	499.47 (390.49, 739.25)
Western Sub-Saharan Africa	83.68 (63.82, 102.81)	24.11 (18.93, 29.10)	15.57 (12.55, 18.53)	490.75 (383.61, 591.44)
Eastern Sub-Saharan Africa	117.66 (94.45, 152.15)	33.45 (27.17, 42.12)	21.68 (17.79, 26.85)	709.49 (572.26, 890.60)
Central Sub-Saharan Africa	133.67 (90.05, 182.02)	38.00 (26.28, 50.98)	25.10 (17.45, 33.97)	813.59 (562.84, 1,104.29)
Southern Sub-Saharan Africa	172.03 (147.39, 197.69)	42.40 (37.16, 47.85)	23.90 (21.02, 26.67)	788.82 (685.47, 885.31)

UI, Uncertainty intervals; ASPR, Age-standardized prevalence rate; ASIR, Age-standardized incidence rate; ASMR, Age-standardized mortality rate; ASDR, Age-standardized DALY rate.

### The temporal trend of cervical cancer burden by region and age from 1991 to 2021

The Joinpoint regression analysis revealed distinct temporal patterns in cervical cancer burden across different metrics (Table 2). Globally, we observed a modest annual increase in ASPR (AAPC: 0.08%, 95% CI: 0.06–0.09), contrasting with significant annual declines in ASIR (−0.52%, −0.54 to −0.50), ASMR (−1.22%, −1.23 to −1.20), and ASDR (−1.21%, −1.23 to −1.20). Regional variations were notable (Figure 1b, Supplementary Table S2), with high-middle SDI region exhibited increasing ASPR (AAPC: 0.86%, 0.83–0.90) alongside stable ASIR (0.03%, −0.01 to 0.07). Southern Sub-Saharan Africa experienced concerning upward trends across all metrics (ASIR: 1.10%, 0.93–1.28), while Australasia showed the most substantial improvements (ASMR: −3.52%, −3.76 to −3.26).

**Table 2:** Average annual percentage change in age-standardized rates, 1991–2021.

	**AAPC in ASRs from 1991 to 2021, % (95% CI)**			
	**Prevalence**	**Incidence**	**Mortality**	**DALYs**
**Characteristic**	0.08 (0.06, 0.09)	−0.52 (−0.54, −0.50)	−1.22 (−1.24, −1.20)	−1.21 (−1.23, −1.20)
**SDI region**				
High SDI	−1.17 (−1.20, −1.13)	−1.48 (−1.50, −1.46)	−2.07 (−2.11, −2.05)	−2.04 (−2.07, −2.02)
High-middle SDI	0.86 (0.83, 0.90)	0.03 (−0.01, 0.07)	−1.22 (−1.28, −1.16)	−1.15 (−1.20, −1.10)
Middle SDI	0.58 (0.55, 0.60)	−0.38 (−0.40, −0.37)	−1.42 (−1.44, −1.40)	−1.42 (−1.44, −1.40)
Low-middle SDI	−0.11 (−0.14, −0.08)	−0.76 (−0.78, −0.73)	−1.32 (−1.34, −1.29)	−1.46 (−1.48, −1.43)
Low SDI	−0.47 (−0.49, −0.44)	−0.99 (−1.01, −0.97)	−1.32 (−1.34, −1.31)	−1.47 (−1.49, −1.45)
**GBD region**				
High-income Asia Pacific	0.46 (0.36, 0.62)	−0.15 (−0.23, −0.07)	−1.65 (−1.70, −1.58)	−1.22 (−1.30, −1.14)
High-income North America	−1.32 (−1.38, −1.25)	−1.41 (−1.46, −1.35)	−1.14 (−1.20, −1.09)	−1.15 (−1.20, −1.10)
Western Europe	−1.21 (−1.31, −1.12)	−1.57 (−1.66, −1.49)	−2.30 (−2.38, −2.22)	−2.25 (−2.34, −2.18)
Australasia	−1.81 (−2.01, −1.63)	−2.27 (−2.48, −2.06)	−3.52 (−3.76, −3.26)	−3.42 (−3.63, −3.23)
Southern Latin America	0.29 (0.17, 0.39)	−0.28 (−0.39, −0.18)	−1.20 (−1.33, −1.10)	−1.16 (−1.29, −1.05)
Andean Latin America	0.64 (0.40, 0.92)	−0.38 (−0.54, −0.21)	−1.31 (−1.48, −1.13)	−1.44 (−1.62, −1.25)
Tropical Latin America	0.43 (0.37, 0.50)	−0.49 (−0.55, −0.43)	−1.52 (−1.56, −1.47)	−1.33 (−1.38, −1.27)
Central Latin America	−0.18 (−0.23, −0.13)	−1.15 (−1.20, −1.09)	−2.44 (−2.50, −2.38)	−2.19 (−2.24, −2.14)
Caribbean	0.11 (0.03, 0.21)	−0.36 (−0.43, −0.29)	−0.85 (−0.89, −0.79)	−0.76 (−0.80, −0.70)
Eastern Europe	1.17 (1.04, 1.33)	0.25 (0.13, 0.39)	−1.09 (−1.32, −0.93)	−0.58 (−0.68, −0.45)
Central Europe	−0.69 (−0.79, −0.61)	−1.09 (−1.16, −1.03)	−1.80 (−1.85, −1.75)	−2.01 (−2.07, −1.95)
Central Asia	−0.10 (−0.19, 0.01)	−0.48 (−0.58, −0.36)	−0.93 (−1.01, −0.84)	−0.93 (−1.01, −0.84)
North Africa and Middle East	−0.15 (−0.17, −0.11)	−0.76 (−0.78, −0.73)	−1.41 (−1.44, −1.39)	−1.57 (−1.59, −1.54)
South Asia	−0.65 (−0.70, −0.60)	−1.37 (−1.41, −1.33)	−1.92 (−1.98, −1.87)	−2.11 (−2.16, −2.07)
Southeast Asia	0.03 (−0.01, 0.07)	−0.59 (−0.63, −0.56)	−1.16 (−1.19, −1.13)	−1.30 (−1.33, −1.27)
East Asia	1.81 (1.75, 1.86)	0.41 (0.37, 0.45)	−1.27 (−1.32, −1.22)	−1.30 (−1.35, −1.25)
Oceania	−0.61 (−0.68, −0.54)	−0.78 (−0.82, −0.74)	−0.85 (−0.88, −0.82)	−0.89 (−0.92, −0.85)
Western Sub-Saharan Africa	0.01 (−0.01, 0.03)	−0.30 (−0.32, −0.29)	−0.55 (−0.57, −0.53)	−0.72 (−0.74, −0.70)
Eastern Sub-Saharan Africa	−0.49 (−0.51, −0.46)	−1.06 (−1.08, −1.03)	−1.40 (−1.42, −1.38)	−1.53 (−1.54, −1.51)
Central Sub-Saharan Africa	0.37 (0.31, 0.43)	−0.17 (−0.20, −0.14)	−0.47 (−0.51, −0.43)	−0.55 (−0.59, −0.50)
Southern Sub-Saharan Africa	1.03 (0.82, 1.25)	1.10 (0.93, 1.28)	1.01 (0.79, 1.21)	0.95 (0.76, 1.17)

AAPC, Average annual percentage change; ASPR, Age-standardized prevalence rate; ASIR, Age-standardized incidence rate; ASMR, Age-standardized mortality rate; ASDR, Age-standardized DALY rate.

**Figure 3:**
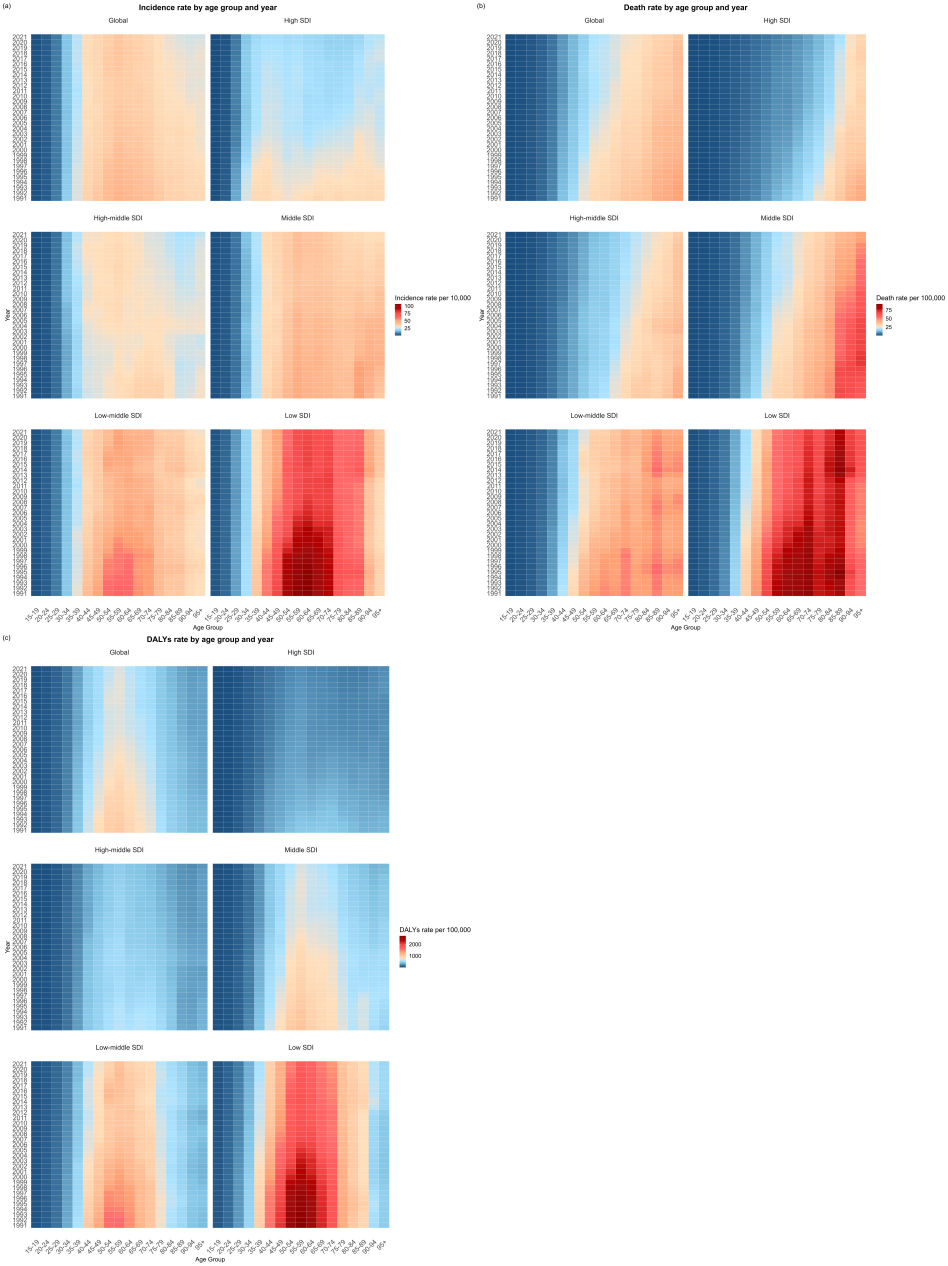
The change in the burden of cervical cancer by different age groups and sociodemographic index (SDI) regions from 1991 to 2021. **(a)** Change in age-specific incidence rate. **(b)** Change in age-specific deaths rate. **(c)** Change in age-specific DALYs rate.

Age-specific analyses revealed distinct patterns (Figure 3, Supplementary Tables S3–S5). Across SDI regions, incidence trends showed marked variations: the high SDI region demonstrated declines in all age groups with emerging dual peaks (40–44 and ≥85 years), while the high-middle SDI region exhibited a shift in peak incidence from ≥70 to 55–59 years. A transient increase occurred in 2003, most notably in the ≥80 age group, before resuming decline from 2006 onward. The most substantial incidence reductions were observed in ≥80 year-olds (middle SDI region) and 50–74 year-olds (low-middle and low SDI regions). Mortality patterns showed consistent declines across all age groups, with peak mortality shifting to older ages—reaching ≥95 years in high, high-middle and middle SDI regions, while remaining at 85–89 years in lower SDI regions. DALY rates decreased most significantly in 45–70 year-olds globally, with persistently low rates in high SDI regions vs. sustained high burden in women >40 years from low SDI regions.

### The related factors of global burden and temporal trends for cervical cancer

National-level analyses revealed significant correlations between baseline burden and subsequent trends (Supplementary Figure S1). Baseline measures in 1991 showed positive associations with AAPCs for incidence (ρ = 0.15, *P* = 0.033), mortality (ρ = 0.37, *P* < 0.001), and DALYs (ρ = 0.35, *P* < 0.001). The most pronounced ASMR reductions (−1.84%) occurred when baseline ASMR was 4.62/100,000. Development indicators strongly predicted improvement rates. Both SDI (ρ = −0.43 to −0.55, all *P* < 0.001) and UHC Service Coverage Index (ρ = −0.36 to −0.51, all *P* < 0.001) showed negative correlations with AAPCs (Supplementary Figure S2). Notably, when SDI exceeded 0.60, downward trends accelerated substantially. Regional analyses identified exceptions to global patterns (Supplementary Figure S3). While most regions showed declining ASIR with rising SDI, Tropical Latin America and parts of Sub-Saharan Africa demonstrated stagnant or increasing rates post-2018. Southern Sub-Saharan Africa showed particularly strong mortality reductions despite lower baseline development indicators.

### Global direct economic burden of cervical cancer, 1990–2021

**Figure 4:**
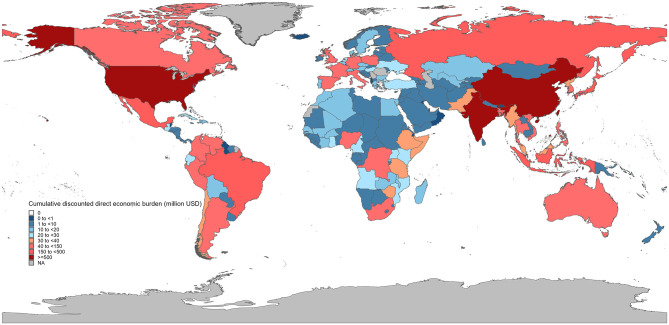
The cumulative discounted direct economic burden of cervical cancer across countries, 1990–2021.

The economic analysis revealed substantial global costs associated with cervical cancer over the 31-year study period. The cumulative undiscounted direct medical costs reached USD 9.26 billion (95% UI: 7.95–10.70 billion), while the discounted total (3% annual rate) was USD 7.21 billion (95% UI: 6.14–8.40 billion). Country-level analyses demonstrated marked variations in cervical cancer-related health expenditures (Figure 4, Supplementary Table S6). In 2021, China bore the heaviest economic burden (USD 256.14 million; 185.10–332.94 million), followed by India (USD 65.19 million; 95% UI: 56.31–75.89 million) and the United States (USD 65.19 million; 95% UI: 62.21–67.90 million). Conversely, several small island nations including Cook Islands, Nauru and Tuvalu maintained expenditures below USD 10,000.

**Figure 5:**
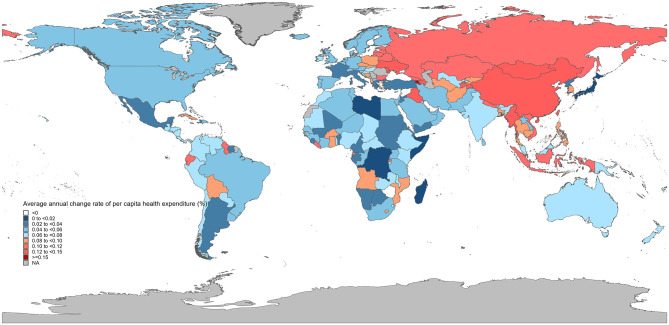
The average annual percentage change (AAPC) in per capita health expenditure across countries, 2000–2022.

The longitudinal analysis revealed equally striking disparities in cost trajectories (Figure 5, Supplementary Table S6). Armenia exhibited the most rapid expenditure growth (16.02% annual increase), closely followed by Republic of Moldova (14.22%) and Timor-Leste (13.73%). In contrast, Lebanon (−0.37%), Syrian Arab Republic (−1.99%), Zimbabwe (−2.39%) and Gambia (−4.23%) showed negative growth rates over the study period.

## Discussion

Our study presents an updated and comprehensive overview of global, regional, and national trends in cervical cancer burden across 204 countries and territories from 1991 to 2021. In 2021, the global burden of cervical cancer remained substantial, with 12 countries in North Africa and Middle East achieving fewer than 4 new cases per 100,000 women. Despite the significant decreases in global ASIR, ASMR and ASDR over the past 30 years, the ASPR has increased, particularly in high-middle and middle SDI regions. The onset risk shifted toward younger women, while the mortality risk remained relatively high in older women. The association between AAPC and SDI exhibited a threshold effect, with more pronounced AAPC decreases when SDI exceeded 0.60.

The upward prevalence of cervical cancer could be attributed to the high incidence in younger women and the high mortality in older women, potentially due to early detection through screening (22, 23). Globally, 139 of 202 countries and territories had issued screening guidelines up to 2021 with coverage ranging from 84% in high-income countries to 9% in lower-middle-income countries (24). However, insufficient screening coverage in low-middle and low SDI regions may have led to an underestimation of incidence. Advancements in cervical cancer therapies and improved survival, especially for early-stage cases (25, 26), could also contribute to the increasing prevalence. Also, the health care quality for cervical cancer was found to increase during 1990 to 2019 particularly for younger individuals (14).

North Africa and the Middle East had the lowest cervical cancer incidence [(4.72, 95% UI: 4.04, 5.50) per 100,000 people], with 12 out of 21 countries (Kuwait, Qatar, Saudi Arabia, Jordan, Oman, Iran, Iraq, Palestine, Syria, Egypt, Sudan, and Yemen) meeting the elimination threshold. However, this burden may be underestimated due to the lack of population-based cancer registries, wars disrupting cancer management, and insufficient data quality in countries such as Palestine, Yemen, Syria, Jordan, Iraq, and Iran (27–29). HPV infections are relatively low in this region, likely due to conservative sexual behaviors linked to religious beliefs, but there is no conclusive evidence that elimination targets have been met, warranting further assessment (30, 31). In contrast, Sub-Saharan Africa lacks well-established national cancer registries (32), and its ASPR, ASIR, ASMR, and ASDR for cervical cancer remain the highest globally and continue to rise (2, 33, 34). This suggests the actual burden may exceed current estimates. In addition to the high prevalence of HPV infection, human immunodeficiency virus (HIV) significantly contributes to cervical cancer risk in this region (35–37), with approximately 1 in 5 cases attributed to HIV, underscoring the need for improved HIV care and HPV monitoring among women living with HIV (38). The region also faces challenges in cervical cancer prevention and treatment, including low vaccine and screening coverage, delayed diagnosis, and poor adherence to treatment guidelines (39, 40). Despite efforts to promote HPV vaccination and screening, screening uptake was only 12.87% from 2000 to 2019, and HPV vaccines remain unavailable in many countries (41–43). Additionally, health education programs are needed to raise awareness among women and empower them to make informed healthcare decisions (44, 45).

In addition to the epidemiological indicators, our study provides the first systematic estimation of the direct medical cost of cervical cancer across 194 countries from 1990 to 2021. The global cumulative treatment-related burden reached approximately USD 9.26 billion without discounting, and USD 7.21 billion when applying a 3% annual discount rate. These substantial costs reflect not only the high incidence in lower SDI regions, but also the growing treatment needs associated with aging populations and delayed diagnoses. Importantly, this financial burden is concentrated in low- and middle-income countries, where public health budgets are already limited. It is important to note that our estimates may still be conservative. Our methodology is based on direct medical costs only and does not include indirect or non-medical expenditures such as productivity loss, transportation, or informal caregiving. We extrapolated unit costs from 2015 estimates, adjusted by national health expenditure growth rates, which may not capture heterogeneity in cancer care costs across settings (46). Despite these limitations, our findings establish a harmonized baseline for future cost monitoring and scenario modeling. This reinforces the urgency of scaling up cost-effective interventions such as HPV vaccination and cervical screening, which can avert both clinical and economic burden.

Between 1991 and 2021, the association between the AAPC, baseline rates, and SDI was consistent with previous findings (9, 11) . Australasia was among the regions with the lowest cervical cancer burden and the greatest reductions in all indicators. Both Australia and New Zealand have implemented nationwide cervical cancer screening since 1991 and 1990, respectively, and HPV vaccination programs since 2007 and 2008 (47, 48). These combined strategies have led to significant progress, with Australia predicted to be one of the first countries to reach cervical cancer elimination thresholds (49, 50). Differentiated prevention measures have been adopted for Indigenous women, who face a relatively higher burden (51). In China, the AAPC exceeded expectations, despite a low baseline ASIR in 1991, likely due to the launch of the cervical cancer screening program for millions of rural women in 2009. However, HPV vaccines were only approved in 2016, with a first-dose coverage of just 1.59% by 2021 (52–54). The Chinese government issued an Action Plan in 2023 to accelerate cervical cancer elimination, aiming to meet WHO's 2030 targets.

Our study, based on the latest GBD 2021 data, fills the gap in estimating prevalence cases and rates for cervical cancer during 1991–2021, revealing the necessity of proper resource allocation. The time trends in cervical cancer burden by age and SDI provide indirect evidence for the impact of HPV vaccination, screening, and treatment on cervical cancer elimination. There were some limitations in this study. First, the GBD database included multiple data sources, but the limited or low quality of available data in some regions may affect the estimated results of the study. Secondly, the data sources of the GBD study include vital registry, cancer registry and other sources. Countries with low development levels may lack these resources, and statistical data may be inaccurate. Third, our cost projection approach assumed that general health expenditure growth rates (from WHO GHED) reflect cervical cancer treatment cost trends over time. While country-specific rates capture national-level economic shifts, regional variations in medical inflation or disease-specific cost structures may not be fully represented. Future studies with access to localized cost data could refine these estimates.

## Conclusion

While global trends show decreasing incidence, mortality, and DALYs rates for cervical cancer, prevalence has risen from 1991 to 2021. Despite the cervical cancer elimination initiative launched in 2018, there has been little change in overall incidence and mortality as of 2021, suggesting a delay in meeting WHO's 2030 targets. Over the past three decades, higher incidence rates have been more pronounced among younger individuals, while mortality has gradually shifted toward older age groups. Significant disparities remain across SDI regions. Although 12 countries have achieved incidence rates below the elimination threshold, the burden in these nations may be underestimated. In Sub-Saharan Africa, all metrics have shown increasingly concerning trends, underscoring the need for tailored strategies in the future. As cervical cancer continues to impose not only health but also growing economic burdens—particularly in resource-constrained settings—investments in prevention, early detection, and universal access to care remain essential to meet global elimination targets.

## Data Availability

All data generated during this study are included in the Supplementary materials. The Global Burden of Disease datasets analyzed are publicly available at https://ghdx.healthdata.org/gbd-2021.

## References

[1] Cohen PA Jhingran A Oaknin A Denny L. Cervical cancer. Lancet. (2019) 393:169–82. 10.1016/S0140-6736(18)32470-X 30638582

[2] Bray F Parkin DM. Cancer in sub-Saharan Africa in 2020: a review of current estimates of the national burden, data gaps, and future needs. Lancet Oncol. (2022) 23:719–28. 10.1016/S1470-2045(22)00270-4 35550275

[3] Sung H Ferlay J Siegel RL Laversanne M Soerjomataram I Jemal A. Global Cancer Statistics 2020: GLOBOCAN Estimates of Incidence and Mortality Worldwide for 36 Cancers in 185 Countries. CA Cancer J Clin. (2021) 71:209–49. 10.3322/caac.21660 33538338

[4] Simms KT Steinberg J Caruana M Smith MA Lew JB Soerjomataram I. Impact of scaled up human papillomavirus vaccination and cervical screening and the potential for global elimination of cervical cancer in 181 countries, 2020-99: a modelling study. Lancet Oncol. (2019) 20:394–407. 10.1016/S1470-2045(18)30836-2 30795950

[5] Gultekin M Ramirez PT Broutet N Hutubessy R. World Health Organization call for action to eliminate cervical cancer globally. Int J Gynecol Cancer. (2020) 30:426–7. 10.1136/ijgc-2020-001285 32122950

[6] World Health Organization. (2020). Cervical Cancer Elimination Initiative [Online]. Available online at: https://www.who.int/initiatives/cervical-cancer-elimination-initiative (Accessed 2024).

[7] Canfell K Kim JJ Brisson M Keane A Simms KT Caruana M. Mortality impact of achieving WHO cervical cancer elimination targets: a comparative modelling analysis in 78 low-income and lower-middle-income countries. Lancet. (2020) 395:591–603. 10.1016/S0140-6736(20)30157-4 32007142 PMC7043006

[8] Singh D Vignat J Lorenzoni V Eslahi M Ginsburg O Lauby-Secretan B. Global estimates of incidence and mortality of cervical cancer in 2020: a baseline analysis of the WHO Global Cervical Cancer Elimination Initiative. Lancet Global Health. (2023) 11:e197–206. 10.1016/S2214-109X(22)00501-0 36528031 PMC9848409

[9] Zhao M Wu Q Hao Y Hu J Gao Y Zhou S. Global, regional, and national burden of cervical cancer for 195 countries and territories, 2007–2017: findings from the Global Burden of Disease Study 2017. BMC Women's Health. (2021) 21:419. 10.1186/s12905-021-01571-3 34922503 PMC8684284

[10] Yao H Yan C Qiumin H Li Z Jiao A Xin L. Epidemiological trends and attributable risk burden of cervical cancer: an observational study from 1990 to 2019. Int J Clin Pract. (2022) 2022:3356431. 10.1155/2022/3356431 36263235 PMC9546700

[11] Zhang X Zeng Q Cai W Ruan W. (2021) Trends of cervical cancer at global, regional, and national level: data from the Global Burden of Disease study 2019. BMC Public Health 21:894. 10.1186/s12889-021-10907-5 33975583 PMC8114503

[12] Momenimovahed Z Mazidimoradi A Maroofi P Allahqoli L Salehiniya H Alkatout I. Global, regional and national burden, incidence, and mortality of cervical cancer. Cancer Rep. (2023) 6:e1756. 10.1002/cnr2.1756 36545760 PMC10026270

[13] Wan Z Zhao J Xu L Sun P Shuai P Li K. Global and regional estimates of cervical cancer burden associated with human immunodeficiency virus infection from 1990 to 2019. J Med Virol. (2023) 95:e28891. 10.1002/jmv.28891 37338085

[14] Azangou-Khyavy M Ghasemi E Rezaei N Khanali J Kolahi AA Malekpour MR. Global, regional, and national quality of care index of cervical and ovarian cancer: a systematic analysis for the global burden of disease study 1990-2019. BMC Womens Health. (2024) 24:69. 10.1186/s12905-024-02884-9 38273304 PMC10809627

[15] Yang M Du J Lu H Xiang F Mei H Xiao H. Global trends and age-specific incidence and mortality of cervical cancer from 1990 to 2019: an international comparative study based on the Global Burden of Disease. BMJ Open. (2022) 12:e055470. 10.1136/bmjopen-2021-055470 35868828 PMC9316042

[16] Vaccarella S Lortet-Tieulent J Plummer M Franceschi S Bray F. Worldwide trends in cervical cancer incidence: impact of screening against changes in disease risk factors. Eur J Cancer. (2013) 49:3262–73. 10.1016/j.ejca.2013.04.024 23751569

[17] Fesenfeld M Hutubessy R Jit M. Cost-effectiveness of human papillomavirus vaccination in low and middle income countries: a systematic review. Vaccine. (2013) 31:3786–804. 10.1016/j.vaccine.2013.06.060 23830973

[18] GBD 2021 Diseases and Injuries Collaborators. (2024). Global incidence, prevalence, years lived with disability (YLDs), disability-adjusted life-years (DALYs), and healthy life expectancy (HALE) for 371 diseases and injuries in 204 countries and territories and 811 subnational locations, 1990-2021: a systematic analysis for the Global Burden of Disease Study 2021. Lancet. 403, 2133-2161. 38642570 10.1016/S0140-6736(24)00757-8 PMC11122111

[19] GBD 2021 Risk Factors Collaborators. (2024). Global burden and strength of evidence for 88 risk factors in 204 countries and 811 subnational locations, 1990-2021: a systematic analysis for the Global Burden of Disease Study 2021. Lancet. 403, 2162-2203. 38762324 10.1016/S0140-6736(24)00933-4 PMC11120204

[20] GBD 2021 Causes Of Death Collaborators. (2024). Global burden of 288 causes of death and life expectancy decomposition in 204 countries and territories and 811 subnational locations, 1990-2021: a systematic analysis for the Global Burden of Disease Study 2021. Lancet. 403, 2100-2132. 38582094 10.1016/S0140-6736(24)00367-2 PMC11126520

[21] World Health Organization. (2023). Tracking Universal Health Coverage: 2023 Global monitoring report. World Health Organization and the International Bank for Reconstruction and Development/The World Bank. Available online at: https://www.who.int/publications/i/item/9789240080379 (Accessed Accessed 5, 2024).

[22] Crosbie EJ Einstein MH Franceschi S Kitchener HC. Human papillomavirus and cervical cancer. Lancet. (2013) 382:889–99. 10.1016/S0140-6736(13)60022-7 23618600

[23] Bedell SL Goldstein LS Goldstein AR Goldstein AT. Cervical cancer screening: past, present, and future. Sex Med Rev. (2020) 8:28–37. 10.1016/j.sxmr.2019.09.005 31791846

[24] Bruni L Serrano B Roura E Alemany L Cowan M Herrero R. Cervical cancer screening programmes and age-specific coverage estimates for 202 countries and territories worldwide: a review and synthetic analysis. Lancet Glob Health. (2022) 10:e1115–27. 10.1016/S2214-109X(22)00241-8 35839811 PMC9296658

[25] Burmeister CA Khan SF Schäfer G Mbatani N Adams T Moodley J. Cervical cancer therapies: current challenges and future perspectives. Tumour Virus Res. (2022) 13:200238. 10.1016/j.tvr.2022.200238 35460940 PMC9062473

[26] Yagi A Ueda Y Kakuda M Tanaka Y Ikeda S Matsuzaki S. Epidemiologic and clinical analysis of cervical cancer using data from the population-based osaka cancer registry. Cancer Res. (2019) 79:1252–9. 10.1158/0008-5472.CAN-18-3109 30635276

[27] Siddiqui A. A. Amin J. Alshammary F. Afroze E. Shaikh S. Rathore H. A. Khan R. (2021). “Burden of Cancer in the Arab World,” In: Laher I. (ed.) Handbook of Healthcare in the Arab World. Cham: Springer International Publishing. 10.1007/978-3-030-36811-1_182

[28] Sancho-Garnier H Khazraji YC Cherif MH Mahnane A Hsairi M El Shalakamy A. Overview of cervical cancer screening practices in the extended Middle East and North Africa countries. Vaccine. (2013) 31 Suppl 6:G51–7. 10.1016/j.vaccine.2012.06.046 24331820

[29] Saeed IE Weng H-Y Mohamed KH Mohammed SI. Cancer incidence in Khartoum, Sudan: first results from the Cancer Registry, 2009-2010. Cancer Med. (2014) 3:1075–84. 10.1002/cam4.254 24821265 PMC4303176

[30] Brown R Kerr K Haoudi A Darzi A. Tackling cancer burden in the Middle East: Qatar as an example. Lancet Oncol. (2012) 13:e501–8. 10.1016/S1470-2045(12)70461-8 23084766

[31] Vaccarella S Bruni L Seoud M. Burden of human papillomavirus infections and related diseases in the extended Middle East and North Africa region. Vaccine. (2013) 31 Suppl 6:G32–44. 10.1016/j.vaccine.2012.06.098 24331818

[32] Crocker-Buque T Pollock AM. Appraising the quality of sub-Saharan African cancer registration systems that contributed to GLOBOCAN 2008: a review of the literature and critical appraisal. J Royal Soc Med. (2015) 108:57–67. 10.1177/0141076814554671 25721114 PMC4344444

[33] Mboumba Bouassa R-S Prazuck T Lethu T Jenabian M-A Meye J-F Bélec L. Cervical cancer in sub-Saharan Africa: a preventable noncommunicable disease. Expert Rev Anti Infect Ther. (2017) 15:613–27. 10.1080/14787210.2017.1322902 28440679

[34] Jedy-Agba E Joko WY Liu B Buziba NG Borok M Korir A. Trends in cervical cancer incidence in sub-Saharan Africa. Br J Cancer. (2020) 123:148–54. 10.1038/s41416-020-0831-9 32336751 PMC7341858

[35] Okoye JO Ofodile CA Adeleke OK Obioma O. Prevalence of high-risk HPV genotypes in sub-Saharan Africa according to HIV status: a 20-year systematic review. Epidemiol Health. (2021) 43:e2021039. 10.4178/epih.e2021039 34044477 PMC8510839

[36] Ibrahim Khalil A Mpunga T Wei F Baussano I De Martel C Bray F. Age-specific burden of cervical cancer associated with HIV: A global analysis with a focus on sub-Saharan Africa. Int J Cancer. (2022) 150:761–72. 10.1002/ijc.33841 34626498 PMC8732304

[37] De Martel C Plummer M Vignat J Franceschi S. Worldwide burden of cancer attributable to HPV by site, country and HPV type. Int J Cancer. (2017) 141:664–70. 10.1002/ijc.30716 28369882 PMC5520228

[38] Castle PE Einstein MH Sahasrabuddhe VV. Cervical cancer prevention and control in women living with human immunodeficiency virus. CA Cancer J Clin. (2021) 71:505–26. 10.3322/caac.21696 34499351 PMC10054840

[39] Griesel M Seraphin TP Mezger NCS Hämmerl L Feuchtner J Joko-Fru WY. Cervical Cancer in Sub-Saharan Africa: a multinational population-based cohort study of care and guideline adherence. Oncologist. (2021) 26:e807–16. 10.1002/onco.13718 33565668 PMC8100544

[40] Mcfarland DM Gueldner SM Mogobe KD. Integrated review of barriers to cervical cancer screening in Sub-Saharan Africa. J Nurs Scholarsh. (2016) 48:490–8. 10.1111/jnu.12232 27434871

[41] Yimer NB Mohammed MA Solomon K Tadese M Grutzmacher S Meikena HK. Cervical cancer screening uptake in Sub-Saharan Africa: a systematic review and meta-analysis. Public Health. (2021) 195:105–11. 10.1016/j.puhe.2021.04.014 34082174

[42] Ngcobo N Jaca A Iwu-Jaja CJ Mavundza E. Reflection: burden of cervical cancer in Sub-Saharan Africa and progress with HPV vaccination. Curr Opin Immunol. (2021) 71:21–6. 10.1016/j.coi.2021.03.006 33857884

[43] Black E Richmond R. Prevention of cervical cancer in Sub-Saharan Africa: the advantages and challenges of HPV vaccination. Vaccines. (2018) 6:61. 10.3390/vaccines6030061 30205561 PMC6161067

[44] Atnafu DD Khatri R Assefa Y. Drivers of cervical cancer prevention and management in sub-Saharan Africa: a qualitative synthesis of mixed studies. Health Res Policy Syst. (2024) 22:21. 10.1186/s12961-023-01094-3 38331830 PMC10851545

[45] Okyere J Aboagye RG Seidu AA Asare BY Mwamba B Ahinkorah BO. Towards a cervical cancer-free future: women's healthcare decision making and cervical cancer screening uptake in sub-Saharan Africa. BMJ Open. (2022) 12:e058026. 10.1136/bmjopen-2021-058026 35906053 PMC9345091

[46] Goldie SJ Gaffikin L Goldhaber-Fiebert JD Gordillo-Tobar A Levin C Mahé C. Cost-effectiveness of cervical-cancer screening in five developing countries. N Engl J Med. (2005) 353:2158–68. 10.1056/NEJMsa044278 16291985

[47] Smith MA Hall M Lew J-B Canfell K. Potential for HPV vaccination and primary HPV screening to reduce cervical cancer disparities: Example from New Zealand. Vaccine. (2018) 36:6314–24. 10.1016/j.vaccine.2018.08.063 30224201

[48] Patel C Brotherton JM Pillsbury A Jayasinghe S Donovan B Macartney K. The impact of 10 years of human papillomavirus (HPV) vaccination in Australia: what additional disease burden will a nonavalent vaccine prevent? Euro Surveill. (2018) 23. 10.2807/1560-7917.ES.2018.23.41.1700737 30326995 PMC6194907

[49] Crowe E Pandeya N Brotherton JML Dobson AJ Kisely S Lambert SB. Effectiveness of quadrivalent human papillomavirus vaccine for the prevention of cervical abnormalities: case-control study nested within a population based screening programme in Australia. BMJ. (2014) 348:g1458. 10.1136/bmj.g1458 24594809 PMC3942076

[50] Hall MT Simms KT Lew JB Smith MA Brotherton JM Saville M. The projected timeframe until cervical cancer elimination in Australia: a modelling study. Lancet Public Health. (2019) 4:e19–27. 10.1016/S2468-2667(18)30183-X 30291040

[51] Whop LJ Smith MA Butler TL Adcock A Bartholomew K Goodman MT. Achieving cervical cancer elimination among Indigenous women. Prevent Med. (2021) 144:106314. 10.1016/j.ypmed.2020.106314 33678228

[52] Yin Y. HPV vaccination in China needs to be more cost-effective. Lancet. (2017) 390:1735–6. 10.1016/S0140-6736(17)32606-5 29047442

[53] You D Han L Li L Hu J Zimet GD Alias H. Human Papillomavirus (HPV) vaccine uptake and the willingness to receive the hpv vaccination among female college students in China: a multicenter study. Vaccines. (2020) 8. 10.3390/vaccines8010031 31963370 PMC7157221

[54] Hu RY Liu LJ Zhang XX Zeng QM Xu CS Ye JK. [Current status of vaccination services for adults in urban and rural areas of nine provinces in China from 2019 to 2021]. Zhonghua Yu Fang Yi Xue Za Zhi [Chin J Prevent Med]. (2023) 57:2050–5. 10.3760/cma.j.cn112150-20230615-00468 38186155

