# Supplementary material for: Global, regional, and national disease burden and economic costs of cervical cancer (1991–2021): a multidimensional data synthesis analysis
Source: Front Public Health. 2025 Sep 11;13:1633975. doi: 10.3389/fpubh.2025.1633975 (PMC12461291; doi:10.3389/fpubh.2025.1633975)

**Global, regional, and national disease burden and economic costs of cervical cancer (1991–2021): a multidimensional data synthesis analysis**

Yidi Ma^1,2,3^, Xiaozhen Lai^1,2,3^, Hai Fang^1,2,3^

**Affiliation:**

1. Department of Health Policy and Management, School of Public Health, Peking University, Beijing, China.
2. China Center for Health Development Studies, Peking University, Beijing, China. [hfang@hsc.pku.edu.cn](mailto:hfang@hsc.pku.edu.cn).
3. Peking University Health Science Center-Chinese Center for Disease Control and Prevention Joint Center for Vaccine Economics, Peking University, Beijing, China. [hfang@hsc.pku.edu.cn](mailto:hfang@hsc.pku.edu.cn).

**Corresponding authors:**

**Hai Fang [hfang@hsc.pku.edu.cn](mailto:hfang@hsc.pku.edu.cn).**

**Xiaozhen Lai** laixiaozhen@pku.edu.cn

**Table S1** The estimated counts, age-standardized rates and crude rates of prevalence, incidence, mortality and DALY per 100 000 people for cervical cancer in 204 countries and territories in 2021.

|  | **Prevalence (95% UI)** | | |  | **Incidence (95% UI)** | | |  | **Mortality (95% UI)** | | |  | **DALY (95% UI)** | | |
| --- | --- | --- | --- | --- | --- | --- | --- | --- | --- | --- | --- | --- | --- | --- | --- |
|  | **Prevalent cases × 10^5** | **ASPR per 100 000 people** | **CPR per 100 000 people** |  | **Incident cases × 10^5** | **ASIR per 100 000 people** | **CIR per 100 000 people** |  | **Death cases × 10^5** | **ASMR per 100 000 people** | **CMR per 100 000 people** |  | **DALYs × 10^5** | **ASDR per 100 000 people** | **CDR per 100 000 people** |
| Afghanistan | 0.01 (0.01, 0.02) | 44.79 (23.58, 68.64) | 29.46 (15.71, 45.94) |  | 0.01 (0.01, 0.02) | 13.33 (7.09, 20.01) | 7.22 (3.75, 10.83) |  | 0.01 (0.00, 0.01) | 8.96 (4.87, 13.16) | 4.23 (2.20, 6.32) |  | 0.26 (0.13, 0.40) | 308.77 (160.75, 462.98) | 171.55 (88.21, 258.92) |
| Albania | 0.00 (0.00, 0.00) | 46.67 (29.91, 71.36) | 55.48 (36.43, 83.00) |  | 0.00 (0.00, 0.00) | 7.71 (5.12, 11.39) | 10.10 (6.73, 14.64) |  | 0.00 (0.00, 0.00) | 2.59 (1.76, 3.65) | 4.03 (2.78, 5.64) |  | 0.02 (0.01, 0.02) | 83.00 (55.10, 121.30) | 116.76 (78.67, 167.61) |
| Algeria | 0.02 (0.01, 0.02) | 34.59 (26.16, 46.02) | 37.24 (27.87, 49.45) |  | 0.02 (0.01, 0.02) | 7.43 (5.78, 9.54) | 7.25 (5.60, 9.38) |  | 0.01 (0.01, 0.01) | 3.69 (2.96, 4.59) | 3.14 (2.49, 3.95) |  | 0.24 (0.19, 0.31) | 115.40 (90.46, 145.83) | 111.90 (86.67, 143.22) |
| American Samoa | 0.00 (0.00, 0.00) | 104.54 (77.45, 137.25) | 109.99 (81.01, 144.17) |  | 0.00 (0.00, 0.00) | 21.97 (16.44, 27.98) | 22.73 (17.03, 29.01) |  | 0.00 (0.00, 0.00) | 10.51 (7.96, 13.40) | 10.44 (7.95, 13.38) |  | 0.00 (0.00, 0.00) | 332.27 (250.13, 425.07) | 350.46 (263.48, 449.64) |
| Andorra | 0.00 (0.00, 0.00) | 29.79 (18.39, 45.24) | 41.63 (25.80, 64.76) |  | 0.00 (0.00, 0.00) | 4.53 (2.92, 6.73) | 6.78 (4.43, 10.08) |  | 0.00 (0.00, 0.00) | 1.25 (0.86, 1.81) | 2.26 (1.58, 3.22) |  | 0.00 (0.00, 0.00) | 39.50 (26.21, 58.26) | 63.86 (43.00, 94.33) |
| Angola | 0.03 (0.02, 0.05) | 129.69 (82.45, 189.51) | 85.27 (53.43, 125.91) |  | 0.03 (0.02, 0.05) | 35.31 (23.39, 51.56) | 19.64 (12.82, 29.01) |  | 0.02 (0.01, 0.03) | 22.69 (15.19, 32.88) | 10.84 (7.24, 15.79) |  | 0.70 (0.45, 1.03) | 741.23 (492.89, 1082.65) | 414.16 (270.10, 614.09) |
| Antigua and Barbuda | 0.00 (0.00, 0.00) | 124.37 (112.54, 138.22) | 157.60 (142.15, 175.69) |  | 0.00 (0.00, 0.00) | 23.46 (21.61, 25.43) | 29.62 (27.27, 32.13) |  | 0.00 (0.00, 0.00) | 10.04 (9.39, 10.72) | 12.56 (11.71, 13.46) |  | 0.00 (0.00, 0.00) | 300.09 (279.39, 322.83) | 383.74 (355.88, 413.21) |
| Argentina | 0.07 (0.06, 0.07) | 140.65 (125.44, 156.12) | 154.98 (138.55, 171.88) |  | 0.07 (0.06, 0.07) | 24.66 (22.43, 27.27) | 28.35 (25.82, 31.30) |  | 0.03 (0.03, 0.03) | 9.59 (8.74, 10.54) | 11.86 (10.78, 13.10) |  | 0.91 (0.84, 1.00) | 337.13 (310.81, 368.72) | 391.01 (359.51, 428.07) |
| Armenia | 0.00 (0.00, 0.00) | 56.75 (48.00, 67.13) | 73.46 (62.59, 86.93) |  | 0.00 (0.00, 0.00) | 11.26 (9.84, 12.93) | 15.70 (13.79, 18.00) |  | 0.00 (0.00, 0.00) | 5.00 (4.43, 5.67) | 7.64 (6.78, 8.66) |  | 0.03 (0.03, 0.04) | 150.60 (132.48, 172.60) | 215.34 (190.03, 246.66) |
| Australia | 0.02 (0.01, 0.02) | 65.80 (58.25, 73.98) | 81.72 (72.86, 91.42) |  | 0.02 (0.01, 0.02) | 9.11 (8.14, 10.16) | 12.06 (10.78, 13.35) |  | 0.00 (0.00, 0.00) | 1.89 (1.67, 2.09) | 3.11 (2.73, 3.45) |  | 0.11 (0.10, 0.13) | 62.84 (56.84, 69.52) | 87.52 (78.92, 96.84) |
| Austria | 0.00 (0.00, 0.01) | 42.57 (37.87, 47.33) | 57.06 (50.98, 63.16) |  | 0.00 (0.00, 0.01) | 6.75 (6.06, 7.44) | 10.46 (9.29, 11.48) |  | 0.00 (0.00, 0.00) | 2.23 (1.97, 2.44) | 4.51 (3.93, 4.98) |  | 0.05 (0.04, 0.06) | 66.98 (60.78, 72.77) | 110.61 (98.81, 121.16) |
| Azerbaijan | 0.01 (0.00, 0.01) | 41.99 (31.59, 53.84) | 51.66 (38.66, 66.24) |  | 0.01 (0.00, 0.01) | 8.73 (6.60, 11.01) | 10.48 (7.90, 13.31) |  | 0.00 (0.00, 0.00) | 4.11 (3.17, 5.12) | 4.73 (3.62, 5.93) |  | 0.08 (0.06, 0.10) | 131.56 (99.64, 164.57) | 159.38 (119.78, 199.30) |
| Bahamas | 0.00 (0.00, 0.00) | 150.20 (113.54, 191.32) | 176.17 (133.48, 223.93) |  | 0.00 (0.00, 0.00) | 27.04 (20.99, 34.17) | 31.53 (24.45, 39.88) |  | 0.00 (0.00, 0.00) | 10.82 (8.56, 13.66) | 12.43 (9.78, 15.74) |  | 0.01 (0.01, 0.01) | 370.40 (285.02, 473.09) | 437.53 (336.44, 559.74) |
| Bahrain | 0.00 (0.00, 0.00) | 18.99 (14.22, 24.85) | 20.88 (15.52, 27.83) |  | 0.00 (0.00, 0.00) | 4.65 (3.59, 6.06) | 4.13 (3.15, 5.42) |  | 0.00 (0.00, 0.00) | 2.44 (1.88, 3.17) | 1.70 (1.28, 2.24) |  | 0.00 (0.00, 0.00) | 66.81 (51.07, 87.89) | 58.03 (43.61, 76.64) |
| Bangladesh | 0.11 (0.08, 0.16) | 63.23 (42.21, 92.10) | 62.96 (41.88, 92.22) |  | 0.11 (0.08, 0.16) | 14.41 (9.88, 20.68) | 13.50 (9.23, 19.54) |  | 0.06 (0.04, 0.08) | 7.72 (5.43, 10.66) | 6.73 (4.76, 9.38) |  | 1.98 (1.38, 2.83) | 253.83 (177.11, 360.37) | 236.62 (164.39, 338.61) |
| Barbados | 0.00 (0.00, 0.00) | 174.54 (132.91, 228.41) | 231.97 (177.29, 303.42) |  | 0.00 (0.00, 0.00) | 31.43 (24.44, 39.88) | 46.38 (36.35, 58.78) |  | 0.00 (0.00, 0.00) | 12.79 (10.16, 15.94) | 21.64 (17.18, 26.83) |  | 0.01 (0.01, 0.01) | 388.97 (304.08, 488.00) | 592.37 (466.06, 741.29) |
| Belarus | 0.01 (0.01, 0.01) | 86.93 (66.96, 111.38) | 111.94 (86.75, 142.36) |  | 0.01 (0.01, 0.01) | 13.54 (10.58, 17.04) | 19.00 (15.05, 23.72) |  | 0.00 (0.00, 0.00) | 4.66 (3.74, 5.73) | 7.80 (6.31, 9.47) |  | 0.12 (0.10, 0.15) | 167.40 (131.94, 209.70) | 246.56 (196.04, 304.39) |
| Belgium | 0.01 (0.01, 0.01) | 51.90 (45.35, 59.17) | 63.76 (56.48, 72.25) |  | 0.01 (0.01, 0.01) | 7.89 (6.97, 8.87) | 11.24 (10.05, 12.49) |  | 0.00 (0.00, 0.00) | 2.39 (2.14, 2.65) | 4.69 (4.04, 5.21) |  | 0.07 (0.06, 0.07) | 74.33 (67.53, 82.50) | 115.72 (104.18, 128.11) |
| Belize | 0.00 (0.00, 0.00) | 204.11 (173.53, 236.72) | 192.10 (161.99, 222.68) |  | 0.00 (0.00, 0.00) | 39.66 (34.35, 45.27) | 34.65 (29.73, 39.70) |  | 0.00 (0.00, 0.00) | 17.63 (15.51, 20.00) | 13.83 (12.12, 15.80) |  | 0.01 (0.01, 0.01) | 585.49 (511.62, 669.96) | 506.00 (441.09, 580.90) |
| Benin | 0.01 (0.01, 0.01) | 86.78 (60.44, 117.61) | 56.90 (38.90, 77.54) |  | 0.01 (0.01, 0.01) | 25.14 (18.23, 33.01) | 13.64 (9.62, 17.98) |  | 0.01 (0.00, 0.01) | 16.63 (12.21, 21.08) | 7.71 (5.62, 9.96) |  | 0.19 (0.14, 0.25) | 515.22 (373.07, 668.97) | 278.76 (197.02, 369.74) |
| Bermuda | 0.00 (0.00, 0.00) | 76.49 (60.94, 97.51) | 107.09 (85.72, 137.23) |  | 0.00 (0.00, 0.00) | 11.51 (9.29, 14.63) | 18.30 (14.87, 23.29) |  | 0.00 (0.00, 0.00) | 3.26 (2.67, 4.15) | 6.86 (5.58, 8.68) |  | 0.00 (0.00, 0.00) | 100.68 (81.30, 127.20) | 171.97 (140.51, 217.55) |
| Bhutan | 0.00 (0.00, 0.00) | 65.43 (41.01, 97.60) | 65.23 (40.46, 98.10) |  | 0.00 (0.00, 0.00) | 15.64 (10.25, 21.75) | 14.50 (9.40, 20.60) |  | 0.00 (0.00, 0.00) | 8.87 (6.00, 12.01) | 7.65 (5.12, 10.36) |  | 0.01 (0.01, 0.01) | 282.09 (184.67, 386.27) | 257.94 (167.82, 356.87) |
| Bolivia (Plurinational State of) | 0.02 (0.01, 0.03) | 167.08 (112.18, 241.80) | 156.75 (105.29, 226.87) |  | 0.02 (0.01, 0.03) | 36.84 (25.27, 51.58) | 32.91 (22.50, 46.66) |  | 0.01 (0.01, 0.02) | 22.05 (15.03, 30.41) | 18.54 (12.56, 25.84) |  | 0.34 (0.23, 0.49) | 653.00 (447.56, 923.07) | 583.25 (397.75, 829.92) |
| Bosnia and Herzegovina | 0.00 (0.00, 0.00) | 78.55 (54.92, 103.36) | 102.62 (72.43, 135.03) |  | 0.00 (0.00, 0.00) | 13.51 (9.64, 17.47) | 19.99 (14.61, 25.59) |  | 0.00 (0.00, 0.00) | 4.68 (3.46, 6.02) | 8.49 (6.38, 10.85) |  | 0.04 (0.03, 0.05) | 150.90 (109.12, 195.76) | 240.30 (175.81, 311.20) |
| Botswana | 0.00 (0.00, 0.00) | 98.79 (63.55, 159.76) | 95.62 (60.50, 154.94) |  | 0.00 (0.00, 0.00) | 28.31 (19.18, 44.69) | 23.75 (15.75, 39.07) |  | 0.00 (0.00, 0.00) | 18.31 (12.81, 28.12) | 13.55 (9.33, 21.13) |  | 0.06 (0.04, 0.09) | 545.12 (369.99, 862.41) | 457.78 (300.37, 748.57) |
| Brazil | 0.27 (0.25, 0.28) | 107.73 (101.98, 113.52) | 125.80 (119.03, 132.56) |  | 0.27 (0.25, 0.28) | 19.90 (18.79, 20.90) | 23.60 (22.27, 24.81) |  | 0.11 (0.11, 0.12) | 8.21 (7.68, 8.66) | 9.97 (9.32, 10.53) |  | 3.78 (3.56, 3.97) | 280.03 (263.77, 293.69) | 335.27 (315.70, 351.57) |
| Brunei Darussalam | 0.00 (0.00, 0.00) | 95.62 (73.53, 122.04) | 112.49 (86.04, 143.77) |  | 0.00 (0.00, 0.00) | 17.94 (14.11, 22.45) | 19.33 (14.98, 24.44) |  | 0.00 (0.00, 0.00) | 7.90 (6.32, 9.67) | 7.50 (5.88, 9.34) |  | 0.01 (0.00, 0.01) | 252.38 (197.42, 315.27) | 271.72 (209.66, 344.55) |
| Bulgaria | 0.01 (0.01, 0.01) | 139.45 (112.21, 168.13) | 179.21 (145.16, 214.46) |  | 0.01 (0.01, 0.01) | 23.22 (18.95, 27.63) | 34.18 (27.87, 40.27) |  | 0.01 (0.00, 0.01) | 7.80 (6.43, 9.17) | 14.41 (11.89, 17.00) |  | 0.15 (0.12, 0.17) | 270.98 (222.41, 319.23) | 423.32 (347.65, 498.60) |
| Burkina Faso | 0.02 (0.01, 0.02) | 100.23 (72.11, 133.93) | 66.45 (47.00, 91.24) |  | 0.02 (0.01, 0.02) | 28.84 (21.39, 37.42) | 15.91 (11.66, 20.91) |  | 0.01 (0.01, 0.01) | 19.50 (14.65, 25.04) | 9.26 (6.89, 11.94) |  | 0.39 (0.29, 0.51) | 606.51 (446.44, 781.04) | 335.06 (244.99, 433.84) |
| Burundi | 0.01 (0.01, 0.02) | 115.80 (78.42, 163.41) | 73.05 (49.41, 103.46) |  | 0.01 (0.01, 0.02) | 34.89 (24.13, 49.08) | 18.09 (12.45, 25.54) |  | 0.01 (0.00, 0.01) | 23.76 (16.59, 33.30) | 10.57 (7.23, 15.01) |  | 0.27 (0.19, 0.39) | 784.95 (534.94, 1117.82) | 409.24 (281.52, 587.75) |
| Cabo Verde | 0.00 (0.00, 0.00) | 83.51 (61.37, 111.45) | 83.54 (60.68, 111.99) |  | 0.00 (0.00, 0.00) | 20.71 (15.89, 26.79) | 19.83 (15.21, 25.43) |  | 0.00 (0.00, 0.00) | 12.59 (9.72, 16.05) | 11.67 (9.04, 14.77) |  | 0.01 (0.01, 0.01) | 335.59 (258.37, 433.85) | 318.09 (245.09, 410.87) |
| Cambodia | 0.01 (0.01, 0.02) | 78.41 (55.79, 108.46) | 76.69 (54.33, 106.20) |  | 0.01 (0.01, 0.02) | 17.05 (12.51, 23.86) | 16.01 (11.68, 22.30) |  | 0.01 (0.01, 0.01) | 9.45 (7.08, 13.17) | 8.45 (6.26, 11.78) |  | 0.26 (0.19, 0.36) | 314.19 (230.11, 439.72) | 295.44 (215.36, 412.95) |
| Cameroon | 0.03 (0.02, 0.04) | 114.01 (69.09, 162.68) | 80.80 (47.23, 114.66) |  | 0.03 (0.02, 0.04) | 31.45 (20.79, 44.42) | 18.33 (11.55, 26.03) |  | 0.02 (0.01, 0.02) | 19.97 (13.34, 28.45) | 9.72 (6.29, 13.87) |  | 0.58 (0.36, 0.82) | 630.37 (406.04, 900.93) | 364.27 (227.19, 516.11) |
| Canada | 0.03 (0.03, 0.03) | 99.77 (88.15, 111.02) | 115.91 (103.63, 128.28) |  | 0.03 (0.03, 0.03) | 13.23 (11.78, 14.59) | 16.57 (14.94, 18.07) |  | 0.01 (0.01, 0.01) | 2.28 (2.10, 2.47) | 4.00 (3.61, 4.37) |  | 0.22 (0.20, 0.24) | 79.47 (73.03, 86.63) | 114.12 (105.06, 123.90) |
| Central African Republic | 0.01 (0.00, 0.01) | 131.71 (84.73, 192.04) | 93.51 (59.43, 137.89) |  | 0.01 (0.00, 0.01) | 44.83 (29.91, 63.82) | 27.05 (17.64, 38.91) |  | 0.00 (0.00, 0.01) | 33.02 (22.16, 47.70) | 17.82 (11.73, 26.00) |  | 0.19 (0.12, 0.28) | 1096.73 (718.76, 1601.52) | 682.73 (444.83, 1001.21) |
| Chad | 0.01 (0.01, 0.02) | 105.99 (73.63, 144.78) | 57.35 (38.95, 80.44) |  | 0.01 (0.01, 0.02) | 34.27 (24.90, 45.34) | 14.81 (10.47, 19.78) |  | 0.01 (0.01, 0.01) | 24.23 (18.03, 32.06) | 8.88 (6.47, 11.81) |  | 0.30 (0.21, 0.40) | 764.57 (554.09, 1019.00) | 335.05 (238.31, 453.20) |
| Chile | 0.02 (0.02, 0.02) | 113.82 (100.13, 129.32) | 132.42 (117.19, 149.52) |  | 0.02 (0.02, 0.02) | 18.50 (16.49, 20.60) | 22.85 (20.53, 25.34) |  | 0.01 (0.01, 0.01) | 6.29 (5.76, 6.84) | 8.74 (7.99, 9.53) |  | 0.25 (0.23, 0.28) | 206.72 (188.19, 226.83) | 264.19 (242.25, 290.58) |
| China | 1.33 (0.96, 1.73) | 79.03 (56.34, 103.91) | 107.47 (76.71, 141.52) |  | 1.33 (0.96, 1.73) | 13.37 (9.61, 17.51) | 19.12 (13.81, 24.85) |  | 0.50 (0.37, 0.64) | 4.64 (3.44, 6.00) | 7.18 (5.31, 9.27) |  | 15.48 (11.20, 20.09) | 149.84 (108.94, 195.58) | 222.82 (161.21, 289.25) |
| Colombia | 0.08 (0.07, 0.10) | 167.25 (135.33, 206.36) | 187.81 (152.26, 231.02) |  | 0.08 (0.07, 0.10) | 28.50 (23.20, 34.55) | 32.58 (26.64, 39.42) |  | 0.02 (0.02, 0.03) | 7.87 (6.51, 9.41) | 9.32 (7.73, 11.12) |  | 0.78 (0.64, 0.94) | 268.41 (221.23, 324.95) | 310.32 (255.95, 374.71) |
| Comoros | 0.00 (0.00, 0.00) | 140.53 (90.99, 202.55) | 127.52 (82.82, 183.61) |  | 0.00 (0.00, 0.00) | 38.16 (25.39, 53.63) | 32.03 (21.25, 45.05) |  | 0.00 (0.00, 0.00) | 24.05 (15.80, 34.20) | 18.84 (12.37, 26.88) |  | 0.03 (0.02, 0.04) | 796.53 (526.96, 1142.51) | 672.17 (442.55, 960.05) |
| Congo | 0.01 (0.01, 0.01) | 180.76 (98.33, 270.62) | 154.68 (82.20, 232.67) |  | 0.01 (0.01, 0.01) | 47.09 (27.40, 70.25) | 34.90 (19.52, 52.45) |  | 0.01 (0.00, 0.01) | 28.75 (17.08, 42.85) | 18.53 (10.70, 28.09) |  | 0.19 (0.11, 0.29) | 960.14 (552.76, 1461.53) | 711.96 (399.67, 1076.92) |
| Cook Islands | 0.00 (0.00, 0.00) | 50.86 (36.17, 70.46) | 59.99 (43.29, 82.18) |  | 0.00 (0.00, 0.00) | 8.94 (6.61, 12.05) | 11.22 (8.36, 15.11) |  | 0.00 (0.00, 0.00) | 3.35 (2.50, 4.36) | 4.62 (3.46, 6.02) |  | 0.00 (0.00, 0.00) | 100.75 (73.99, 135.18) | 130.57 (95.73, 174.48) |
| Costa Rica | 0.01 (0.01, 0.01) | 147.37 (123.70, 172.35) | 171.42 (144.06, 200.59) |  | 0.01 (0.01, 0.01) | 24.27 (20.72, 27.93) | 28.51 (24.40, 32.73) |  | 0.00 (0.00, 0.00) | 6.28 (5.48, 7.17) | 7.55 (6.59, 8.62) |  | 0.06 (0.05, 0.07) | 212.36 (183.16, 241.65) | 250.78 (216.20, 285.87) |
| Croatia | 0.00 (0.00, 0.01) | 91.03 (69.47, 116.80) | 129.15 (98.89, 164.30) |  | 0.00 (0.00, 0.01) | 13.31 (10.21, 16.84) | 21.15 (16.30, 26.69) |  | 0.00 (0.00, 0.00) | 3.51 (2.74, 4.35) | 7.27 (5.77, 8.95) |  | 0.04 (0.03, 0.05) | 109.25 (83.89, 136.74) | 185.32 (144.02, 228.66) |
| Cuba | 0.02 (0.02, 0.02) | 137.85 (112.45, 166.27) | 182.57 (148.64, 218.38) |  | 0.02 (0.02, 0.02) | 22.42 (18.50, 26.55) | 32.33 (26.86, 38.14) |  | 0.01 (0.01, 0.01) | 7.51 (6.32, 8.80) | 12.69 (10.67, 14.76) |  | 0.21 (0.17, 0.25) | 243.27 (203.96, 288.14) | 367.96 (308.56, 433.96) |
| Cyprus | 0.00 (0.00, 0.00) | 39.83 (31.34, 49.89) | 53.98 (42.61, 67.72) |  | 0.00 (0.00, 0.00) | 6.43 (5.15, 7.89) | 9.05 (7.19, 11.08) |  | 0.00 (0.00, 0.00) | 2.31 (1.84, 2.83) | 3.45 (2.75, 4.25) |  | 0.01 (0.00, 0.01) | 62.64 (50.07, 76.05) | 90.37 (72.13, 110.33) |
| Czechia | 0.01 (0.01, 0.01) | 56.16 (46.44, 66.89) | 73.04 (60.77, 86.32) |  | 0.01 (0.01, 0.01) | 9.60 (8.13, 11.29) | 14.73 (12.48, 17.39) |  | 0.00 (0.00, 0.00) | 3.70 (3.11, 4.40) | 7.26 (6.03, 8.71) |  | 0.10 (0.09, 0.12) | 113.49 (95.90, 134.41) | 187.70 (157.83, 223.00) |
| Cote d'Ivoire | 0.01 (0.01, 0.02) | 55.84 (35.75, 79.95) | 38.76 (25.04, 56.19) |  | 0.01 (0.01, 0.02) | 15.87 (10.54, 22.25) | 9.03 (5.93, 12.75) |  | 0.01 (0.00, 0.01) | 10.20 (6.96, 14.10) | 4.87 (3.20, 6.89) |  | 0.24 (0.16, 0.35) | 320.70 (210.10, 453.02) | 181.11 (115.72, 259.67) |
| Democratic People's Republic of Korea | 0.03 (0.02, 0.04) | 94.85 (61.31, 133.70) | 119.38 (77.48, 166.97) |  | 0.03 (0.02, 0.04) | 17.45 (11.82, 24.70) | 22.85 (15.60, 32.27) |  | 0.01 (0.01, 0.02) | 7.60 (5.30, 10.91) | 10.52 (7.40, 15.18) |  | 0.47 (0.32, 0.68) | 268.44 (182.00, 385.08) | 357.44 (242.85, 513.76) |
| Democratic Republic of the Congo | 0.10 (0.07, 0.14) | 131.15 (83.63, 189.43) | 89.11 (56.12, 128.36) |  | 0.10 (0.07, 0.14) | 37.92 (25.03, 53.74) | 22.27 (14.65, 31.49) |  | 0.06 (0.04, 0.08) | 25.34 (16.85, 35.65) | 13.21 (8.73, 18.72) |  | 2.15 (1.41, 3.08) | 816.07 (538.56, 1159.07) | 483.16 (315.54, 691.49) |
| Denmark | 0.00 (0.00, 0.00) | 53.38 (47.09, 60.62) | 62.07 (55.11, 69.51) |  | 0.00 (0.00, 0.00) | 8.47 (7.61, 9.39) | 11.93 (10.72, 13.09) |  | 0.00 (0.00, 0.00) | 2.88 (2.58, 3.15) | 5.74 (5.01, 6.35) |  | 0.04 (0.04, 0.04) | 84.21 (76.05, 91.58) | 134.08 (121.56, 145.71) |
| Djibouti | 0.00 (0.00, 0.00) | 124.32 (73.60, 193.65) | 112.94 (65.89, 180.64) |  | 0.00 (0.00, 0.00) | 34.71 (21.48, 52.64) | 26.39 (16.05, 40.59) |  | 0.00 (0.00, 0.00) | 22.17 (14.03, 33.11) | 14.35 (8.80, 21.99) |  | 0.03 (0.02, 0.05) | 713.65 (434.79, 1093.97) | 544.90 (331.49, 853.17) |
| Dominica | 0.00 (0.00, 0.00) | 149.58 (109.48, 197.98) | 170.81 (125.22, 225.16) |  | 0.00 (0.00, 0.00) | 31.42 (23.66, 40.48) | 37.97 (28.64, 48.83) |  | 0.00 (0.00, 0.00) | 15.50 (12.01, 19.67) | 19.92 (15.50, 25.26) |  | 0.00 (0.00, 0.00) | 480.09 (364.90, 623.86) | 584.23 (444.59, 754.77) |
| Dominican Republic | 0.01 (0.01, 0.02) | 114.27 (82.05, 153.39) | 116.16 (83.62, 156.02) |  | 0.01 (0.01, 0.02) | 22.40 (16.19, 29.68) | 22.31 (16.17, 29.49) |  | 0.01 (0.00, 0.01) | 10.30 (7.55, 13.42) | 10.00 (7.34, 13.04) |  | 0.18 (0.13, 0.24) | 338.56 (245.31, 446.16) | 335.86 (243.93, 443.01) |
| Ecuador | 0.02 (0.02, 0.03) | 132.02 (98.53, 171.92) | 131.10 (97.86, 170.84) |  | 0.02 (0.02, 0.03) | 23.78 (18.21, 30.20) | 23.34 (17.88, 29.67) |  | 0.01 (0.01, 0.01) | 11.25 (8.75, 14.06) | 10.80 (8.39, 13.51) |  | 0.31 (0.24, 0.39) | 346.85 (266.20, 441.46) | 338.71 (259.74, 431.92) |
| Egypt | 0.01 (0.01, 0.02) | 12.33 (8.74, 16.29) | 10.83 (7.69, 14.43) |  | 0.01 (0.01, 0.02) | 3.63 (2.69, 4.71) | 2.62 (1.89, 3.44) |  | 0.01 (0.00, 0.01) | 2.29 (1.71, 2.86) | 1.37 (0.98, 1.78) |  | 0.24 (0.17, 0.31) | 63.91 (46.38, 82.19) | 46.36 (33.11, 60.59) |
| El Salvador | 0.02 (0.01, 0.02) | 239.69 (177.07, 310.54) | 243.37 (179.47, 314.45) |  | 0.02 (0.01, 0.02) | 45.54 (34.26, 58.40) | 46.54 (35.03, 59.65) |  | 0.01 (0.00, 0.01) | 15.36 (11.91, 19.26) | 16.02 (12.41, 20.14) |  | 0.17 (0.13, 0.22) | 496.03 (378.07, 622.92) | 507.20 (387.04, 637.42) |
| Equatorial Guinea | 0.00 (0.00, 0.00) | 155.58 (83.19, 255.96) | 117.07 (60.90, 193.36) |  | 0.00 (0.00, 0.00) | 36.25 (20.29, 57.84) | 22.99 (12.53, 37.48) |  | 0.00 (0.00, 0.00) | 20.39 (12.07, 32.33) | 10.59 (5.88, 17.07) |  | 0.03 (0.01, 0.05) | 659.20 (368.14, 1063.19) | 406.43 (216.44, 666.63) |
| Eritrea | 0.01 (0.01, 0.02) | 159.21 (97.62, 238.75) | 123.08 (75.79, 187.85) |  | 0.01 (0.01, 0.02) | 48.85 (30.69, 71.24) | 32.90 (20.56, 48.51) |  | 0.01 (0.00, 0.01) | 32.97 (20.78, 47.02) | 19.89 (12.35, 28.70) |  | 0.24 (0.15, 0.36) | 1097.51 (679.21, 1591.32) | 750.26 (465.82, 1096.42) |
| Estonia | 0.00 (0.00, 0.00) | 99.64 (82.04, 117.51) | 132.25 (110.79, 154.76) |  | 0.00 (0.00, 0.00) | 15.43 (12.98, 17.98) | 23.96 (20.40, 27.91) |  | 0.00 (0.00, 0.00) | 4.56 (3.88, 5.28) | 9.41 (8.04, 10.88) |  | 0.02 (0.01, 0.02) | 143.88 (120.64, 167.22) | 234.68 (198.80, 272.14) |
| Eswatini | 0.00 (0.00, 0.00) | 179.84 (93.98, 300.45) | 143.35 (73.45, 243.69) |  | 0.00 (0.00, 0.00) | 53.27 (28.74, 84.26) | 37.22 (19.71, 59.69) |  | 0.00 (0.00, 0.00) | 34.62 (19.25, 53.61) | 21.64 (11.72, 33.98) |  | 0.04 (0.02, 0.07) | 1089.98 (584.58, 1729.53) | 760.23 (399.72, 1231.18) |
| Ethiopia | 0.08 (0.06, 0.12) | 91.96 (66.26, 136.98) | 60.46 (43.12, 91.18) |  | 0.08 (0.06, 0.12) | 26.94 (20.07, 38.59) | 14.59 (10.66, 21.78) |  | 0.04 (0.03, 0.06) | 17.92 (13.56, 25.08) | 8.30 (6.21, 11.84) |  | 1.63 (1.20, 2.39) | 559.13 (415.69, 800.41) | 301.26 (221.91, 442.55) |
| Fiji | 0.00 (0.00, 0.00) | 185.53 (128.95, 254.81) | 189.15 (131.22, 259.63) |  | 0.00 (0.00, 0.00) | 42.64 (29.70, 57.21) | 41.52 (28.95, 55.91) |  | 0.00 (0.00, 0.00) | 22.33 (15.73, 29.35) | 20.29 (14.16, 27.34) |  | 0.03 (0.02, 0.04) | 704.07 (489.82, 944.25) | 693.28 (482.93, 934.13) |
| Finland | 0.00 (0.00, 0.00) | 38.15 (33.32, 43.33) | 46.26 (40.61, 52.43) |  | 0.00 (0.00, 0.00) | 5.44 (4.78, 6.10) | 7.75 (6.82, 8.69) |  | 0.00 (0.00, 0.00) | 1.35 (1.19, 1.50) | 2.88 (2.45, 3.25) |  | 0.02 (0.02, 0.02) | 43.40 (38.76, 48.11) | 68.58 (60.76, 76.28) |
| France | 0.04 (0.04, 0.04) | 53.32 (47.66, 60.05) | 64.11 (57.35, 71.65) |  | 0.04 (0.04, 0.04) | 8.05 (7.28, 9.01) | 11.57 (10.40, 12.69) |  | 0.02 (0.01, 0.02) | 2.42 (2.16, 2.67) | 5.10 (4.35, 5.72) |  | 0.40 (0.36, 0.44) | 74.05 (67.55, 81.05) | 117.56 (106.04, 129.41) |
| Gabon | 0.00 (0.00, 0.00) | 133.72 (82.72, 207.84) | 109.64 (67.37, 171.86) |  | 0.00 (0.00, 0.00) | 33.24 (21.13, 49.91) | 24.37 (15.24, 37.06) |  | 0.00 (0.00, 0.00) | 19.74 (12.91, 28.90) | 12.91 (8.30, 19.10) |  | 0.04 (0.03, 0.07) | 624.87 (400.64, 934.44) | 457.35 (291.10, 689.97) |
| Gambia | 0.00 (0.00, 0.00) | 106.87 (68.56, 147.90) | 76.58 (46.94, 109.57) |  | 0.00 (0.00, 0.00) | 27.33 (18.70, 36.82) | 16.44 (10.57, 22.61) |  | 0.00 (0.00, 0.00) | 16.22 (11.50, 21.74) | 8.23 (5.66, 11.14) |  | 0.04 (0.03, 0.05) | 545.41 (370.91, 745.47) | 320.42 (211.52, 448.02) |
| Georgia | 0.00 (0.00, 0.01) | 83.88 (70.40, 98.30) | 105.84 (88.87, 124.28) |  | 0.00 (0.00, 0.01) | 16.88 (14.41, 19.48) | 24.21 (20.76, 27.81) |  | 0.00 (0.00, 0.00) | 7.63 (6.60, 8.76) | 12.72 (11.02, 14.65) |  | 0.07 (0.06, 0.08) | 244.44 (209.20, 282.13) | 363.77 (312.08, 418.44) |
| Germany | 0.06 (0.05, 0.06) | 64.09 (56.56, 72.49) | 82.52 (73.56, 91.37) |  | 0.06 (0.05, 0.06) | 9.31 (8.35, 10.31) | 13.79 (12.34, 15.04) |  | 0.02 (0.02, 0.03) | 2.61 (2.37, 2.83) | 5.37 (4.73, 5.94) |  | 0.59 (0.53, 0.64) | 85.17 (77.28, 92.34) | 137.98 (124.49, 150.20) |
| Ghana | 0.04 (0.02, 0.05) | 113.65 (77.50, 151.17) | 93.94 (64.08, 125.61) |  | 0.04 (0.02, 0.05) | 29.20 (19.98, 38.18) | 20.70 (14.15, 27.29) |  | 0.02 (0.01, 0.03) | 17.99 (12.46, 23.59) | 10.96 (7.51, 14.38) |  | 0.70 (0.47, 0.92) | 563.21 (382.99, 741.79) | 396.74 (268.00, 521.74) |
| Greece | 0.01 (0.01, 0.01) | 52.50 (47.85, 57.23) | 70.20 (64.25, 76.73) |  | 0.01 (0.01, 0.01) | 8.28 (7.58, 8.93) | 13.28 (12.07, 14.34) |  | 0.00 (0.00, 0.00) | 2.78 (2.53, 2.99) | 6.24 (5.50, 6.80) |  | 0.08 (0.07, 0.08) | 83.72 (77.68, 89.35) | 146.00 (133.36, 157.19) |
| Greenland | 0.00 (0.00, 0.00) | 138.88 (96.47, 189.37) | 157.62 (111.31, 213.11) |  | 0.00 (0.00, 0.00) | 22.77 (16.59, 30.60) | 26.54 (19.62, 35.60) |  | 0.00 (0.00, 0.00) | 7.59 (5.95, 9.94) | 9.24 (7.21, 12.29) |  | 0.00 (0.00, 0.00) | 268.57 (204.62, 355.49) | 327.44 (250.60, 435.40) |
| Grenada | 0.00 (0.00, 0.00) | 201.11 (167.89, 243.07) | 224.37 (188.68, 269.51) |  | 0.00 (0.00, 0.00) | 39.17 (33.26, 46.33) | 44.85 (38.40, 53.12) |  | 0.00 (0.00, 0.00) | 17.46 (15.17, 20.27) | 20.60 (17.92, 23.90) |  | 0.00 (0.00, 0.00) | 557.21 (470.99, 660.01) | 648.18 (552.31, 769.58) |
| Guam | 0.00 (0.00, 0.00) | 72.32 (59.77, 84.88) | 81.82 (68.00, 95.76) |  | 0.00 (0.00, 0.00) | 12.48 (10.45, 14.39) | 14.93 (12.54, 17.20) |  | 0.00 (0.00, 0.00) | 4.43 (3.66, 5.12) | 5.81 (4.76, 6.75) |  | 0.00 (0.00, 0.00) | 158.91 (135.70, 182.26) | 195.08 (163.56, 224.37) |
| Guatemala | 0.02 (0.02, 0.03) | 151.75 (126.88, 180.27) | 133.90 (111.89, 159.26) |  | 0.02 (0.02, 0.03) | 34.72 (29.76, 40.74) | 28.78 (24.57, 33.80) |  | 0.01 (0.01, 0.01) | 14.76 (12.75, 17.17) | 11.32 (9.73, 13.21) |  | 0.31 (0.27, 0.37) | 472.16 (402.85, 552.62) | 385.89 (328.72, 452.60) |
| Guinea | 0.02 (0.01, 0.02) | 149.33 (101.40, 207.85) | 103.78 (69.29, 148.96) |  | 0.02 (0.01, 0.02) | 43.11 (30.96, 57.99) | 25.05 (17.41, 34.29) |  | 0.01 (0.01, 0.01) | 27.90 (20.26, 37.25) | 13.96 (9.98, 19.02) |  | 0.38 (0.26, 0.52) | 937.56 (664.26, 1285.66) | 543.00 (373.66, 750.81) |
| Guinea-Bissau | 0.00 (0.00, 0.00) | 152.74 (104.71, 200.90) | 108.19 (73.00, 142.51) |  | 0.00 (0.00, 0.00) | 46.88 (32.93, 60.31) | 26.72 (18.23, 35.08) |  | 0.00 (0.00, 0.00) | 31.89 (22.42, 40.63) | 15.26 (10.69, 19.85) |  | 0.06 (0.04, 0.08) | 1047.92 (733.50, 1363.28) | 603.13 (422.22, 801.02) |
| Guyana | 0.00 (0.00, 0.00) | 144.33 (101.31, 199.61) | 144.31 (101.22, 199.55) |  | 0.00 (0.00, 0.00) | 32.36 (23.52, 44.10) | 31.39 (22.58, 42.91) |  | 0.00 (0.00, 0.00) | 16.87 (12.78, 21.99) | 15.70 (11.82, 20.64) |  | 0.02 (0.02, 0.03) | 560.17 (414.70, 749.75) | 547.54 (402.80, 736.70) |
| Haiti | 0.02 (0.02, 0.03) | 168.39 (112.32, 237.78) | 154.85 (103.09, 218.35) |  | 0.02 (0.02, 0.03) | 45.94 (31.09, 64.15) | 36.67 (24.66, 51.46) |  | 0.01 (0.01, 0.02) | 28.28 (19.39, 39.02) | 19.88 (13.55, 27.72) |  | 0.50 (0.34, 0.70) | 948.31 (645.53, 1322.35) | 767.37 (513.98, 1061.95) |
| Honduras | 0.01 (0.01, 0.02) | 136.02 (81.17, 200.94) | 116.82 (68.61, 176.35) |  | 0.01 (0.01, 0.02) | 33.62 (20.76, 48.16) | 26.39 (15.73, 38.61) |  | 0.01 (0.00, 0.01) | 15.66 (10.06, 21.50) | 11.05 (7.08, 15.42) |  | 0.20 (0.12, 0.28) | 490.14 (297.52, 687.43) | 379.84 (222.70, 542.24) |
| Hungary | 0.01 (0.01, 0.01) | 97.94 (74.21, 123.50) | 126.51 (98.07, 158.45) |  | 0.01 (0.01, 0.01) | 15.75 (12.53, 19.59) | 23.13 (18.89, 28.38) |  | 0.00 (0.00, 0.01) | 4.73 (3.94, 5.63) | 8.96 (7.56, 10.54) |  | 0.13 (0.11, 0.15) | 157.29 (128.26, 190.65) | 252.37 (210.42, 301.61) |
| Iceland | 0.00 (0.00, 0.00) | 46.68 (39.13, 54.62) | 54.62 (46.59, 63.10) |  | 0.00 (0.00, 0.00) | 6.86 (5.87, 7.99) | 8.81 (7.67, 10.10) |  | 0.00 (0.00, 0.00) | 1.86 (1.61, 2.09) | 3.04 (2.58, 3.42) |  | 0.00 (0.00, 0.00) | 56.56 (49.57, 63.77) | 78.60 (69.00, 88.00) |
| India | 1.12 (0.96, 1.29) | 68.05 (58.11, 78.84) | 69.63 (59.51, 80.86) |  | 1.12 (0.96, 1.29) | 16.53 (14.14, 18.99) | 16.24 (13.87, 18.70) |  | 0.60 (0.52, 0.69) | 9.24 (7.96, 10.62) | 8.70 (7.47, 10.01) |  | 20.56 (17.58, 23.78) | 303.66 (260.09, 350.67) | 297.92 (254.68, 344.55) |
| Indonesia | 0.21 (0.15, 0.27) | 63.29 (46.17, 84.22) | 72.14 (52.53, 96.02) |  | 0.21 (0.15, 0.27) | 13.78 (10.07, 18.15) | 14.92 (10.85, 19.82) |  | 0.10 (0.07, 0.14) | 7.38 (5.39, 9.53) | 7.48 (5.41, 9.81) |  | 3.68 (2.66, 4.89) | 244.61 (176.99, 323.26) | 267.28 (192.79, 355.02) |
| Iran (Islamic Republic of) | 0.01 (0.01, 0.01) | 10.31 (8.65, 11.90) | 12.30 (10.33, 14.31) |  | 0.01 (0.01, 0.01) | 2.50 (2.16, 2.85) | 2.68 (2.31, 3.06) |  | 0.01 (0.00, 0.01) | 1.37 (1.18, 1.55) | 1.32 (1.14, 1.50) |  | 0.18 (0.15, 0.20) | 39.79 (34.54, 44.97) | 42.40 (36.90, 48.02) |
| Iraq | 0.01 (0.00, 0.01) | 14.34 (10.11, 20.27) | 12.68 (8.91, 17.95) |  | 0.01 (0.00, 0.01) | 3.51 (2.51, 4.80) | 2.73 (1.95, 3.79) |  | 0.00 (0.00, 0.00) | 1.86 (1.33, 2.48) | 1.28 (0.91, 1.72) |  | 0.09 (0.06, 0.12) | 58.40 (41.76, 78.99) | 44.90 (31.74, 61.33) |
| Ireland | 0.00 (0.00, 0.00) | 55.28 (47.67, 62.55) | 68.05 (59.03, 77.21) |  | 0.00 (0.00, 0.00) | 8.02 (7.04, 8.98) | 10.46 (9.18, 11.70) |  | 0.00 (0.00, 0.00) | 2.15 (1.90, 2.40) | 3.31 (2.88, 3.71) |  | 0.02 (0.02, 0.03) | 69.40 (61.55, 77.05) | 95.93 (85.59, 106.69) |
| Israel | 0.00 (0.00, 0.00) | 32.64 (28.15, 37.14) | 34.34 (29.70, 38.77) |  | 0.00 (0.00, 0.00) | 5.38 (4.76, 6.03) | 6.17 (5.46, 6.87) |  | 0.00 (0.00, 0.00) | 1.97 (1.73, 2.19) | 2.65 (2.28, 2.96) |  | 0.03 (0.03, 0.04) | 55.90 (50.10, 61.40) | 66.51 (59.05, 73.56) |
| Italy | 0.04 (0.04, 0.05) | 55.89 (51.71, 60.74) | 80.97 (73.83, 88.26) |  | 0.04 (0.04, 0.05) | 8.38 (7.79, 8.99) | 14.13 (12.76, 15.25) |  | 0.02 (0.01, 0.02) | 2.13 (1.92, 2.29) | 5.00 (4.32, 5.48) |  | 0.36 (0.33, 0.39) | 65.50 (60.87, 69.52) | 118.29 (107.03, 126.90) |
| Jamaica | 0.01 (0.00, 0.01) | 189.81 (136.73, 258.15) | 210.84 (152.07, 286.26) |  | 0.01 (0.00, 0.01) | 34.40 (25.19, 45.93) | 38.48 (28.30, 51.33) |  | 0.00 (0.00, 0.00) | 13.98 (10.53, 17.99) | 15.94 (12.02, 20.45) |  | 0.07 (0.05, 0.09) | 457.27 (339.87, 599.60) | 509.60 (378.85, 667.53) |
| Japan | 0.12 (0.11, 0.13) | 89.78 (83.79, 95.97) | 112.86 (104.45, 120.68) |  | 0.12 (0.11, 0.13) | 12.35 (11.56, 13.11) | 18.05 (16.40, 19.26) |  | 0.04 (0.03, 0.04) | 2.74 (2.52, 2.88) | 5.94 (5.02, 6.50) |  | 1.00 (0.91, 1.06) | 97.47 (92.46, 101.36) | 153.21 (139.12, 162.05) |
| Jordan | 0.00 (0.00, 0.00) | 12.93 (9.15, 18.19) | 12.09 (8.42, 17.00) |  | 0.00 (0.00, 0.00) | 2.95 (2.15, 3.96) | 2.38 (1.72, 3.26) |  | 0.00 (0.00, 0.00) | 1.47 (1.11, 1.90) | 1.00 (0.73, 1.32) |  | 0.02 (0.01, 0.03) | 43.66 (31.93, 58.31) | 34.58 (24.93, 47.24) |
| Kazakhstan | 0.02 (0.01, 0.02) | 93.23 (79.17, 108.40) | 103.08 (87.51, 120.06) |  | 0.02 (0.01, 0.02) | 16.20 (13.69, 18.77) | 17.93 (15.15, 20.75) |  | 0.01 (0.01, 0.01) | 5.88 (5.02, 6.85) | 6.48 (5.53, 7.56) |  | 0.23 (0.20, 0.27) | 211.67 (180.06, 245.42) | 235.55 (200.26, 273.39) |
| Kenya | 0.03 (0.02, 0.04) | 73.74 (51.96, 100.58) | 54.28 (38.33, 74.73) |  | 0.03 (0.02, 0.04) | 19.35 (13.61, 26.38) | 12.38 (8.71, 16.85) |  | 0.02 (0.01, 0.02) | 12.12 (8.57, 16.38) | 6.81 (4.82, 9.31) |  | 0.62 (0.44, 0.86) | 389.77 (275.36, 537.10) | 247.44 (174.62, 339.49) |
| Kiribati | 0.00 (0.00, 0.00) | 245.93 (172.63, 346.96) | 220.96 (155.10, 312.66) |  | 0.00 (0.00, 0.00) | 70.03 (50.27, 93.83) | 55.62 (39.44, 76.39) |  | 0.00 (0.00, 0.00) | 45.10 (32.22, 58.73) | 31.66 (22.64, 42.04) |  | 0.01 (0.00, 0.01) | 1402.05 (997.62, 1890.91) | 1137.01 (797.85, 1559.39) |
| Kuwait | 0.00 (0.00, 0.00) | 10.09 (8.33, 12.22) | 13.76 (11.23, 16.86) |  | 0.00 (0.00, 0.00) | 2.11 (1.78, 2.50) | 2.22 (1.85, 2.67) |  | 0.00 (0.00, 0.00) | 0.92 (0.77, 1.09) | 0.68 (0.57, 0.80) |  | 0.01 (0.00, 0.01) | 26.60 (22.44, 31.40) | 25.47 (21.38, 30.67) |
| Kyrgyzstan | 0.01 (0.00, 0.01) | 94.37 (71.59, 121.69) | 90.44 (68.42, 116.89) |  | 0.01 (0.00, 0.01) | 18.64 (14.65, 23.18) | 17.14 (13.34, 21.54) |  | 0.00 (0.00, 0.00) | 8.22 (6.70, 9.92) | 7.08 (5.75, 8.64) |  | 0.09 (0.07, 0.11) | 276.65 (221.69, 338.28) | 251.87 (199.51, 310.13) |
| Lao People's Democratic Republic | 0.00 (0.00, 0.01) | 61.89 (43.29, 82.97) | 56.84 (39.62, 76.73) |  | 0.00 (0.00, 0.01) | 14.98 (10.77, 19.32) | 12.55 (8.94, 16.34) |  | 0.00 (0.00, 0.00) | 8.71 (6.37, 11.47) | 6.65 (4.80, 8.83) |  | 0.09 (0.06, 0.12) | 293.78 (210.59, 392.09) | 246.88 (174.86, 330.08) |
| Latvia | 0.00 (0.00, 0.00) | 50.07 (40.43, 59.62) | 65.69 (53.91, 77.74) |  | 0.00 (0.00, 0.00) | 9.74 (8.11, 11.42) | 16.16 (13.58, 18.60) |  | 0.00 (0.00, 0.00) | 4.66 (3.99, 5.39) | 9.87 (8.38, 11.38) |  | 0.02 (0.02, 0.03) | 145.71 (122.57, 169.04) | 245.93 (209.84, 284.24) |
| Lebanon | 0.00 (0.00, 0.00) | 18.33 (13.64, 23.86) | 21.20 (15.69, 27.64) |  | 0.00 (0.00, 0.00) | 3.75 (2.86, 4.78) | 4.32 (3.30, 5.50) |  | 0.00 (0.00, 0.00) | 1.66 (1.29, 2.05) | 1.94 (1.51, 2.39) |  | 0.02 (0.01, 0.02) | 50.96 (39.09, 64.60) | 58.13 (44.62, 73.39) |
| Lesotho | 0.00 (0.00, 0.01) | 184.44 (104.15, 287.56) | 149.16 (83.69, 235.83) |  | 0.00 (0.00, 0.01) | 60.76 (36.14, 88.56) | 45.70 (26.70, 66.71) |  | 0.00 (0.00, 0.00) | 42.45 (25.97, 62.03) | 30.30 (18.27, 44.69) |  | 0.10 (0.06, 0.14) | 1337.99 (795.12, 1985.02) | 1011.14 (592.99, 1497.40) |
| Liberia | 0.01 (0.00, 0.01) | 123.81 (81.83, 173.82) | 91.24 (59.26, 129.30) |  | 0.01 (0.00, 0.01) | 33.77 (23.34, 46.43) | 20.01 (13.27, 27.78) |  | 0.00 (0.00, 0.00) | 21.18 (14.71, 29.17) | 10.20 (7.05, 14.22) |  | 0.11 (0.07, 0.15) | 672.26 (461.97, 937.48) | 393.89 (264.47, 549.37) |
| Libya | 0.00 (0.00, 0.01) | 55.16 (37.90, 77.96) | 66.35 (45.28, 94.77) |  | 0.00 (0.00, 0.01) | 12.19 (8.71, 16.81) | 12.97 (9.10, 17.90) |  | 0.00 (0.00, 0.00) | 6.07 (4.43, 8.19) | 5.66 (4.06, 7.67) |  | 0.07 (0.05, 0.10) | 200.21 (143.37, 271.20) | 210.69 (148.45, 287.37) |
| Lithuania | 0.00 (0.00, 0.00) | 99.78 (83.67, 117.78) | 130.52 (109.86, 152.98) |  | 0.00 (0.00, 0.00) | 16.75 (14.30, 19.55) | 25.96 (22.26, 29.99) |  | 0.00 (0.00, 0.00) | 5.72 (4.87, 6.55) | 11.94 (10.22, 13.63) |  | 0.04 (0.04, 0.05) | 181.07 (151.40, 208.23) | 304.69 (260.12, 348.62) |
| Luxembourg | 0.00 (0.00, 0.00) | 31.88 (28.06, 36.15) | 42.16 (37.08, 47.69) |  | 0.00 (0.00, 0.00) | 4.99 (4.43, 5.57) | 7.18 (6.37, 8.00) |  | 0.00 (0.00, 0.00) | 1.64 (1.47, 1.82) | 2.85 (2.53, 3.17) |  | 0.00 (0.00, 0.00) | 47.76 (42.71, 53.04) | 72.28 (64.60, 80.32) |
| Madagascar | 0.03 (0.02, 0.05) | 131.64 (81.63, 188.94) | 95.57 (59.37, 137.02) |  | 0.03 (0.02, 0.05) | 36.33 (22.19, 51.65) | 22.52 (13.84, 32.21) |  | 0.02 (0.01, 0.02) | 22.97 (14.17, 31.64) | 12.34 (7.66, 17.14) |  | 0.71 (0.44, 1.00) | 786.27 (486.92, 1092.78) | 489.96 (302.68, 692.46) |
| Malawi | 0.03 (0.02, 0.04) | 167.91 (109.56, 240.18) | 117.02 (74.37, 171.47) |  | 0.03 (0.02, 0.04) | 44.36 (29.18, 61.73) | 26.21 (17.12, 37.08) |  | 0.01 (0.01, 0.02) | 27.26 (18.29, 37.60) | 13.80 (9.07, 19.30) |  | 0.54 (0.35, 0.77) | 931.99 (610.09, 1301.94) | 544.49 (352.70, 776.69) |
| Malaysia | 0.03 (0.02, 0.04) | 99.25 (74.51, 117.46) | 103.61 (77.97, 122.94) |  | 0.03 (0.02, 0.04) | 19.44 (15.08, 22.81) | 19.51 (15.12, 22.97) |  | 0.01 (0.01, 0.02) | 9.45 (7.27, 11.02) | 8.90 (6.89, 10.39) |  | 0.43 (0.33, 0.51) | 282.80 (216.70, 330.76) | 283.01 (216.66, 329.28) |
| Maldives | 0.00 (0.00, 0.00) | 32.76 (24.65, 42.12) | 35.52 (26.44, 45.32) |  | 0.00 (0.00, 0.00) | 5.98 (4.56, 7.53) | 5.86 (4.46, 7.36) |  | 0.00 (0.00, 0.00) | 2.68 (2.06, 3.45) | 2.22 (1.68, 2.83) |  | 0.00 (0.00, 0.00) | 79.42 (60.19, 100.55) | 75.25 (56.23, 95.18) |
| Mali | 0.02 (0.01, 0.02) | 102.39 (73.15, 139.44) | 64.26 (45.22, 88.31) |  | 0.02 (0.01, 0.02) | 28.29 (21.21, 37.38) | 14.80 (10.76, 19.89) |  | 0.01 (0.01, 0.01) | 17.81 (13.31, 23.35) | 7.91 (5.96, 10.36) |  | 0.38 (0.28, 0.50) | 595.51 (445.53, 785.05) | 309.63 (228.63, 415.54) |
| Malta | 0.00 (0.00, 0.00) | 36.63 (31.17, 42.65) | 46.93 (40.11, 54.01) |  | 0.00 (0.00, 0.00) | 5.62 (4.87, 6.40) | 8.46 (7.35, 9.56) |  | 0.00 (0.00, 0.00) | 1.74 (1.52, 1.98) | 3.58 (3.09, 4.13) |  | 0.00 (0.00, 0.00) | 53.73 (46.70, 61.64) | 88.55 (76.78, 100.58) |
| Marshall Islands | 0.00 (0.00, 0.00) | 144.07 (87.09, 201.52) | 140.10 (84.09, 197.78) |  | 0.00 (0.00, 0.00) | 34.03 (21.47, 46.90) | 29.83 (18.48, 41.92) |  | 0.00 (0.00, 0.00) | 18.28 (12.03, 24.61) | 14.02 (8.79, 19.23) |  | 0.00 (0.00, 0.00) | 626.75 (392.07, 861.80) | 555.17 (342.03, 775.86) |
| Mauritania | 0.00 (0.00, 0.01) | 115.57 (79.62, 164.96) | 79.70 (54.15, 116.67) |  | 0.00 (0.00, 0.01) | 30.36 (21.35, 41.47) | 18.31 (12.91, 25.27) |  | 0.00 (0.00, 0.00) | 18.53 (13.06, 24.62) | 9.85 (6.89, 13.31) |  | 0.07 (0.05, 0.10) | 560.80 (389.98, 760.04) | 334.92 (234.05, 460.59) |
| Mauritius | 0.00 (0.00, 0.00) | 73.52 (64.78, 80.93) | 94.77 (83.57, 104.23) |  | 0.00 (0.00, 0.00) | 13.38 (11.97, 14.37) | 18.29 (16.39, 19.63) |  | 0.00 (0.00, 0.00) | 5.89 (5.30, 6.25) | 8.69 (7.81, 9.21) |  | 0.02 (0.02, 0.02) | 188.41 (168.14, 200.79) | 263.91 (235.94, 281.31) |
| Mexico | 0.16 (0.14, 0.19) | 118.91 (98.40, 139.54) | 130.46 (107.92, 153.23) |  | 0.16 (0.14, 0.19) | 22.99 (19.14, 26.86) | 24.88 (20.70, 29.12) |  | 0.05 (0.05, 0.06) | 7.87 (6.64, 9.12) | 8.27 (6.96, 9.59) |  | 1.83 (1.53, 2.14) | 255.74 (213.83, 297.88) | 277.19 (231.33, 323.33) |
| Micronesia (Federated States of) | 0.00 (0.00, 0.00) | 132.77 (90.87, 189.19) | 125.56 (85.60, 179.59) |  | 0.00 (0.00, 0.00) | 31.05 (22.09, 43.65) | 27.78 (19.48, 39.22) |  | 0.00 (0.00, 0.00) | 16.41 (12.00, 22.27) | 13.53 (9.66, 18.58) |  | 0.00 (0.00, 0.00) | 543.60 (384.04, 747.25) | 492.09 (343.91, 681.27) |
| Monaco | 0.00 (0.00, 0.00) | 74.96 (51.00, 106.22) | 87.70 (61.35, 120.63) |  | 0.00 (0.00, 0.00) | 10.93 (7.63, 15.05) | 15.70 (11.37, 20.96) |  | 0.00 (0.00, 0.00) | 2.89 (2.10, 3.83) | 6.61 (4.88, 8.78) |  | 0.00 (0.00, 0.00) | 93.32 (66.33, 125.49) | 157.52 (115.40, 209.31) |
| Mongolia | 0.00 (0.00, 0.00) | 79.12 (58.55, 103.35) | 82.82 (61.63, 107.90) |  | 0.00 (0.00, 0.00) | 18.35 (13.61, 24.61) | 17.61 (13.14, 23.29) |  | 0.00 (0.00, 0.00) | 9.64 (7.14, 12.68) | 8.27 (6.13, 10.91) |  | 0.05 (0.04, 0.06) | 303.39 (224.72, 400.71) | 289.60 (215.37, 383.25) |
| Montenegro | 0.00 (0.00, 0.00) | 89.82 (68.56, 115.26) | 111.71 (85.89, 141.70) |  | 0.00 (0.00, 0.00) | 14.50 (11.30, 18.41) | 19.57 (15.30, 24.60) |  | 0.00 (0.00, 0.00) | 4.50 (3.51, 5.58) | 7.11 (5.55, 8.85) |  | 0.01 (0.01, 0.01) | 142.21 (112.10, 178.28) | 205.94 (161.11, 255.64) |
| Morocco | 0.02 (0.01, 0.03) | 40.23 (27.32, 58.29) | 43.55 (29.42, 63.27) |  | 0.02 (0.01, 0.03) | 10.26 (6.93, 13.91) | 10.76 (7.19, 14.73) |  | 0.01 (0.01, 0.01) | 5.79 (4.00, 7.66) | 5.87 (3.97, 7.86) |  | 0.37 (0.24, 0.52) | 192.10 (126.36, 262.58) | 202.43 (131.95, 279.30) |
| Mozambique | 0.04 (0.02, 0.07) | 156.39 (94.42, 261.36) | 98.10 (59.29, 169.18) |  | 0.04 (0.02, 0.07) | 46.72 (28.79, 74.22) | 24.94 (15.15, 40.75) |  | 0.02 (0.01, 0.04) | 31.27 (19.31, 50.23) | 14.65 (8.91, 23.62) |  | 0.90 (0.54, 1.45) | 1038.62 (631.61, 1686.00) | 559.06 (337.36, 897.90) |
| Myanmar | 0.04 (0.03, 0.05) | 59.78 (42.79, 83.42) | 62.84 (45.03, 87.83) |  | 0.04 (0.03, 0.05) | 13.07 (9.29, 17.92) | 13.48 (9.59, 18.54) |  | 0.02 (0.01, 0.03) | 7.04 (5.09, 9.97) | 7.07 (5.11, 10.00) |  | 0.73 (0.52, 1.01) | 239.18 (170.86, 332.57) | 248.61 (177.69, 345.70) |
| Namibia | 0.00 (0.00, 0.00) | 104.82 (65.71, 159.61) | 90.71 (55.71, 139.90) |  | 0.00 (0.00, 0.00) | 26.70 (17.11, 37.91) | 20.79 (13.00, 29.86) |  | 0.00 (0.00, 0.00) | 15.86 (10.56, 21.45) | 11.13 (7.23, 15.29) |  | 0.05 (0.03, 0.07) | 502.55 (320.98, 703.54) | 388.57 (246.42, 555.20) |
| Nauru | 0.00 (0.00, 0.00) | 189.56 (113.00, 273.30) | 162.08 (95.64, 234.17) |  | 0.00 (0.00, 0.00) | 42.10 (26.19, 58.95) | 32.56 (19.77, 46.48) |  | 0.00 (0.00, 0.00) | 21.26 (13.73, 28.90) | 14.40 (9.05, 20.07) |  | 0.00 (0.00, 0.00) | 726.73 (454.08, 1027.11) | 557.81 (338.83, 802.87) |
| Nepal | 0.02 (0.02, 0.03) | 65.68 (43.52, 90.38) | 62.21 (40.58, 87.25) |  | 0.02 (0.02, 0.03) | 15.96 (10.93, 21.11) | 14.08 (9.41, 18.80) |  | 0.01 (0.01, 0.02) | 9.18 (6.42, 12.27) | 7.51 (5.17, 10.05) |  | 0.43 (0.29, 0.58) | 301.00 (204.31, 401.87) | 264.16 (178.19, 354.47) |
| Netherlands | 0.01 (0.01, 0.01) | 51.56 (45.35, 58.78) | 66.31 (58.85, 75.06) |  | 0.01 (0.01, 0.01) | 7.26 (6.42, 8.20) | 10.37 (9.24, 11.60) |  | 0.00 (0.00, 0.00) | 1.85 (1.67, 2.07) | 3.59 (3.21, 4.02) |  | 0.08 (0.07, 0.09) | 57.85 (51.27, 64.81) | 90.49 (81.00, 100.81) |
| New Zealand | 0.00 (0.00, 0.00) | 35.01 (30.92, 39.89) | 41.30 (36.74, 46.69) |  | 0.00 (0.00, 0.00) | 5.40 (4.81, 6.09) | 6.87 (6.22, 7.67) |  | 0.00 (0.00, 0.00) | 1.62 (1.47, 1.78) | 2.42 (2.15, 2.66) |  | 0.02 (0.02, 0.02) | 55.68 (50.10, 61.91) | 72.37 (65.48, 79.96) |
| Nicaragua | 0.01 (0.01, 0.01) | 170.71 (130.53, 221.68) | 165.87 (126.21, 216.51) |  | 0.01 (0.01, 0.01) | 33.30 (25.96, 42.94) | 30.67 (23.79, 39.69) |  | 0.00 (0.00, 0.00) | 11.89 (9.55, 15.30) | 10.06 (8.00, 13.02) |  | 0.12 (0.09, 0.15) | 382.68 (299.52, 494.20) | 345.84 (268.29, 445.88) |
| Niger | 0.02 (0.01, 0.02) | 92.01 (63.81, 133.94) | 49.90 (33.94, 73.54) |  | 0.02 (0.01, 0.02) | 29.47 (20.91, 41.45) | 13.24 (9.18, 19.02) |  | 0.01 (0.01, 0.01) | 20.73 (15.04, 28.90) | 8.08 (5.73, 11.43) |  | 0.37 (0.26, 0.53) | 641.32 (454.74, 905.74) | 292.74 (203.02, 420.91) |
| Nigeria | 0.13 (0.08, 0.19) | 61.43 (36.95, 91.97) | 42.66 (24.87, 65.96) |  | 0.13 (0.08, 0.19) | 18.50 (11.98, 26.63) | 10.73 (6.67, 15.94) |  | 0.07 (0.05, 0.10) | 11.83 (8.10, 16.36) | 5.82 (3.81, 8.29) |  | 2.61 (1.63, 3.83) | 372.29 (241.58, 531.52) | 216.62 (135.17, 317.50) |
| Niue | 0.00 (0.00, 0.00) | 125.32 (95.68, 161.00) | 138.39 (104.82, 177.92) |  | 0.00 (0.00, 0.00) | 24.23 (18.65, 30.41) | 29.00 (22.12, 36.67) |  | 0.00 (0.00, 0.00) | 10.52 (8.08, 13.29) | 13.91 (10.55, 17.67) |  | 0.00 (0.00, 0.00) | 339.60 (263.20, 431.84) | 417.41 (316.44, 530.05) |
| North Macedonia | 0.00 (0.00, 0.00) | 82.42 (59.85, 107.82) | 110.95 (80.24, 145.21) |  | 0.00 (0.00, 0.00) | 14.86 (10.96, 19.46) | 21.11 (15.53, 27.79) |  | 0.00 (0.00, 0.00) | 5.64 (4.20, 7.33) | 8.57 (6.34, 11.19) |  | 0.03 (0.02, 0.04) | 171.73 (127.49, 224.92) | 254.30 (186.33, 334.55) |
| Northern Mariana Islands | 0.00 (0.00, 0.00) | 186.78 (144.43, 229.02) | 229.17 (176.03, 283.33) |  | 0.00 (0.00, 0.00) | 35.36 (28.14, 42.42) | 42.16 (32.85, 51.11) |  | 0.00 (0.00, 0.00) | 15.03 (12.08, 17.40) | 16.72 (13.29, 19.79) |  | 0.00 (0.00, 0.00) | 460.48 (364.45, 546.50) | 564.88 (445.31, 678.85) |
| Norway | 0.00 (0.00, 0.00) | 59.14 (54.17, 64.09) | 70.12 (64.25, 75.97) |  | 0.00 (0.00, 0.00) | 9.03 (8.34, 9.72) | 12.33 (11.35, 13.25) |  | 0.00 (0.00, 0.00) | 2.86 (2.60, 3.07) | 5.37 (4.70, 5.83) |  | 0.03 (0.03, 0.04) | 84.11 (78.44, 89.54) | 127.62 (117.44, 136.52) |
| Oman | 0.00 (0.00, 0.00) | 14.80 (10.82, 19.90) | 14.66 (10.49, 20.03) |  | 0.00 (0.00, 0.00) | 3.37 (2.53, 4.27) | 2.59 (1.90, 3.44) |  | 0.00 (0.00, 0.00) | 1.62 (1.23, 2.08) | 0.93 (0.70, 1.20) |  | 0.01 (0.00, 0.01) | 48.50 (36.66, 61.83) | 34.22 (25.19, 45.05) |
| Pakistan | 0.07 (0.05, 0.09) | 28.28 (19.08, 39.62) | 24.74 (16.62, 35.07) |  | 0.07 (0.05, 0.09) | 7.87 (5.61, 10.71) | 5.86 (4.11, 8.10) |  | 0.03 (0.02, 0.05) | 4.66 (3.34, 6.25) | 2.94 (2.10, 3.98) |  | 1.34 (0.93, 1.82) | 157.49 (111.57, 213.14) | 116.23 (81.15, 158.60) |
| Palau | 0.00 (0.00, 0.00) | 263.73 (192.73, 354.95) | 337.77 (247.94, 453.70) |  | 0.00 (0.00, 0.00) | 57.48 (44.17, 74.16) | 71.98 (55.02, 93.59) |  | 0.00 (0.00, 0.00) | 30.03 (23.64, 37.61) | 35.05 (27.65, 44.68) |  | 0.00 (0.00, 0.00) | 819.64 (635.71, 1054.51) | 1072.97 (820.58, 1397.13) |
| Palestine | 0.00 (0.00, 0.00) | 6.28 (4.83, 7.98) | 4.93 (3.71, 6.31) |  | 0.00 (0.00, 0.00) | 1.67 (1.32, 2.07) | 1.09 (0.85, 1.36) |  | 0.00 (0.00, 0.00) | 0.97 (0.78, 1.17) | 0.54 (0.43, 0.66) |  | 0.00 (0.00, 0.01) | 27.49 (21.71, 33.98) | 17.56 (13.64, 22.06) |
| Panama | 0.01 (0.01, 0.01) | 184.19 (143.68, 228.37) | 188.63 (147.26, 233.66) |  | 0.01 (0.01, 0.01) | 31.31 (24.85, 38.21) | 32.43 (25.78, 39.52) |  | 0.00 (0.00, 0.00) | 8.44 (6.68, 10.24) | 9.00 (7.13, 10.91) |  | 0.06 (0.05, 0.08) | 289.07 (227.62, 350.05) | 301.51 (237.32, 365.48) |
| Papua New Guinea | 0.01 (0.01, 0.01) | 95.35 (67.76, 164.81) | 79.56 (56.15, 136.87) |  | 0.01 (0.01, 0.01) | 24.52 (17.62, 41.84) | 17.48 (12.52, 29.77) |  | 0.00 (0.00, 0.01) | 14.00 (9.87, 23.93) | 8.41 (5.93, 14.53) |  | 0.17 (0.12, 0.29) | 462.31 (326.46, 798.92) | 330.80 (233.46, 574.80) |
| Paraguay | 0.01 (0.01, 0.02) | 180.35 (129.49, 251.34) | 177.63 (127.67, 249.28) |  | 0.01 (0.01, 0.02) | 35.59 (26.12, 48.68) | 33.72 (24.63, 46.19) |  | 0.01 (0.00, 0.01) | 16.04 (11.94, 21.38) | 14.39 (10.69, 19.22) |  | 0.17 (0.13, 0.23) | 519.03 (384.24, 693.92) | 484.60 (358.91, 650.99) |
| Peru | 0.06 (0.04, 0.08) | 189.68 (135.66, 251.42) | 197.39 (141.25, 262.92) |  | 0.06 (0.04, 0.08) | 30.77 (21.95, 40.85) | 31.60 (22.53, 41.92) |  | 0.02 (0.02, 0.03) | 13.17 (9.51, 17.37) | 13.10 (9.42, 17.23) |  | 0.75 (0.54, 0.99) | 409.73 (294.37, 541.09) | 416.78 (298.62, 550.24) |
| Philippines | 0.07 (0.05, 0.09) | 57.19 (42.23, 75.03) | 54.72 (40.35, 72.05) |  | 0.07 (0.05, 0.09) | 12.94 (9.80, 16.55) | 11.89 (8.93, 15.26) |  | 0.03 (0.02, 0.04) | 6.47 (5.05, 8.11) | 5.64 (4.36, 7.11) |  | 1.16 (0.89, 1.48) | 226.50 (173.15, 288.67) | 207.89 (158.53, 265.81) |
| Poland | 0.03 (0.03, 0.04) | 48.93 (42.54, 55.45) | 66.24 (57.59, 75.50) |  | 0.03 (0.03, 0.04) | 10.63 (9.34, 11.88) | 16.92 (14.98, 18.86) |  | 0.02 (0.02, 0.02) | 5.17 (4.58, 5.72) | 9.92 (8.79, 10.96) |  | 0.50 (0.44, 0.56) | 152.82 (134.74, 169.80) | 254.13 (224.45, 281.73) |
| Portugal | 0.01 (0.01, 0.01) | 81.32 (72.18, 91.26) | 111.25 (98.66, 123.22) |  | 0.01 (0.01, 0.01) | 11.73 (10.56, 13.02) | 18.41 (16.41, 20.29) |  | 0.00 (0.00, 0.00) | 2.95 (2.64, 3.23) | 6.44 (5.63, 7.20) |  | 0.09 (0.08, 0.10) | 95.13 (86.07, 104.54) | 159.04 (142.99, 174.27) |
| Puerto Rico | 0.00 (0.00, 0.00) | 77.52 (61.22, 94.99) | 97.98 (78.29, 118.94) |  | 0.00 (0.00, 0.00) | 11.83 (9.37, 14.35) | 17.11 (13.74, 20.55) |  | 0.00 (0.00, 0.00) | 3.44 (2.81, 4.14) | 6.58 (5.39, 7.94) |  | 0.03 (0.02, 0.04) | 114.84 (92.28, 138.78) | 176.28 (144.09, 212.26) |
| Qatar | 0.00 (0.00, 0.00) | 15.01 (10.91, 20.79) | 15.59 (11.03, 21.64) |  | 0.00 (0.00, 0.00) | 3.95 (2.94, 5.29) | 2.60 (1.88, 3.63) |  | 0.00 (0.00, 0.00) | 2.04 (1.55, 2.69) | 0.80 (0.59, 1.13) |  | 0.00 (0.00, 0.00) | 52.50 (39.22, 71.41) | 29.79 (21.48, 42.63) |
| Republic of Korea | 0.03 (0.03, 0.04) | 61.26 (48.64, 79.26) | 84.46 (66.57, 110.07) |  | 0.03 (0.03, 0.04) | 8.88 (7.12, 11.47) | 13.32 (10.48, 17.37) |  | 0.01 (0.01, 0.01) | 2.25 (1.77, 2.89) | 4.18 (3.24, 5.40) |  | 0.29 (0.23, 0.38) | 70.79 (56.61, 91.26) | 113.87 (89.66, 149.30) |
| Republic of Moldova | 0.00 (0.00, 0.00) | 86.85 (75.01, 99.65) | 117.86 (101.82, 135.13) |  | 0.00 (0.00, 0.00) | 15.28 (13.36, 17.56) | 22.51 (19.80, 25.87) |  | 0.00 (0.00, 0.00) | 6.49 (5.79, 7.29) | 10.74 (9.62, 12.07) |  | 0.06 (0.06, 0.07) | 223.41 (197.65, 252.55) | 338.34 (301.18, 382.53) |
| Romania | 0.04 (0.03, 0.05) | 161.82 (135.94, 188.39) | 210.87 (178.76, 246.04) |  | 0.04 (0.03, 0.05) | 27.86 (23.77, 32.03) | 41.34 (35.48, 47.24) |  | 0.02 (0.02, 0.02) | 10.45 (9.14, 11.90) | 18.92 (16.65, 21.36) |  | 0.53 (0.46, 0.60) | 347.84 (302.45, 397.40) | 547.52 (476.71, 621.23) |
| Russian Federation | 0.20 (0.18, 0.22) | 125.65 (110.04, 140.28) | 156.22 (137.07, 174.28) |  | 0.20 (0.18, 0.22) | 19.39 (17.08, 21.57) | 26.21 (23.18, 29.06) |  | 0.07 (0.07, 0.08) | 6.14 (5.44, 6.80) | 9.68 (8.60, 10.65) |  | 2.49 (2.20, 2.76) | 229.21 (201.84, 255.11) | 321.35 (283.88, 356.92) |
| Rwanda | 0.02 (0.01, 0.02) | 121.75 (78.51, 177.86) | 96.37 (62.05, 139.16) |  | 0.02 (0.01, 0.02) | 33.79 (22.00, 49.27) | 23.50 (15.13, 34.32) |  | 0.01 (0.01, 0.01) | 21.41 (14.07, 31.61) | 13.27 (8.59, 19.65) |  | 0.33 (0.21, 0.49) | 698.98 (453.07, 1040.69) | 485.82 (312.39, 716.63) |
| Saint Kitts and Nevis | 0.00 (0.00, 0.00) | 126.38 (99.08, 160.93) | 165.93 (130.80, 210.73) |  | 0.00 (0.00, 0.00) | 26.51 (21.75, 32.82) | 34.24 (27.87, 42.61) |  | 0.00 (0.00, 0.00) | 13.12 (10.93, 15.82) | 16.42 (13.57, 19.93) |  | 0.00 (0.00, 0.00) | 379.43 (309.03, 465.11) | 497.80 (405.10, 613.42) |
| Saint Lucia | 0.00 (0.00, 0.00) | 157.96 (125.67, 194.84) | 199.81 (159.37, 247.10) |  | 0.00 (0.00, 0.00) | 29.38 (23.48, 35.98) | 38.67 (30.95, 47.20) |  | 0.00 (0.00, 0.00) | 12.50 (10.06, 14.99) | 17.42 (14.01, 20.89) |  | 0.00 (0.00, 0.01) | 402.90 (319.27, 488.97) | 539.59 (429.35, 654.31) |
| Saint Vincent and the Grenadines | 0.00 (0.00, 0.00) | 221.89 (185.90, 264.05) | 252.51 (211.71, 301.78) |  | 0.00 (0.00, 0.00) | 42.23 (36.11, 49.24) | 49.52 (42.24, 57.67) |  | 0.00 (0.00, 0.00) | 18.36 (15.92, 21.25) | 22.31 (19.33, 25.80) |  | 0.00 (0.00, 0.00) | 624.32 (537.63, 728.86) | 741.41 (636.63, 865.94) |
| Samoa | 0.00 (0.00, 0.00) | 138.27 (90.72, 194.87) | 113.68 (74.84, 160.51) |  | 0.00 (0.00, 0.00) | 26.91 (18.14, 36.65) | 21.37 (14.38, 29.07) |  | 0.00 (0.00, 0.00) | 11.75 (8.15, 15.75) | 8.83 (6.08, 11.84) |  | 0.00 (0.00, 0.00) | 415.07 (280.36, 569.63) | 327.33 (221.53, 450.76) |
| San Marino | 0.00 (0.00, 0.00) | 20.75 (11.16, 33.55) | 26.90 (15.18, 43.67) |  | 0.00 (0.00, 0.00) | 3.09 (1.74, 4.97) | 4.66 (2.76, 7.35) |  | 0.00 (0.00, 0.00) | 0.89 (0.55, 1.39) | 1.91 (1.19, 2.86) |  | 0.00 (0.00, 0.00) | 27.53 (16.41, 44.23) | 46.64 (28.64, 73.69) |
| Sao Tome and Principe | 0.00 (0.00, 0.00) | 170.17 (116.11, 242.97) | 137.81 (92.02, 196.95) |  | 0.00 (0.00, 0.00) | 40.02 (27.59, 54.17) | 28.57 (19.38, 39.74) |  | 0.00 (0.00, 0.00) | 22.81 (16.04, 30.13) | 14.21 (9.98, 18.97) |  | 0.01 (0.00, 0.01) | 729.59 (509.29, 978.34) | 514.28 (350.72, 698.85) |
| Saudi Arabia | 0.01 (0.00, 0.01) | 18.25 (13.00, 25.41) | 23.41 (16.35, 33.39) |  | 0.01 (0.00, 0.01) | 3.56 (2.60, 4.90) | 3.86 (2.74, 5.45) |  | 0.00 (0.00, 0.00) | 0.89 (0.66, 1.17) | 0.66 (0.48, 0.91) |  | 0.04 (0.03, 0.06) | 29.96 (21.95, 40.49) | 28.18 (20.18, 39.06) |
| Senegal | 0.02 (0.01, 0.02) | 111.21 (79.65, 151.91) | 82.41 (58.51, 113.35) |  | 0.02 (0.01, 0.02) | 30.28 (21.81, 40.80) | 19.50 (13.95, 26.28) |  | 0.01 (0.01, 0.01) | 19.51 (14.12, 26.28) | 11.12 (8.01, 15.03) |  | 0.31 (0.22, 0.42) | 605.85 (434.01, 818.57) | 389.41 (279.19, 527.86) |
| Serbia | 0.01 (0.01, 0.02) | 127.38 (96.61, 165.11) | 166.43 (127.44, 213.75) |  | 0.01 (0.01, 0.02) | 21.29 (16.31, 27.21) | 31.15 (24.25, 39.53) |  | 0.01 (0.00, 0.01) | 7.54 (5.88, 9.56) | 13.33 (10.43, 16.92) |  | 0.17 (0.13, 0.21) | 242.93 (184.82, 310.70) | 376.41 (290.43, 480.93) |
| Seychelles | 0.00 (0.00, 0.00) | 157.27 (127.02, 189.49) | 185.19 (150.23, 223.73) |  | 0.00 (0.00, 0.00) | 29.05 (24.10, 34.39) | 34.64 (28.69, 41.08) |  | 0.00 (0.00, 0.00) | 13.13 (10.87, 15.46) | 15.90 (13.23, 18.71) |  | 0.00 (0.00, 0.00) | 423.92 (351.36, 498.42) | 514.09 (425.91, 605.10) |
| Sierra Leone | 0.01 (0.01, 0.01) | 111.91 (77.15, 153.11) | 78.68 (53.82, 107.81) |  | 0.01 (0.01, 0.01) | 31.89 (23.18, 42.35) | 18.48 (13.25, 25.02) |  | 0.00 (0.00, 0.01) | 20.84 (15.55, 28.11) | 10.27 (7.45, 13.93) |  | 0.17 (0.12, 0.23) | 662.33 (475.34, 906.34) | 380.75 (265.48, 525.15) |
| Singapore | 0.00 (0.00, 0.00) | 47.35 (42.38, 53.03) | 67.66 (60.35, 75.80) |  | 0.00 (0.00, 0.00) | 7.19 (6.50, 7.95) | 10.50 (9.45, 11.60) |  | 0.00 (0.00, 0.00) | 2.13 (1.90, 2.34) | 3.28 (2.92, 3.60) |  | 0.03 (0.02, 0.03) | 63.57 (57.10, 70.29) | 94.24 (84.73, 104.17) |
| Slovakia | 0.01 (0.00, 0.01) | 89.38 (66.11, 113.82) | 117.93 (87.09, 151.88) |  | 0.01 (0.00, 0.01) | 14.47 (10.90, 18.48) | 21.01 (15.69, 26.88) |  | 0.00 (0.00, 0.00) | 4.83 (3.61, 6.24) | 8.33 (6.19, 10.87) |  | 0.07 (0.05, 0.09) | 159.61 (120.04, 202.86) | 244.00 (182.33, 313.69) |
| Slovenia | 0.00 (0.00, 0.00) | 144.91 (112.43, 176.84) | 182.28 (145.67, 220.47) |  | 0.00 (0.00, 0.00) | 20.02 (15.90, 24.25) | 27.74 (22.39, 33.20) |  | 0.00 (0.00, 0.00) | 2.38 (1.95, 2.80) | 5.06 (4.17, 5.97) |  | 0.01 (0.01, 0.02) | 78.47 (62.56, 94.59) | 132.36 (107.86, 158.54) |
| Solomon Islands | 0.00 (0.00, 0.00) | 137.84 (97.07, 197.20) | 113.92 (79.00, 164.35) |  | 0.00 (0.00, 0.00) | 32.69 (23.81, 45.97) | 23.87 (17.16, 34.52) |  | 0.00 (0.00, 0.00) | 18.40 (13.68, 25.39) | 11.68 (8.43, 16.70) |  | 0.02 (0.01, 0.02) | 622.82 (451.14, 889.28) | 458.34 (325.44, 666.65) |
| Somalia | 0.03 (0.02, 0.04) | 147.64 (98.70, 224.88) | 85.88 (56.06, 132.70) |  | 0.03 (0.02, 0.04) | 50.52 (34.42, 73.38) | 24.45 (16.48, 36.59) |  | 0.02 (0.01, 0.02) | 36.56 (25.22, 51.80) | 15.60 (10.63, 22.55) |  | 0.64 (0.43, 0.95) | 1220.34 (838.53, 1777.02) | 603.56 (403.84, 899.68) |
| South Africa | 0.12 (0.10, 0.14) | 171.53 (147.94, 197.57) | 184.47 (158.48, 213.18) |  | 0.12 (0.10, 0.14) | 39.99 (34.95, 45.56) | 41.03 (35.81, 46.76) |  | 0.06 (0.05, 0.07) | 21.26 (18.75, 24.11) | 20.57 (18.13, 23.32) |  | 2.08 (1.82, 2.37) | 703.46 (616.54, 798.67) | 718.01 (627.39, 816.20) |
| South Sudan | 0.01 (0.01, 0.02) | 147.19 (97.18, 215.50) | 102.54 (66.81, 150.35) |  | 0.01 (0.01, 0.02) | 43.28 (29.08, 62.75) | 25.52 (17.13, 37.31) |  | 0.01 (0.00, 0.01) | 28.14 (19.33, 40.75) | 14.39 (9.66, 20.95) |  | 0.27 (0.18, 0.39) | 938.70 (629.96, 1371.88) | 559.98 (379.11, 817.35) |
| Spain | 0.03 (0.03, 0.04) | 61.70 (55.01, 68.87) | 87.53 (78.04, 97.47) |  | 0.03 (0.03, 0.04) | 8.67 (7.77, 9.64) | 13.52 (12.05, 15.06) |  | 0.01 (0.01, 0.01) | 1.92 (1.72, 2.12) | 3.96 (3.44, 4.42) |  | 0.24 (0.22, 0.27) | 62.73 (56.57, 69.36) | 103.82 (93.15, 115.04) |
| Sri Lanka | 0.01 (0.01, 0.02) | 43.44 (26.13, 61.25) | 52.22 (31.72, 74.31) |  | 0.01 (0.01, 0.02) | 7.98 (5.04, 11.14) | 9.91 (6.22, 13.96) |  | 0.01 (0.00, 0.01) | 3.53 (2.30, 4.91) | 4.56 (2.97, 6.38) |  | 0.15 (0.10, 0.22) | 105.74 (66.07, 149.40) | 133.57 (83.36, 189.33) |
| Sudan | 0.01 (0.00, 0.01) | 17.25 (9.93, 26.11) | 14.06 (7.79, 22.08) |  | 0.01 (0.00, 0.01) | 4.59 (2.85, 6.78) | 3.02 (1.76, 4.60) |  | 0.00 (0.00, 0.00) | 2.71 (1.81, 3.78) | 1.47 (0.90, 2.13) |  | 0.12 (0.07, 0.18) | 89.19 (54.78, 129.43) | 57.61 (33.59, 85.83) |
| Suriname | 0.00 (0.00, 0.00) | 159.14 (111.87, 211.14) | 175.62 (123.65, 232.50) |  | 0.00 (0.00, 0.00) | 31.93 (23.32, 41.19) | 36.02 (26.25, 46.37) |  | 0.00 (0.00, 0.00) | 14.87 (10.95, 18.85) | 17.16 (12.66, 21.79) |  | 0.02 (0.01, 0.02) | 508.75 (368.29, 654.16) | 580.12 (418.38, 741.71) |
| Sweden | 0.00 (0.00, 0.01) | 40.58 (33.97, 48.24) | 45.35 (38.28, 53.38) |  | 0.00 (0.00, 0.01) | 6.63 (5.64, 7.72) | 9.47 (8.08, 10.87) |  | 0.00 (0.00, 0.00) | 2.38 (2.03, 2.72) | 5.14 (4.28, 5.85) |  | 0.06 (0.05, 0.06) | 66.64 (57.35, 75.93) | 108.68 (92.96, 123.72) |
| Switzerland | 0.00 (0.00, 0.00) | 36.72 (32.86, 41.53) | 46.83 (41.97, 52.54) |  | 0.00 (0.00, 0.00) | 5.42 (4.85, 6.06) | 7.97 (7.04, 8.87) |  | 0.00 (0.00, 0.00) | 1.52 (1.33, 1.70) | 3.16 (2.61, 3.57) |  | 0.03 (0.03, 0.04) | 46.08 (41.78, 50.92) | 74.89 (66.29, 83.07) |
| Syrian Arab Republic | 0.00 (0.00, 0.00) | 12.11 (7.97, 16.99) | 12.98 (8.63, 18.22) |  | 0.00 (0.00, 0.00) | 2.86 (1.97, 3.95) | 2.88 (1.97, 4.02) |  | 0.00 (0.00, 0.00) | 1.51 (1.05, 2.04) | 1.38 (0.95, 1.91) |  | 0.03 (0.02, 0.05) | 44.57 (30.38, 61.28) | 45.96 (31.20, 63.76) |
| Taiwan (Province of China) | 0.02 (0.02, 0.02) | 70.60 (60.86, 80.13) | 103.33 (89.33, 117.13) |  | 0.02 (0.02, 0.02) | 10.94 (9.57, 12.21) | 17.09 (15.04, 19.19) |  | 0.01 (0.01, 0.01) | 3.67 (3.21, 4.13) | 6.65 (5.80, 7.50) |  | 0.21 (0.18, 0.23) | 107.54 (94.77, 119.53) | 175.75 (154.89, 196.38) |
| Tajikistan | 0.00 (0.00, 0.00) | 28.66 (18.57, 42.95) | 25.53 (16.42, 38.53) |  | 0.00 (0.00, 0.00) | 6.88 (4.70, 9.89) | 5.43 (3.63, 8.05) |  | 0.00 (0.00, 0.00) | 3.78 (2.72, 5.21) | 2.60 (1.81, 3.71) |  | 0.05 (0.03, 0.07) | 120.69 (82.69, 172.19) | 94.06 (62.57, 137.43) |
| Thailand | 0.11 (0.08, 0.14) | 130.82 (96.01, 175.80) | 177.90 (133.52, 236.68) |  | 0.11 (0.08, 0.14) | 21.93 (16.54, 28.65) | 31.78 (24.15, 41.24) |  | 0.05 (0.03, 0.06) | 8.25 (6.23, 10.64) | 13.39 (10.10, 17.38) |  | 1.40 (1.06, 1.83) | 268.60 (202.54, 350.83) | 405.80 (306.30, 530.20) |
| Timor-Leste | 0.00 (0.00, 0.00) | 55.14 (39.10, 77.38) | 39.51 (27.93, 55.18) |  | 0.00 (0.00, 0.00) | 13.73 (9.95, 18.56) | 9.38 (6.76, 12.72) |  | 0.00 (0.00, 0.00) | 8.22 (6.12, 11.11) | 5.36 (4.01, 7.26) |  | 0.01 (0.01, 0.02) | 266.38 (192.53, 369.70) | 181.67 (131.55, 251.96) |
| Togo | 0.01 (0.01, 0.01) | 117.94 (75.15, 164.19) | 95.19 (59.83, 135.38) |  | 0.01 (0.01, 0.01) | 32.32 (21.20, 43.70) | 22.17 (14.28, 30.03) |  | 0.01 (0.00, 0.01) | 20.57 (14.13, 27.31) | 12.16 (8.03, 16.31) |  | 0.19 (0.12, 0.26) | 650.14 (424.98, 873.26) | 446.59 (288.27, 605.43) |
| Tokelau | 0.00 (0.00, 0.00) | 154.26 (114.06, 201.58) | 153.51 (113.64, 201.34) |  | 0.00 (0.00, 0.00) | 29.57 (22.19, 37.83) | 30.63 (23.01, 39.36) |  | 0.00 (0.00, 0.00) | 12.60 (9.50, 16.22) | 13.77 (10.41, 17.87) |  | 0.00 (0.00, 0.00) | 428.17 (320.12, 552.68) | 448.04 (336.30, 580.58) |
| Tonga | 0.00 (0.00, 0.00) | 168.26 (116.08, 231.90) | 143.56 (98.90, 198.35) |  | 0.00 (0.00, 0.00) | 36.86 (26.45, 49.69) | 30.82 (22.06, 41.70) |  | 0.00 (0.00, 0.00) | 18.63 (13.54, 24.44) | 15.27 (11.12, 20.04) |  | 0.00 (0.00, 0.00) | 556.02 (398.96, 745.33) | 463.62 (331.38, 622.74) |
| Trinidad and Tobago | 0.00 (0.00, 0.00) | 159.60 (116.73, 213.81) | 196.19 (143.71, 261.54) |  | 0.00 (0.00, 0.00) | 28.90 (21.50, 38.01) | 37.23 (27.89, 48.65) |  | 0.00 (0.00, 0.00) | 11.61 (8.86, 14.85) | 15.95 (12.13, 20.38) |  | 0.04 (0.03, 0.05) | 393.04 (291.90, 514.48) | 512.45 (384.17, 667.89) |
| Tunisia | 0.00 (0.00, 0.01) | 27.59 (18.90, 38.16) | 32.78 (22.49, 45.63) |  | 0.00 (0.00, 0.01) | 5.79 (4.01, 7.87) | 6.87 (4.76, 9.37) |  | 0.00 (0.00, 0.00) | 2.65 (1.82, 3.55) | 3.12 (2.13, 4.19) |  | 0.06 (0.04, 0.08) | 84.51 (58.38, 115.13) | 101.09 (69.45, 138.11) |
| Turkmenistan | 0.02 (0.01, 0.02) | 81.20 (59.13, 109.34) | 81.94 (59.53, 110.27) |  | 0.02 (0.01, 0.02) | 3.73 (2.86, 4.77) | 16.40 (12.13, 21.66) |  | 0.01 (0.01, 0.01) | 1.86 (1.43, 2.37) | 7.16 (5.32, 9.57) |  | 0.07 (0.05, 0.09) | 261.97 (193.87, 350.84) | 260.85 (192.56, 350.04) |
| Tuvalu | 0.00 (0.00, 0.01) | 137.77 (98.20, 187.48) | 128.16 (92.00, 173.24) |  | 0.00 (0.00, 0.01) | 16.53 (12.26, 21.79) | 28.31 (20.84, 37.64) |  | 0.00 (0.00, 0.00) | 7.43 (5.55, 9.90) | 14.31 (10.68, 18.80) |  | 0.00 (0.00, 0.00) | 515.57 (381.56, 686.84) | 482.44 (356.21, 641.93) |
| Turkey | 0.00 (0.00, 0.00) | 16.58 (12.48, 21.89) | 19.31 (14.58, 25.25) |  | 0.00 (0.00, 0.00) | 30.42 (22.49, 40.35) | 4.41 (3.38, 5.68) |  | 0.00 (0.00, 0.00) | 15.62 (11.85, 20.40) | 2.22 (1.71, 2.84) |  | 0.27 (0.21, 0.35) | 54.83 (41.67, 70.43) | 65.68 (49.76, 84.56) |
| Uganda | 0.04 (0.03, 0.05) | 125.97 (85.78, 172.42) | 79.21 (53.18, 107.49) |  | 0.04 (0.03, 0.05) | 34.78 (24.22, 46.81) | 18.40 (12.51, 24.70) |  | 0.02 (0.01, 0.03) | 21.78 (15.33, 29.15) | 9.86 (6.80, 13.36) |  | 0.82 (0.55, 1.12) | 709.19 (487.25, 962.18) | 372.11 (250.43, 508.36) |
| Ukraine | 0.03 (0.02, 0.04) | 42.20 (24.99, 63.32) | 62.36 (37.80, 92.38) |  | 0.03 (0.02, 0.04) | 7.93 (4.84, 11.97) | 12.85 (8.09, 18.92) |  | 0.02 (0.01, 0.02) | 3.56 (2.24, 5.19) | 6.51 (4.18, 9.29) |  | 0.44 (0.27, 0.65) | 117.59 (71.48, 175.56) | 191.40 (118.71, 279.49) |
| United Arab Emirates | 0.00 (0.00, 0.00) | 34.09 (26.46, 44.12) | 33.34 (24.05, 45.97) |  | 0.00 (0.00, 0.00) | 15.46 (11.61, 19.92) | 6.69 (4.98, 8.89) |  | 0.00 (0.00, 0.00) | 11.96 (8.69, 15.99) | 2.90 (2.19, 3.83) |  | 0.03 (0.02, 0.04) | 250.69 (186.54, 325.43) | 105.21 (78.78, 142.52) |
| United Kingdom | 0.04 (0.04, 0.05) | 69.79 (66.35, 73.52) | 76.48 (72.94, 80.34) |  | 0.04 (0.04, 0.05) | 10.33 (9.88, 10.81) | 12.66 (12.06, 13.21) |  | 0.01 (0.01, 0.01) | 2.20 (2.07, 2.28) | 3.81 (3.49, 3.99) |  | 0.36 (0.35, 0.38) | 74.54 (71.94, 77.38) | 104.75 (100.33, 108.83) |
| United Republic of Tanzania | 0.06 (0.04, 0.08) | 120.88 (82.50, 167.05) | 84.26 (56.79, 119.07) |  | 0.06 (0.04, 0.08) | 33.29 (22.99, 44.88) | 19.95 (13.49, 27.14) |  | 0.03 (0.02, 0.05) | 21.11 (14.90, 28.55) | 11.09 (7.64, 15.30) |  | 1.22 (0.82, 1.67) | 682.71 (467.75, 944.53) | 407.91 (273.30, 556.56) |
| United States of America | 0.27 (0.26, 0.28) | 84.75 (81.35, 88.46) | 99.69 (95.72, 103.81) |  | 0.27 (0.26, 0.28) | 12.64 (12.16, 13.16) | 16.11 (15.38, 16.79) |  | 0.07 (0.07, 0.08) | 2.69 (2.54, 2.80) | 4.26 (3.95, 4.46) |  | 2.18 (2.08, 2.28) | 94.20 (90.31, 98.36) | 129.06 (123.17, 134.78) |
| United States Virgin Islands | 0.00 (0.00, 0.00) | 78.57 (51.96, 116.22) | 94.39 (64.55, 135.31) |  | 0.00 (0.00, 0.00) | 13.26 (9.12, 18.93) | 18.84 (13.26, 26.38) |  | 0.00 (0.00, 0.00) | 4.77 (3.44, 6.52) | 8.66 (6.26, 11.85) |  | 0.00 (0.00, 0.00) | 162.76 (113.29, 227.75) | 243.83 (173.07, 334.02) |
| Uruguay | 0.01 (0.00, 0.01) | 137.18 (120.92, 155.07) | 152.97 (135.77, 172.69) |  | 0.01 (0.00, 0.01) | 23.13 (20.80, 25.81) | 29.00 (26.20, 32.24) |  | 0.00 (0.00, 0.00) | 8.53 (7.72, 9.36) | 13.12 (11.76, 14.44) |  | 0.07 (0.06, 0.07) | 291.72 (264.32, 323.01) | 378.91 (344.58, 418.58) |
| Uzbekistan | 0.03 (0.02, 0.03) | 70.90 (56.39, 85.96) | 75.09 (59.89, 91.09) |  | 0.03 (0.02, 0.03) | 14.99 (12.14, 17.92) | 15.17 (12.25, 18.13) |  | 0.01 (0.01, 0.01) | 7.11 (5.89, 8.55) | 6.76 (5.59, 8.13) |  | 0.42 (0.34, 0.50) | 242.35 (199.86, 291.93) | 243.90 (200.70, 293.85) |
| Vanuatu | 0.00 (0.00, 0.00) | 97.59 (69.91, 134.14) | 80.91 (57.82, 111.79) |  | 0.00 (0.00, 0.00) | 24.39 (17.99, 32.33) | 18.11 (13.22, 24.23) |  | 0.00 (0.00, 0.00) | 13.88 (10.40, 18.16) | 9.08 (6.78, 11.86) |  | 0.01 (0.00, 0.01) | 457.87 (340.32, 599.94) | 342.59 (251.96, 455.22) |
| Venezuela (Bolivarian Republic of) | 0.08 (0.06, 0.11) | 279.61 (200.47, 382.57) | 319.94 (229.76, 436.80) |  | 0.08 (0.06, 0.11) | 50.15 (35.91, 66.02) | 58.12 (41.65, 76.57) |  | 0.03 (0.02, 0.03) | 15.72 (11.54, 20.43) | 18.51 (13.60, 24.05) |  | 0.88 (0.63, 1.15) | 546.87 (390.29, 712.67) | 640.67 (458.41, 834.12) |
| Viet Nam | 0.10 (0.07, 0.13) | 88.11 (65.32, 116.75) | 104.31 (77.12, 139.00) |  | 0.10 (0.07, 0.13) | 16.50 (12.49, 21.83) | 19.21 (14.44, 25.53) |  | 0.04 (0.03, 0.06) | 7.66 (5.90, 9.94) | 8.69 (6.66, 11.37) |  | 1.37 (1.03, 1.82) | 232.72 (175.02, 307.00) | 271.86 (204.30, 361.17) |
| Yemen | 0.00 (0.00, 0.01) | 16.14 (10.16, 24.17) | 11.84 (7.26, 18.08) |  | 0.00 (0.00, 0.01) | 4.91 (3.22, 7.18) | 2.93 (1.86, 4.36) |  | 0.00 (0.00, 0.00) | 3.24 (2.23, 4.61) | 1.66 (1.10, 2.39) |  | 0.10 (0.06, 0.15) | 102.75 (67.49, 147.52) | 61.17 (38.94, 88.48) |
| Zambia | 0.02 (0.02, 0.05) | 167.32 (101.56, 316.18) | 111.31 (66.36, 218.84) |  | 0.02 (0.02, 0.05) | 45.01 (28.71, 81.92) | 24.96 (15.29, 46.97) |  | 0.01 (0.01, 0.02) | 27.91 (18.27, 48.36) | 13.08 (8.49, 23.85) |  | 0.51 (0.31, 0.96) | 935.50 (601.53, 1706.07) | 513.49 (319.43, 973.55) |
| Zimbabwe | 0.03 (0.02, 0.04) | 201.63 (134.80, 292.54) | 151.46 (100.70, 220.89) |  | 0.03 (0.02, 0.04) | 59.49 (40.34, 81.99) | 38.85 (25.91, 54.95) |  | 0.02 (0.01, 0.03) | 39.05 (26.94, 53.92) | 22.72 (15.38, 31.84) |  | 0.68 (0.45, 0.96) | 1282.03 (865.70, 1811.88) | 841.22 (560.04, 1187.36) |

ASPR, age-standardized prevalence rate; ASIR, age-standardized incidence rate; ASMR, age-standardized mortality rate; ASDR, age-standardized DALY rate

CPR, crude prevalence rate; CIR, crude incidence rate; CMR, crude mortality rate; CDR, crude DALY rate

**Table S2** The average annual percentage change (AAPC) in age-standardized rates (ASRs) of prevalence, incidence, mortality and DALY for cervical cancer in 204 countries and territories from 1991 to 2021.

| **AAPC in ASRs from 1991 to 2021, % (95% CI)** | | | | | | | |
| --- | --- | --- | --- | --- | --- | --- | --- |
|  | **Prevalence** |  | **Incidence** |  | **Mortality** |  | **DALYs** |
| Afghanistan | -0.00 (-0.08, 0.07) |  | -0.51 (-0.56, -0.46) |  | -0.87 (-0.91, -0.83) |  | -0.92 (-0.98, -0.87) |
| Albania | 0.47 (0.32, 0.62) |  | -0.28 (-0.42, -0.13) |  | -1.50 (-1.62, -1.38) |  | -1.65 (-1.78, -1.52) |
| Algeria | -0.68 (-0.71, -0.65) |  | -1.34 (-1.37, -1.30) |  | -2.08 (-2.11, -2.04) |  | -2.26 (-2.28, -2.23) |
| American Samoa | 0.78 (0.70, 0.86) |  | 0.45 (0.38, 0.52) |  | 0.05 (-0.02, 0.13) |  | 0.17 (0.10, 0.25) |
| Andorra | -0.95 (-1.17, -0.73) |  | -1.16 (-1.34, -0.98) |  | -1.91 (-2.11, -1.70) |  | -1.97 (-2.16, -1.77) |
| Angola | 0.41 (0.26, 0.58) |  | -0.34 (-0.45, -0.19) |  | -0.81 (-0.91, -0.71) |  | -0.87 (-0.99, -0.71) |
| Antigua and Barbuda | -0.08 (-0.20, 0.04) |  | -0.31 (-0.46, -0.15) |  | -0.60 (-0.78, -0.42) |  | -0.80 (-0.96, -0.63) |
| Argentina | 0.62 (0.47, 0.76) |  | 0.22 (0.09, 0.35) |  | -0.47 (-0.61, -0.35) |  | -0.52 (-0.65, -0.40) |
| Armenia | -1.80 (-2.01, -1.52) |  | -1.96 (-2.18, -1.71) |  | -2.18 (-2.41, -1.98) |  | -2.50 (-2.75, -2.25) |
| Australia | -1.67 (-1.92, -1.39) |  | -2.11 (-2.37, -1.81) |  | -3.40 (-3.71, -3.04) |  | -3.30 (-3.56, -3.01) |
| Austria | -2.27 (-2.57, -2.06) |  | -2.61 (-2.87, -2.42) |  | -3.06 (-3.24, -2.88) |  | -3.35 (-3.61, -3.10) |
| Azerbaijan | -1.12 (-1.26, -0.96) |  | -1.42 (-1.53, -1.30) |  | -1.74 (-1.84, -1.59) |  | -2.02 (-2.14, -1.85) |
| Bahamas | 0.13 (0.03, 0.25) |  | -0.29 (-0.37, -0.19) |  | -0.84 (-0.94, -0.71) |  | -0.92 (-1.02, -0.79) |
| Bahrain | -0.47 (-0.59, -0.34) |  | -1.60 (-1.65, -1.55) |  | -2.51 (-2.59, -2.44) |  | -2.64 (-2.68, -2.59) |
| Bangladesh | -0.42 (-0.48, -0.33) |  | -1.45 (-1.49, -1.39) |  | -2.22 (-2.27, -2.15) |  | -2.42 (-2.47, -2.36) |
| Barbados | -0.26 (-0.40, -0.10) |  | -0.58 (-0.71, -0.40) |  | -1.00 (-1.17, -0.86) |  | -1.14 (-1.26, -1.03) |
| Belarus | -0.36 (-0.87, 0.17) |  | -1.23 (-1.73, -0.78) |  | -2.48 (-2.95, -2.09) |  | -2.20 (-2.67, -1.81) |
| Belgium | -1.20 (-1.52, -0.97) |  | -1.58 (-1.83, -1.40) |  | -2.03 (-2.21, -1.85) |  | -2.21 (-2.40, -2.06) |
| Belize | 0.61 (0.38, 0.83) |  | 0.25 (0.07, 0.42) |  | -0.21 (-0.38, -0.03) |  | -0.20 (-0.36, -0.04) |
| Benin | 0.14 (0.08, 0.19) |  | -0.27 (-0.32, -0.22) |  | -0.53 (-0.58, -0.49) |  | -0.67 (-0.73, -0.63) |
| Bermuda | -0.72 (-0.89, -0.56) |  | -1.44 (-1.57, -1.33) |  | -2.81 (-2.92, -2.69) |  | -2.77 (-2.90, -2.66) |
| Bhutan | -0.61 (-0.67, -0.54) |  | -1.55 (-1.59, -1.50) |  | -2.19 (-2.23, -2.15) |  | -2.42 (-2.46, -2.37) |
| Bolivia (Plurinational State of) | -0.28 (-0.32, -0.25) |  | -1.13 (-1.17, -1.09) |  | -1.64 (-1.67, -1.60) |  | -1.86 (-1.90, -1.83) |
| Bosnia and Herzegovina | 0.41 (0.22, 0.59) |  | -0.18 (-0.37, 0.01) |  | -1.17 (-1.32, -1.01) |  | -1.20 (-1.33, -1.05) |
| Botswana | -0.56 (-0.78, -0.29) |  | -0.92 (-1.13, -0.65) |  | -1.18 (-1.40, -0.90) |  | -1.32 (-1.56, -1.01) |
| Brazil | 0.42 (0.36, 0.49) |  | -0.49 (-0.56, -0.42) |  | -1.56 (-1.61, -1.50) |  | -1.35 (-1.41, -1.30) |
| Brunei Darussalam | -0.91 (-0.96, -0.86) |  | -1.32 (-1.36, -1.27) |  | -1.78 (-1.85, -1.72) |  | -1.87 (-1.92, -1.83) |
| Bulgaria | 0.19 (0.07, 0.33) |  | 0.14 (-0.00, 0.26) |  | -0.41 (-0.53, -0.27) |  | -0.40 (-0.58, -0.25) |
| Burkina Faso | -0.23 (-0.31, -0.16) |  | -0.54 (-0.60, -0.49) |  | -0.70 (-0.77, -0.64) |  | -0.83 (-0.90, -0.77) |
| Burundi | -1.21 (-1.25, -1.16) |  | -1.57 (-1.62, -1.53) |  | -1.77 (-1.83, -1.71) |  | -1.91 (-1.95, -1.86) |
| Cabo Verde | -0.75 (-0.79, -0.69) |  | -1.08 (-1.13, -1.02) |  | -1.26 (-1.29, -1.23) |  | -1.61 (-1.64, -1.57) |
| Cambodia | -0.08 (-0.10, -0.05) |  | -0.86 (-0.88, -0.84) |  | -1.50 (-1.51, -1.49) |  | -1.63 (-1.65, -1.61) |
| Cameroon | -0.00 (-0.07, 0.06) |  | -0.31 (-0.36, -0.25) |  | -0.56 (-0.59, -0.53) |  | -0.69 (-0.72, -0.65) |
| Canada | 0.67 (0.40, 0.88) |  | 0.33 (0.05, 0.55) |  | -0.99 (-1.27, -0.74) |  | -0.73 (-1.03, -0.44) |
| Central African Republic | -0.30 (-0.38, -0.19) |  | -0.51 (-0.58, -0.42) |  | -0.60 (-0.66, -0.52) |  | -0.66 (-0.75, -0.56) |
| Chad | 0.97 (0.91, 1.05) |  | 0.77 (0.73, 0.82) |  | 0.61 (0.57, 0.65) |  | 0.53 (0.47, 0.58) |
| Chile | -0.81 (-0.96, -0.70) |  | -1.84 (-2.00, -1.73) |  | -3.28 (-3.40, -3.18) |  | -3.16 (-3.28, -3.06) |
| China | 2.01 (1.96, 2.06) |  | 0.52 (0.48, 0.56) |  | -1.22 (-1.27, -1.17) |  | -1.26 (-1.32, -1.22) |
| Colombia | 0.93 (0.83, 1.04) |  | -0.29 (-0.42, -0.16) |  | -2.02 (-2.11, -1.92) |  | -1.79 (-1.89, -1.69) |
| Comoros | -0.77 (-1.02, -0.53) |  | -1.06 (-1.22, -0.89) |  | -1.19 (-1.32, -1.02) |  | -1.31 (-1.53, -1.06) |
| Congo | 0.47 (0.36, 0.59) |  | -0.32 (-0.41, -0.23) |  | -0.85 (-0.93, -0.77) |  | -0.83 (-0.92, -0.74) |
| Cook Islands | -0.41 (-0.44, -0.38) |  | -1.03 (-1.07, -0.99) |  | -1.88 (-1.91, -1.85) |  | -1.93 (-1.96, -1.90) |
| Costa Rica | -0.63 (-0.88, -0.37) |  | -1.31 (-1.59, -1.03) |  | -2.49 (-2.81, -2.18) |  | -2.23 (-2.52, -1.94) |
| Croatia | -0.99 (-1.20, -0.76) |  | -1.38 (-1.60, -1.15) |  | -2.32 (-2.54, -2.09) |  | -2.30 (-2.52, -2.08) |
| Cuba | -0.27 (-0.43, -0.09) |  | -0.51 (-0.65, -0.33) |  | -1.14 (-1.24, -1.01) |  | -1.15 (-1.27, -1.02) |
| Cyprus | -0.43 (-0.58, -0.26) |  | -1.08 (-1.20, -0.95) |  | -2.38 (-2.50, -2.27) |  | -2.27 (-2.38, -2.15) |
| Czechia | -1.87 (-2.08, -1.66) |  | -2.31 (-2.50, -2.18) |  | -2.85 (-3.00, -2.76) |  | -3.08 (-3.24, -2.98) |
| Cote d'Ivoire | 0.44 (0.38, 0.49) |  | -0.11 (-0.15, -0.06) |  | -0.48 (-0.52, -0.44) |  | -0.49 (-0.53, -0.44) |
| Democratic People's Republic of Korea | 1.18 (1.16, 1.21) |  | 0.64 (0.62, 0.66) |  | -0.07 (-0.08, -0.06) |  | 0.04 (0.03, 0.05) |
| Democratic Republic of the Congo | 0.48 (0.40, 0.56) |  | 0.03 (-0.06, 0.11) |  | -0.25 (-0.31, -0.19) |  | -0.31 (-0.38, -0.24) |
| Denmark | -2.33 (-2.49, -2.21) |  | -2.81 (-2.99, -2.68) |  | -3.67 (-3.86, -3.52) |  | -3.84 (-3.97, -3.73) |
| Djibouti | 0.18 (0.05, 0.34) |  | -0.33 (-0.40, -0.25) |  | -0.58 (-0.64, -0.52) |  | -0.69 (-0.77, -0.59) |
| Dominica | -0.23 (-0.27, -0.19) |  | -0.56 (-0.59, -0.52) |  | -0.87 (-0.89, -0.84) |  | -0.87 (-0.90, -0.83) |
| Dominican Republic | 0.80 (0.71, 0.89) |  | 0.17 (0.09, 0.25) |  | -0.52 (-0.61, -0.44) |  | -0.50 (-0.59, -0.41) |
| Ecuador | 0.27 (0.08, 0.53) |  | -0.49 (-0.66, -0.29) |  | -1.35 (-1.53, -1.19) |  | -1.55 (-1.74, -1.32) |
| Egypt | 1.92 (1.76, 2.05) |  | 1.71 (1.57, 1.84) |  | 1.33 (1.19, 1.47) |  | 0.97 (0.81, 1.11) |
| El Salvador | 1.61 (1.33, 1.89) |  | 0.67 (0.46, 0.90) |  | -0.55 (-0.69, -0.39) |  | -0.65 (-0.82, -0.46) |
| Equatorial Guinea | 0.86 (0.76, 0.99) |  | -0.43 (-0.53, -0.32) |  | -1.37 (-1.46, -1.27) |  | -1.48 (-1.57, -1.38) |
| Eritrea | -0.21 (-0.26, -0.15) |  | -0.66 (-0.69, -0.61) |  | -0.90 (-0.93, -0.87) |  | -1.03 (-1.06, -0.98) |
| Estonia | -1.16 (-1.59, -0.62) |  | -1.49 (-1.71, -1.26) |  | -2.63 (-2.79, -2.46) |  | -2.82 (-2.99, -2.63) |
| Eswatini | 1.59 (1.53, 1.66) |  | 1.32 (1.26, 1.39) |  | 1.08 (1.03, 1.15) |  | 1.09 (1.02, 1.16) |
| Ethiopia | -1.50 (-1.54, -1.47) |  | -2.37 (-2.40, -2.33) |  | -2.87 (-2.90, -2.83) |  | -3.07 (-3.12, -3.01) |
| Fiji | -0.51 (-0.59, -0.40) |  | -0.67 (-0.75, -0.59) |  | -0.71 (-0.79, -0.62) |  | -0.84 (-0.92, -0.76) |
| Finland | -0.26 (-0.44, -0.07) |  | -0.85 (-1.07, -0.65) |  | -2.29 (-2.52, -2.05) |  | -1.93 (-2.10, -1.76) |
| France | -0.54 (-0.64, -0.46) |  | -1.04 (-1.12, -0.97) |  | -2.11 (-2.20, -2.03) |  | -2.10 (-2.16, -2.05) |
| Gabon | 0.37 (0.31, 0.45) |  | -0.20 (-0.27, -0.13) |  | -0.72 (-0.80, -0.65) |  | -0.88 (-0.95, -0.78) |
| Gambia | 0.91 (0.62, 1.28) |  | 0.60 (0.35, 0.94) |  | 0.23 (0.04, 0.48) |  | 0.25 (0.02, 0.56) |
| Georgia | -0.36 (-0.67, -0.01) |  | -0.21 (-0.53, 0.16) |  | -0.26 (-0.65, 0.24) |  | -0.61 (-1.01, -0.22) |
| Germany | -0.94 (-1.05, -0.85) |  | -1.46 (-1.60, -1.34) |  | -2.73 (-2.89, -2.62) |  | -2.55 (-2.66, -2.46) |
| Ghana | -0.03 (-0.06, 0.01) |  | -0.23 (-0.26, -0.20) |  | -0.39 (-0.42, -0.35) |  | -0.66 (-0.70, -0.63) |
| Greece | -1.24 (-1.44, -0.96) |  | -1.42 (-1.63, -1.15) |  | -1.81 (-1.96, -1.59) |  | -1.72 (-1.91, -1.45) |
| Greenland | -1.17 (-1.26, -1.08) |  | -1.83 (-1.92, -1.73) |  | -3.02 (-3.11, -2.91) |  | -2.97 (-3.05, -2.88) |
| Grenada | 0.04 (-0.08, 0.16) |  | -0.31 (-0.42, -0.19) |  | -0.63 (-0.76, -0.49) |  | -0.91 (-1.01, -0.80) |
| Guam | -0.28 (-0.39, -0.18) |  | -1.03 (-1.10, -0.95) |  | -2.34 (-2.48, -2.22) |  | -1.41 (-1.48, -1.34) |
| Guatemala | 1.35 (1.06, 1.66) |  | 0.59 (0.32, 0.86) |  | -0.24 (-0.52, 0.05) |  | -0.35 (-0.60, -0.07) |
| Guinea | 0.03 (-0.02, 0.09) |  | -0.32 (-0.37, -0.27) |  | -0.58 (-0.62, -0.54) |  | -0.64 (-0.68, -0.60) |
| Guinea-Bissau | 0.44 (0.41, 0.47) |  | 0.19 (0.16, 0.22) |  | 0.01 (-0.01, 0.04) |  | -0.17 (-0.20, -0.14) |
| Guyana | 0.21 (-0.01, 0.43) |  | -0.32 (-0.54, -0.11) |  | -0.74 (-0.97, -0.50) |  | -0.87 (-1.17, -0.53) |
| Haiti | -0.53 (-0.67, -0.35) |  | -1.04 (-1.07, -1.00) |  | -1.35 (-1.38, -1.32) |  | -1.44 (-1.47, -1.40) |
| Honduras | 0.66 (0.48, 0.84) |  | 0.58 (0.44, 0.74) |  | 0.63 (0.46, 0.85) |  | 0.06 (-0.08, 0.24) |
| Hungary | -0.73 (-0.91, -0.52) |  | -1.04 (-1.19, -0.88) |  | -1.89 (-2.04, -1.75) |  | -2.02 (-2.16, -1.87) |
| Iceland | -1.52 (-1.72, -1.31) |  | -1.81 (-2.00, -1.61) |  | -2.79 (-3.01, -2.62) |  | -2.91 (-3.13, -2.73) |
| India | -0.69 (-0.75, -0.64) |  | -1.40 (-1.45, -1.35) |  | -1.97 (-2.03, -1.91) |  | -2.14 (-2.20, -2.10) |
| Indonesia | 0.07 (0.04, 0.10) |  | -0.41 (-0.43, -0.39) |  | -0.83 (-0.84, -0.82) |  | -0.99 (-1.01, -0.98) |
| Iran (Islamic Republic of) | -0.94 (-1.04, -0.84) |  | -1.49 (-1.56, -1.41) |  | -2.11 (-2.18, -2.02) |  | -2.32 (-2.41, -2.22) |
| Iraq | 0.14 (0.10, 0.19) |  | -0.32 (-0.36, -0.27) |  | -0.89 (-0.94, -0.83) |  | -1.18 (-1.22, -1.13) |
| Ireland | -0.57 (-0.91, -0.22) |  | -1.08 (-1.41, -0.74) |  | -2.29 (-2.51, -2.05) |  | -2.19 (-2.41, -1.93) |
| Israel | -0.85 (-1.00, -0.74) |  | -1.32 (-1.50, -1.11) |  | -2.04 (-2.32, -1.78) |  | -2.19 (-2.35, -1.97) |
| Italy | 2.39 (2.01, 2.66) |  | 1.86 (1.45, 2.21) |  | 1.06 (0.85, 1.27) |  | 1.01 (0.79, 1.22) |
| Jamaica | 0.22 (0.06, 0.38) |  | -0.21 (-0.36, -0.07) |  | -0.72 (-0.86, -0.59) |  | -0.62 (-0.75, -0.49) |
| Japan | 1.03 (0.91, 1.18) |  | 0.52 (0.40, 0.72) |  | -0.84 (-0.91, -0.76) |  | -0.24 (-0.32, -0.15) |
| Jordan | -1.74 (-1.90, -1.60) |  | -2.26 (-2.43, -2.14) |  | -2.91 (-3.10, -2.75) |  | -3.22 (-3.32, -3.12) |
| Kazakhstan | 0.80 (0.66, 0.96) |  | -0.08 (-0.26, 0.09) |  | -1.29 (-1.50, -1.08) |  | -1.00 (-1.14, -0.80) |
| Kenya | 0.64 (0.59, 0.70) |  | 0.10 (0.04, 0.16) |  | -0.10 (-0.15, -0.06) |  | -0.18 (-0.23, -0.14) |
| Kiribati | 0.11 (0.08, 0.14) |  | -0.16 (-0.17, -0.15) |  | -0.32 (-0.34, -0.31) |  | -0.44 (-0.46, -0.43) |
| Kuwait | -3.73 (-4.00, -3.44) |  | -4.07 (-4.31, -3.82) |  | -4.57 (-4.81, -4.30) |  | -4.89 (-5.13, -4.65) |
| Kyrgyzstan | -0.46 (-0.66, -0.24) |  | -0.86 (-1.04, -0.64) |  | -1.46 (-1.68, -1.30) |  | -1.54 (-1.77, -1.30) |
| Lao People's Democratic Republic | -0.87 (-0.89, -0.84) |  | -1.67 (-1.70, -1.64) |  | -2.25 (-2.29, -2.22) |  | -2.37 (-2.41, -2.34) |
| Latvia | -0.84 (-1.10, -0.43) |  | -1.51 (-1.81, -1.19) |  | -1.85 (-2.14, -1.56) |  | -2.10 (-2.40, -1.80) |
| Lebanon | -1.59 (-1.70, -1.48) |  | -2.17 (-2.25, -2.07) |  | -2.94 (-3.05, -2.84) |  | -3.31 (-3.54, -3.20) |
| Lesotho | 3.05 (2.87, 3.21) |  | 2.93 (2.78, 3.08) |  | 2.78 (2.67, 2.88) |  | 2.91 (2.79, 3.02) |
| Liberia | 1.35 (1.28, 1.42) |  | 0.58 (0.52, 0.64) |  | 0.06 (0.01, 0.12) |  | 0.02 (-0.04, 0.09) |
| Libya | 0.54 (0.42, 0.66) |  | 0.05 (-0.02, 0.14) |  | -0.39 (-0.47, -0.31) |  | -0.46 (-0.52, -0.39) |
| Lithuania | -0.80 (-0.97, -0.64) |  | -1.00 (-1.15, -0.86) |  | -1.45 (-1.68, -1.23) |  | -1.59 (-1.73, -1.44) |
| Luxembourg | -2.25 (-2.44, -1.99) |  | -2.49 (-2.70, -2.32) |  | -3.26 (-3.44, -3.08) |  | -3.53 (-3.69, -3.38) |
| Madagascar | -0.37 (-0.42, -0.31) |  | -0.69 (-0.74, -0.64) |  | -0.92 (-0.96, -0.87) |  | -1.00 (-1.04, -0.95) |
| Malawi | 0.87 (0.82, 0.91) |  | 0.28 (0.22, 0.33) |  | -0.13 (-0.19, -0.07) |  | -0.15 (-0.21, -0.10) |
| Malaysia | 0.58 (0.39, 0.77) |  | -0.23 (-0.41, -0.06) |  | -1.03 (-1.20, -0.86) |  | -1.06 (-1.24, -0.89) |
| Maldives | -2.92 (-3.01, -2.84) |  | -3.90 (-3.99, -3.81) |  | -4.84 (-4.96, -4.69) |  | -5.26 (-5.34, -5.17) |
| Mali | -0.40 (-0.45, -0.33) |  | -0.78 (-0.83, -0.74) |  | -1.08 (-1.12, -1.04) |  | -1.18 (-1.23, -1.13) |
| Malta | -0.55 (-0.86, -0.21) |  | -1.15 (-1.42, -0.88) |  | -2.44 (-2.65, -2.26) |  | -2.27 (-2.47, -2.10) |
| Marshall Islands | 0.35 (0.33, 0.38) |  | -0.02 (-0.04, -0.00) |  | -0.37 (-0.38, -0.36) |  | -0.29 (-0.30, -0.28) |
| Mauritania | 0.67 (0.59, 0.78) |  | -0.15 (-0.24, -0.06) |  | -0.71 (-0.76, -0.65) |  | -0.87 (-0.94, -0.78) |
| Mauritius | -1.01 (-1.34, -0.62) |  | -1.45 (-1.80, -1.01) |  | -2.07 (-2.40, -1.68) |  | -2.09 (-2.41, -1.69) |
| Mexico | -1.22 (-1.29, -1.14) |  | -2.29 (-2.36, -2.22) |  | -3.66 (-3.75, -3.55) |  | -3.27 (-3.36, -3.18) |
| Micronesia (Federated States of) | 0.11 (0.10, 0.13) |  | -0.45 (-0.46, -0.43) |  | -0.91 (-0.92, -0.90) |  | -0.90 (-0.91, -0.90) |
| Monaco | 0.01 (-0.02, 0.04) |  | -0.26 (-0.28, -0.23) |  | -1.02 (-1.04, -0.99) |  | -1.02 (-1.05, -1.00) |
| Mongolia | -0.00 (-0.13, 0.13) |  | -0.80 (-1.00, -0.60) |  | -1.45 (-1.57, -1.33) |  | -1.65 (-1.78, -1.50) |
| Montenegro | -0.14 (-0.26, -0.03) |  | -0.18 (-0.28, -0.09) |  | -0.40 (-0.49, -0.31) |  | -0.68 (-0.74, -0.62) |
| Morocco | 0.65 (0.61, 0.70) |  | -0.05 (-0.08, -0.02) |  | -0.62 (-0.65, -0.59) |  | -0.71 (-0.74, -0.67) |
| Mozambique | 0.51 (0.47, 0.56) |  | 0.26 (0.21, 0.31) |  | 0.06 (0.01, 0.11) |  | -0.03 (-0.08, 0.03) |
| Myanmar | -1.23 (-1.26, -1.20) |  | -1.95 (-1.98, -1.92) |  | -2.51 (-2.54, -2.49) |  | -2.71 (-2.74, -2.67) |
| Namibia | 1.80 (1.68, 1.90) |  | 1.13 (1.04, 1.21) |  | 0.63 (0.53, 0.71) |  | 0.59 (0.50, 0.68) |
| Nauru | 0.15 (0.12, 0.21) |  | -0.23 (-0.26, -0.20) |  | -0.61 (-0.65, -0.56) |  | -0.56 (-0.60, -0.51) |
| Nepal | -0.45 (-0.49, -0.41) |  | -1.38 (-1.42, -1.33) |  | -2.00 (-2.03, -1.96) |  | -2.15 (-2.21, -2.10) |
| Netherlands | -0.72 (-0.85, -0.52) |  | -0.97 (-1.13, -0.79) |  | -1.80 (-1.97, -1.56) |  | -1.90 (-2.07, -1.74) |
| New Zealand | -3.10 (-3.33, -2.73) |  | -3.30 (-3.49, -3.09) |  | -4.38 (-4.60, -4.09) |  | -4.26 (-4.45, -3.98) |
| Nicaragua | -0.74 (-1.02, -0.51) |  | -1.29 (-1.52, -1.11) |  | -1.97 (-2.12, -1.82) |  | -2.20 (-2.41, -2.01) |
| Niger | 0.03 (-0.01, 0.07) |  | -0.14 (-0.19, -0.11) |  | -0.28 (-0.32, -0.24) |  | -0.48 (-0.53, -0.44) |
| Nigeria | -0.11 (-0.15, -0.07) |  | -0.44 (-0.48, -0.41) |  | -0.74 (-0.76, -0.71) |  | -0.94 (-0.97, -0.90) |
| Niue | 0.39 (0.19, 0.60) |  | -0.16 (-0.30, -0.04) |  | -0.87 (-0.94, -0.81) |  | -0.76 (-0.90, -0.64) |
| North Macedonia | -0.07 (-0.18, 0.04) |  | -0.44 (-0.56, -0.33) |  | -1.01 (-1.10, -0.92) |  | -1.23 (-1.35, -1.09) |
| Northern Mariana Islands | -0.67 (-0.73, -0.60) |  | -0.88 (-0.99, -0.72) |  | -1.31 (-1.64, -0.91) |  | -1.30 (-1.50, -1.13) |
| Norway | -1.07 (-1.28, -0.86) |  | -1.46 (-1.62, -1.31) |  | -2.17 (-2.45, -1.91) |  | -2.51 (-2.72, -2.35) |
| Oman | -1.53 (-1.68, -1.37) |  | -2.20 (-2.37, -2.01) |  | -2.94 (-3.00, -2.88) |  | -3.20 (-3.30, -3.11) |
| Pakistan | 0.63 (0.61, 0.67) |  | 0.13 (0.10, 0.16) |  | -0.31 (-0.33, -0.29) |  | -0.30 (-0.32, -0.28) |
| Palau | -0.52 (-0.56, -0.48) |  | -0.73 (-0.78, -0.70) |  | -0.88 (-0.92, -0.83) |  | -1.17 (-1.20, -1.14) |
| Palestine | -0.83 (-0.87, -0.77) |  | -1.35 (-1.41, -1.30) |  | -1.88 (-1.95, -1.82) |  | -2.09 (-2.14, -2.05) |
| Panama | -0.80 (-0.95, -0.63) |  | -1.42 (-1.58, -1.22) |  | -2.51 (-2.73, -2.27) |  | -2.44 (-2.64, -2.22) |
| Papua New Guinea | -0.60 (-0.67, -0.52) |  | -0.74 (-0.81, -0.63) |  | -0.82 (-0.89, -0.75) |  | -0.90 (-0.95, -0.84) |
| Paraguay | 0.48 (0.32, 0.67) |  | 0.25 (0.10, 0.40) |  | -0.24 (-0.41, -0.08) |  | -0.41 (-0.65, -0.23) |
| Peru | 1.45 (0.97, 1.99) |  | 0.07 (-0.16, 0.31) |  | -1.01 (-1.28, -0.74) |  | -1.13 (-1.40, -0.85) |
| Philippines | 0.51 (0.45, 0.58) |  | 0.27 (0.21, 0.33) |  | -0.04 (-0.10, 0.01) |  | -0.06 (-0.14, 0.03) |
| Poland | -1.66 (-1.80, -1.50) |  | -2.15 (-2.27, -2.02) |  | -2.60 (-2.69, -2.48) |  | -3.00 (-3.09, -2.89) |
| Portugal | -0.52 (-0.73, -0.29) |  | -1.10 (-1.27, -0.91) |  | -2.57 (-2.75, -2.40) |  | -2.42 (-2.56, -2.25) |
| Puerto Rico | 0.40 (0.06, 0.74) |  | -0.28 (-0.54, -0.03) |  | -1.54 (-1.73, -1.34) |  | -1.34 (-1.54, -1.14) |
| Qatar | -1.45 (-1.63, -1.29) |  | -1.75 (-1.88, -1.61) |  | -2.47 (-2.67, -2.24) |  | -2.82 (-2.95, -2.69) |
| Republic of Korea | -0.86 (-0.93, -0.77) |  | -1.70 (-1.76, -1.62) |  | -3.41 (-3.50, -3.34) |  | -3.69 (-3.75, -3.63) |
| Republic of Moldova | -0.14 (-0.45, 0.10) |  | -0.89 (-1.17, -0.68) |  | -1.71 (-1.95, -1.49) |  | -1.53 (-1.79, -1.30) |
| Romania | 0.08 (-0.04, 0.20) |  | -0.33 (-0.45, -0.20) |  | -0.93 (-1.01, -0.83) |  | -1.24 (-1.34, -1.14) |
| Russian Federation | 2.43 (2.31, 2.57) |  | 1.44 (1.33, 1.57) |  | -0.13 (-0.24, -0.01) |  | 0.49 (0.36, 0.61) |
| Rwanda | -1.83 (-2.13, -1.34) |  | -2.36 (-2.42, -2.30) |  | -2.77 (-2.83, -2.70) |  | -3.02 (-3.09, -2.95) |
| Saint Kitts and Nevis | -1.71 (-1.83, -1.61) |  | -1.83 (-1.93, -1.72) |  | -1.97 (-2.06, -1.85) |  | -2.64 (-2.73, -2.54) |
| Saint Lucia | -0.96 (-1.09, -0.83) |  | -1.60 (-1.76, -1.46) |  | -2.37 (-2.48, -2.27) |  | -2.40 (-2.51, -2.28) |
| Saint Vincent and the Grenadines | -0.58 (-0.73, -0.39) |  | -1.05 (-1.18, -0.93) |  | -1.56 (-1.70, -1.46) |  | -1.56 (-1.68, -1.44) |
| Samoa | 1.08 (1.06, 1.10) |  | 0.58 (0.57, 0.59) |  | 0.02 (0.00, 0.04) |  | 0.13 (0.12, 0.14) |
| San Marino | -1.42 (-1.70, -1.20) |  | -1.76 (-2.03, -1.47) |  | -2.50 (-2.78, -2.30) |  | -2.24 (-2.49, -2.05) |
| Sao Tome and Principe | 0.67 (0.49, 0.85) |  | 0.14 (0.04, 0.26) |  | -0.33 (-0.44, -0.23) |  | -0.44 (-0.54, -0.33) |
| Saudi Arabia | 0.41 (0.36, 0.45) |  | -0.33 (-0.39, -0.27) |  | -2.00 (-2.04, -1.96) |  | -1.95 (-1.99, -1.91) |
| Senegal | 0.50 (0.33, 0.68) |  | 0.10 (-0.10, 0.27) |  | -0.07 (-0.23, 0.06) |  | -0.28 (-0.48, -0.12) |
| Serbia | -0.28 (-0.54, -0.03) |  | -0.62 (-0.80, -0.40) |  | -1.50 (-1.63, -1.33) |  | -1.55 (-1.70, -1.35) |
| Seychelles | -0.36 (-0.46, -0.22) |  | -0.97 (-1.01, -0.93) |  | -1.56 (-1.65, -1.47) |  | -1.64 (-1.67, -1.60) |
| Sierra Leone | 1.50 (1.45, 1.55) |  | 1.01 (0.97, 1.05) |  | 0.67 (0.64, 0.71) |  | 0.67 (0.62, 0.72) |
| Singapore | -1.83 (-2.09, -1.54) |  | -2.32 (-2.64, -1.98) |  | -3.37 (-3.55, -3.20) |  | -3.52 (-3.72, -3.34) |
| Slovakia | -0.78 (-0.99, -0.54) |  | -1.01 (-1.23, -0.81) |  | -1.47 (-1.57, -1.35) |  | -1.72 (-1.87, -1.49) |
| Slovenia | -1.26 (-1.69, -0.90) |  | -1.59 (-1.97, -1.24) |  | -2.62 (-2.86, -2.37) |  | -2.72 (-3.02, -2.43) |
| Solomon Islands | 0.46 (0.37, 0.55) |  | -0.07 (-0.15, -0.01) |  | -0.42 (-0.50, -0.36) |  | -0.31 (-0.39, -0.25) |
| Somalia | -0.31 (-0.32, -0.29) |  | -0.43 (-0.45, -0.41) |  | -0.49 (-0.50, -0.47) |  | -0.60 (-0.62, -0.57) |
| South Africa | 0.78 (0.43, 1.18) |  | 1.03 (0.79, 1.29) |  | 1.14 (0.89, 1.46) |  | 0.77 (0.52, 1.04) |
| South Sudan | 0.61 (0.52, 0.71) |  | 0.26 (0.20, 0.33) |  | -0.03 (-0.07, 0.03) |  | -0.04 (-0.10, 0.03) |
| Spain | -0.88 (-1.03, -0.76) |  | -1.19 (-1.32, -1.07) |  | -2.23 (-2.34, -2.14) |  | -2.21 (-2.35, -2.10) |
| Sri Lanka | -0.16 (-0.50, 0.27) |  | -0.73 (-0.89, -0.58) |  | -1.51 (-1.65, -1.38) |  | -1.75 (-1.93, -1.60) |
| Sudan | -0.30 (-0.33, -0.26) |  | -0.93 (-0.95, -0.91) |  | -1.49 (-1.50, -1.48) |  | -1.62 (-1.64, -1.61) |
| Suriname | -0.03 (-0.16, 0.11) |  | -0.62 (-0.73, -0.49) |  | -1.19 (-1.34, -1.04) |  | -1.06 (-1.20, -0.93) |
| Sweden | -0.92 (-1.22, -0.66) |  | -1.15 (-1.33, -0.94) |  | -1.51 (-1.67, -1.39) |  | -1.75 (-1.91, -1.57) |
| Switzerland | -2.25 (-2.43, -2.06) |  | -2.59 (-2.75, -2.42) |  | -3.44 (-3.60, -3.29) |  | -3.49 (-3.65, -3.33) |
| Syrian Arab Republic | -0.35 (-0.46, -0.23) |  | -0.83 (-0.96, -0.72) |  | -1.42 (-1.49, -1.33) |  | -1.73 (-1.88, -1.58) |
| Taiwan (Province of China) | -2.93 (-3.06, -2.82) |  | -3.38 (-3.50, -3.28) |  | -4.24 (-4.36, -4.15) |  | -4.35 (-4.45, -4.25) |
| Tajikistan | -1.80 (-1.87, -1.73) |  | -1.81 (-1.86, -1.74) |  | -1.92 (-1.97, -1.85) |  | -2.11 (-2.17, -2.03) |
| Thailand | -0.02 (-0.12, 0.09) |  | -0.92 (-1.02, -0.81) |  | -2.11 (-2.19, -2.02) |  | -2.04 (-2.16, -1.91) |
| Timor-Leste | 0.03 (-0.05, 0.15) |  | -0.63 (-0.70, -0.56) |  | -1.05 (-1.12, -0.99) |  | -1.12 (-1.19, -1.06) |
| Togo | 0.35 (0.30, 0.40) |  | 0.10 (0.06, 0.14) |  | -0.07 (-0.11, -0.04) |  | -0.25 (-0.28, -0.22) |
| Tokelau | 0.44 (0.29, 0.58) |  | -0.44 (-0.55, -0.36) |  | -1.38 (-1.42, -1.34) |  | -1.21 (-1.26, -1.13) |
| Tonga | -0.41 (-0.43, -0.38) |  | -0.64 (-0.67, -0.61) |  | -0.79 (-0.81, -0.76) |  | -0.97 (-1.00, -0.94) |
| Trinidad and Tobago | 0.54 (0.37, 0.78) |  | -0.47 (-0.63, -0.23) |  | -1.35 (-1.58, -1.11) |  | -1.08 (-1.36, -0.80) |
| Tunisia | -0.22 (-0.26, -0.17) |  | -0.78 (-0.81, -0.73) |  | -1.54 (-1.58, -1.49) |  | -1.65 (-1.69, -1.60) |
| Turkmenistan | 1.14 (0.95, 1.32) |  | -1.58 (-1.63, -1.52) |  | -2.38 (-2.43, -2.33) |  | 0.17 (-0.01, 0.36) |
| Tuvalu | -0.15 (-0.18, -0.13) |  | 0.71 (0.53, 0.90) |  | 0.10 (-0.06, 0.27) |  | -1.39 (-1.42, -1.37) |
| Turkey | -0.83 (-0.90, -0.76) |  | -0.79 (-0.82, -0.76) |  | -1.34 (-1.36, -1.32) |  | -2.82 (-2.86, -2.77) |
| Uganda | 0.16 (0.10, 0.24) |  | -0.39 (-0.45, -0.31) |  | -0.75 (-0.80, -0.69) |  | -0.76 (-0.82, -0.69) |
| Ukraine | -2.94 (-3.25, -2.66) |  | -3.10 (-3.39, -2.78) |  | -3.50 (-3.75, -3.22) |  | -3.42 (-3.68, -3.14) |
| United Arab Emirates | -1.36 (-1.46, -1.25) |  | -0.66 (-0.89, -0.44) |  | -0.20 (-0.51, 0.10) |  | -1.46 (-1.69, -1.26) |
| United Kingdom | -3.20 (-3.34, -3.03) |  | -3.38 (-3.53, -3.22) |  | -3.16 (-3.26, -3.04) |  | -3.21 (-3.34, -3.05) |
| United Republic of Tanzania | -0.48 (-0.50, -0.44) |  | -0.83 (-0.85, -0.79) |  | -1.07 (-1.09, -1.03) |  | -1.18 (-1.20, -1.14) |
| United States of America | -1.52 (-1.58, -1.44) |  | -1.53 (-1.59, -1.49) |  | -1.11 (-1.18, -1.07) |  | -1.18 (-1.25, -1.14) |
| United States Virgin Islands | -1.52 (-1.69, -1.27) |  | -2.16 (-2.26, -2.02) |  | -3.00 (-3.09, -2.93) |  | -2.76 (-2.83, -2.69) |
| Uruguay | 0.63 (0.42, 0.90) |  | 0.22 (-0.00, 0.50) |  | -0.49 (-0.72, -0.20) |  | -0.57 (-0.80, -0.28) |
| Uzbekistan | 0.29 (0.15, 0.44) |  | 0.15 (-0.02, 0.32) |  | -0.14 (-0.27, -0.01) |  | -0.23 (-0.37, -0.10) |
| Vanuatu | -0.22 (-0.30, -0.12) |  | -0.44 (-0.52, -0.35) |  | -0.62 (-0.67, -0.55) |  | -0.59 (-0.66, -0.51) |
| Venezuela (Bolivarian Republic of) | 1.02 (0.87, 1.19) |  | 0.35 (0.21, 0.50) |  | -0.74 (-0.91, -0.55) |  | -0.59 (-0.74, -0.41) |
| Viet Nam | 0.70 (0.65, 0.76) |  | 0.06 (-0.00, 0.13) |  | -0.61 (-0.71, -0.50) |  | -0.90 (-0.97, -0.82) |
| Yemen | -0.25 (-0.41, -0.13) |  | -0.71 (-0.80, -0.63) |  | -1.01 (-1.08, -0.95) |  | -1.12 (-1.23, -1.03) |
| Zambia | -0.30 (-0.34, -0.24) |  | -0.80 (-0.83, -0.77) |  | -1.14 (-1.17, -1.11) |  | -1.29 (-1.32, -1.25) |
| Zimbabwe | 1.52 (1.40, 1.65) |  | 1.43 (1.34, 1.55) |  | 1.32 (1.23, 1.42) |  | 1.54 (1.45, 1.65) |

**Table S3** The age-standardized incidence rate (ASIR) per 100 000 people for cervical cancer by age group and region in 1991, 2001, 2011 and 2021.

|  | 15-19 | 20-24 | 25-29 | 30-34 | 35-39 | 40-44 | 45-49 | 50-54 | 55-59 | 60-64 | 65-69 | 70-74 | 75-79 | 80-84 | 85-89 | 90-94 | 95+ |
| --- | --- | --- | --- | --- | --- | --- | --- | --- | --- | --- | --- | --- | --- | --- | --- | --- | --- |
| **Global** | | | | | | | | | | | | | | | | | |
| 1991 | 0.93 (0.83, 1.05) | 2.59 (2.33, 2.9) | 7.79 (7.22, 8.38) | 17.87 (16.78, 18.96) | 26.92 (25.1, 28.82) | 35.62 (33.2, 38.1) | 39.95 (37.26, 43.07) | 43.96 (40.44, 47.45) | 46.72 (43.36, 50.33) | 45.12 (42.05, 48.62) | 43.78 (41.02, 46.75) | 42.17 (38.98, 45.25) | 39.13 (36.52, 41.48) | 35.51 (31.42, 38.21) | 35.87 (30.5, 38.88) | 34.04 (27.91, 37.55) | 33.33 (25.52, 37.6) |
| 2001 | 0.96 (0.86, 1.06) | 2.78 (2.5, 3.08) | 7.65 (7.16, 8.13) | 15.82 (14.89, 16.66) | 24.82 (23.4, 26.14) | 32.66 (31.03, 34.55) | 34.73 (32.96, 36.45) | 37.98 (35.91, 39.87) | 42.07 (39.65, 44.53) | 39.82 (37.22, 42.4) | 39.67 (37.12, 42.1) | 37.6 (35.03, 39.81) | 34.43 (31.82, 36.39) | 32.4 (28.23, 35.44) | 33.21 (27.43, 36.42) | 31.28 (25.02, 34.64) | 29.35 (22.01, 33.42) |
| 2011 | 0.96 (0.85, 1.09) | 2.74 (2.48, 3.04) | 7.63 (7.09, 8.19) | 15.81 (14.72, 16.96) | 23.18 (21.64, 24.54) | 30.44 (28.62, 32.36) | 34.12 (32.08, 36.15) | 36.26 (34.14, 38.47) | 38.05 (35.79, 40.08) | 37.26 (34.82, 39.25) | 37.04 (34.36, 39.28) | 35.19 (32.39, 37.33) | 32.05 (28.76, 34.12) | 30.75 (26.57, 33.31) | 29.79 (24.45, 32.97) | 27.9 (21.72, 31.25) | 28.3 (20.9, 32.37) |
| 2021 | 0.92 (0.79, 1.12) | 2.75 (2.4, 3.19) | 7.58 (6.81, 8.32) | 16.12 (14.55, 17.79) | 24.13 (21.8, 26.46) | 31.75 (29.04, 34.82) | 34.15 (30.96, 37.6) | 36.61 (32.94, 40.78) | 39.94 (36.1, 44.21) | 37.31 (33.94, 40.52) | 36.01 (32.6, 39.45) | 33.59 (30.34, 36.67) | 30.45 (26.63, 33.22) | 28.21 (23.75, 31.06) | 26.73 (21.4, 29.87) | 26.03 (19.8, 29.44) | 26.01 (18.75, 30.21) |
| **High SDI** | | | | | | | | | | | | | | | | | |
| 1991 | 0.58 (0.55, 0.61) | 2.93 (2.79, 3.07) | 12.54 (12.05, 13.05) | 26.81 (25.83, 27.88) | 33.35 (32, 34.89) | 33.24 (32.04, 34.5) | 31.4 (30.3, 32.68) | 28.97 (27.89, 30.18) | 31 (29.9, 32.15) | 32.11 (30.66, 33.4) | 34.02 (32.13, 35.44) | 32.72 (30.74, 34.25) | 34.22 (31.34, 36.08) | 32.73 (28.16, 35.46) | 33.3 (26.96, 36.82) | 33.7 (26.7, 37.87) | 33.48 (24.89, 38.51) |
| 2001 | 0.45 (0.42, 0.48) | 2.16 (2.05, 2.26) | 8.6 (8.25, 8.91) | 19.04 (18.27, 19.83) | 26.08 (25.02, 27.2) | 28.09 (27.1, 29.31) | 27.01 (26, 27.99) | 25.01 (24.17, 25.83) | 25.5 (24.57, 26.39) | 23.62 (22.34, 24.61) | 25.04 (23.38, 26.23) | 25.81 (23.85, 27) | 26.5 (23.98, 28.18) | 26.32 (22.08, 28.81) | 30.2 (23.75, 33.48) | 28.56 (21.82, 32.38) | 26.53 (19.25, 30.7) |
| 2011 | 0.36 (0.34, 0.39) | 1.91 (1.82, 2.01) | 8.17 (7.85, 8.51) | 17.35 (16.61, 18.14) | 22.63 (21.52, 23.81) | 25.31 (24.2, 26.38) | 24.83 (23.76, 25.87) | 23.27 (22.39, 24.22) | 23.58 (22.68, 24.5) | 22.07 (20.7, 22.99) | 22.17 (20.55, 23.27) | 20.86 (18.74, 22.08) | 21.36 (18.75, 22.86) | 22.38 (17.94, 24.78) | 24.67 (18.69, 27.95) | 24.14 (17.69, 27.69) | 24.83 (17.69, 28.92) |
| 2021 | 0.3 (0.28, 0.33) | 1.54 (1.44, 1.65) | 7.13 (6.78, 7.53) | 16.08 (15.21, 16.99) | 20.31 (19.14, 21.39) | 22.01 (20.86, 23.2) | 21.9 (20.77, 23.01) | 21.2 (20.15, 22.31) | 21.71 (20.52, 22.81) | 20.41 (18.98, 21.55) | 20.3 (18.61, 21.6) | 19.28 (17.1, 20.62) | 19.2 (16.5, 20.86) | 18.95 (15.06, 21.47) | 20.12 (14.72, 23.18) | 23.72 (17.09, 27.65) | 23.95 (16.57, 28.26) |
| **High-middle SDI** | | | | | | | | | | | | | | | | | |
| 1991 | 0.52 (0.46, 0.6) | 1.61 (1.41, 1.9) | 5.31 (4.85, 5.93) | 13.21 (12.1, 14.54) | 20.25 (18.45, 22.43) | 25.92 (23.73, 28.26) | 27.14 (25.07, 29.81) | 29.69 (27.51, 32.47) | 32.07 (29.8, 35.02) | 33.37 (31.26, 35.96) | 36.05 (33.7, 38.41) | 35.69 (33.52, 38.51) | 36.29 (33.64, 38.62) | 30.93 (27.45, 33.4) | 25.86 (22.35, 28.31) | 27.05 (22.8, 29.88) | 29.56 (23.17, 33) |
| 2001 | 0.53 (0.47, 0.6) | 1.85 (1.66, 2.09) | 5.92 (5.44, 6.39) | 13.02 (12.07, 14.17) | 20.13 (18.61, 22.02) | 26.71 (24.86, 28.69) | 28.06 (26.12, 30.17) | 28.44 (26.44, 30.64) | 29.58 (27.57, 32.15) | 29.4 (27.67, 31.25) | 29.2 (27.51, 30.96) | 31.01 (29.06, 32.89) | 30.62 (28.31, 32.49) | 27.86 (24.39, 30.25) | 26.42 (22.63, 28.77) | 26.26 (21.96, 28.96) | 25.41 (20.11, 28.63) |
| 2011 | 0.54 (0.48, 0.61) | 1.9 (1.68, 2.13) | 6.81 (6.22, 7.39) | 16.02 (14.82, 17.52) | 22.45 (20.55, 24.53) | 28.11 (25.44, 31.17) | 31.25 (28.25, 34.53) | 31.23 (28.79, 33.72) | 32.5 (29.49, 35.18) | 30.97 (28.42, 33.41) | 29.68 (27.33, 32.08) | 29.42 (26.77, 31.39) | 28.44 (25.37, 30.52) | 27.9 (24.23, 30.33) | 25.8 (21.34, 28.77) | 25.65 (20.57, 28.8) | 27.74 (21.15, 31.68) |
| 2021 | 0.47 (0.4, 0.56) | 1.66 (1.39, 2) | 5.96 (5.07, 6.82) | 15.24 (13.09, 17.45) | 23.75 (20.24, 27.41) | 29.33 (25.34, 33.89) | 29.92 (25.14, 35.6) | 31.2 (25.69, 37.46) | 32.26 (26.85, 38.34) | 30.34 (26.4, 35.13) | 29.43 (25.54, 33.99) | 27.32 (23.61, 31.28) | 26.47 (22.2, 30.57) | 24.52 (20.16, 27.87) | 22.75 (17.99, 26.4) | 22.85 (17.72, 26.29) | 24.55 (17.91, 28.82) |
| **Middle SDI** | | | | | | | | | | | | | | | | | |
| 1991 | 0.88 (0.79, 0.99) | 2.21 (1.93, 2.49) | 6.17 (5.53, 6.89) | 15.32 (13.97, 16.64) | 24.12 (21.92, 26.52) | 34.19 (31.25, 37.29) | 38.71 (35.96, 41.74) | 45.24 (41.23, 49.71) | 49.17 (44.92, 53.85) | 46.51 (42.83, 50.7) | 45.68 (41.69, 49.6) | 45.24 (41.15, 50.03) | 43.06 (38.76, 47.05) | 42.13 (37.4, 45.51) | 52.63 (46.67, 57.36) | 45.97 (39.44, 51.24) | 39.75 (31, 46.24) |
| 2001 | 0.85 (0.77, 0.93) | 2.3 (2.04, 2.54) | 6.58 (5.94, 7.18) | 14.74 (13.53, 16.03) | 23.41 (21.7, 25.26) | 32.62 (30.37, 34.89) | 33.45 (31.3, 35.54) | 40.92 (37.73, 44.01) | 45.72 (42.32, 48.79) | 43.66 (40.36, 46.74) | 44.98 (41.8, 47.98) | 43.45 (40.22, 46.65) | 40.78 (37.17, 43.74) | 40.56 (35.81, 44.14) | 44.01 (37.02, 48.75) | 47.44 (39.33, 52.67) | 46.14 (36.22, 52.48) |
| 2011 | 0.85 (0.78, 0.94) | 2.23 (2.01, 2.45) | 6.43 (5.94, 6.95) | 14.32 (13.18, 15.39) | 21.94 (20.21, 23.58) | 29.97 (27.61, 32.32) | 34.65 (31.9, 37.36) | 37.52 (34.76, 40.19) | 40.6 (37.4, 43.79) | 40.44 (37.34, 43.39) | 40.91 (37.75, 43.89) | 40.14 (36.6, 43.23) | 38.27 (34.49, 41.43) | 38.17 (32.83, 41.64) | 38.65 (31.97, 42.95) | 37.46 (30.13, 41.7) | 40.17 (31.12, 45.56) |
| 2021 | 0.74 (0.65, 0.86) | 2.2 (1.91, 2.5) | 6.85 (6.13, 7.58) | 15.59 (13.84, 17.45) | 23.51 (20.79, 26.39) | 32.1 (28.93, 35.92) | 35.19 (31.25, 39.76) | 38.07 (33.05, 43.97) | 42.59 (36.82, 48.9) | 40.8 (36.26, 45.78) | 40.48 (35.44, 45.75) | 38.35 (33.23, 44.21) | 35.37 (30.03, 40.07) | 33.32 (27.89, 37.46) | 32.54 (25.69, 37.27) | 30.77 (23.86, 35.08) | 31.41 (23.1, 37.39) |
| **Low-middle SDI** | | | | | | | | | | | | | | | | | |
| 1991 | 1.18 (0.95, 1.42) | 3.02 (2.48, 3.69) | 7.73 (6.58, 9.01) | 16.82 (14.38, 19.27) | 28.05 (23.85, 32.3) | 43.33 (37.35, 50.15) | 51.72 (44.96, 59.54) | 62.29 (53.1, 71.59) | 65.64 (55.76, 75.69) | 63.93 (52.85, 74.65) | 56.38 (47.35, 65.46) | 53.38 (43.79, 63.02) | 43.45 (36.74, 51.03) | 38.53 (32.47, 45.09) | 39.21 (32.55, 47.17) | 33.26 (27.34, 39.3) | 30.61 (23.52, 36.67) |
| 2001 | 1.23 (1.02, 1.46) | 3.43 (2.89, 4.05) | 8.28 (7.19, 9.31) | 16.01 (14.17, 17.76) | 26.8 (23.46, 30.42) | 36.66 (32.82, 40.89) | 43.2 (39.06, 48.51) | 49.92 (44.7, 55.19) | 55.68 (49.39, 62.24) | 53.24 (46.47, 60.48) | 53.22 (46.63, 60.45) | 49 (42.12, 56.33) | 42.43 (37.65, 48.14) | 38 (32.98, 44.47) | 37.94 (31.36, 44.86) | 31.02 (24.69, 37.26) | 29.88 (21.54, 36.2) |
| 2011 | 1.18 (1, 1.42) | 3.42 (2.86, 4.04) | 7.98 (7.08, 9.01) | 15.3 (13.71, 17.28) | 23.03 (20.6, 26.11) | 32.58 (29.39, 36.46) | 38.04 (34.59, 42.12) | 43.94 (39.68, 47.88) | 46.91 (42.43, 52.03) | 49.1 (44.42, 54.45) | 47.35 (42.11, 52.64) | 45.64 (40.6, 50.84) | 39.58 (35.28, 43.98) | 38.71 (34.09, 43.77) | 36.87 (30.52, 42.2) | 30.63 (24.26, 36.28) | 29.26 (20.91, 34.82) |
| 2021 | 1.07 (0.86, 1.42) | 3.15 (2.56, 3.94) | 7.7 (6.57, 8.86) | 15.67 (13.39, 18.43) | 24.26 (20.92, 28.08) | 34.24 (29.97, 38.76) | 37.9 (33.38, 43.02) | 43.67 (38.27, 49.25) | 50.98 (44.3, 58.15) | 46.96 (41.53, 52.57) | 46.11 (40.93, 51.45) | 46.11 (40.51, 51.94) | 38.44 (33.84, 42.8) | 38.14 (32.64, 43.33) | 37.4 (31.48, 42.68) | 31.65 (25.26, 36.42) | 29.32 (20.58, 34.97) |
| **Low SDI** | | | | | | | | | | | | | | | | | |
| 1991 | 1.71 (1.34, 2.17) | 4.63 (3.59, 5.94) | 11.89 (9.63, 14.59) | 22.32 (18.02, 27.46) | 40.2 (32.4, 48.86) | 59.82 (48.78, 72.65) | 76.98 (64.02, 93.11) | 95.71 (79.32, 115.66) | 104.86 (87.56, 129.25) | 106.64 (87.28, 133.14) | 99.08 (81.58, 122.04) | 91.37 (74.59, 112.87) | 71.21 (58.37, 88.95) | 65.16 (52.27, 80.55) | 64.65 (50.45, 81.39) | 44.33 (33.1, 58.58) | 34.1 (24.47, 45.84) |
| 2001 | 1.72 (1.39, 2.15) | 4.81 (3.92, 6.16) | 11.91 (9.93, 14.27) | 20.82 (17.35, 25.03) | 36.34 (30.11, 43.13) | 52.19 (43.44, 62.53) | 66.85 (55.39, 79.23) | 78.77 (67.02, 93.02) | 91.16 (78.37, 109.06) | 92.73 (78.07, 110.24) | 91.13 (76.88, 110.28) | 86.6 (72.99, 103.9) | 69.7 (57.9, 83.34) | 62.62 (51.58, 77.66) | 59.18 (47.02, 76.03) | 39.33 (30.5, 52.81) | 31.97 (22.98, 45.76) |
| 2011 | 1.56 (1.25, 1.93) | 4.59 (3.68, 5.72) | 10.82 (8.93, 13.13) | 19.05 (15.18, 23.21) | 30.21 (24.73, 37.14) | 43.47 (36.49, 53.07) | 54.04 (45.57, 66.18) | 67.42 (56.68, 81.45) | 74.53 (62.19, 87.67) | 77.64 (65.43, 91.94) | 77.8 (66.18, 90.26) | 78.96 (66.6, 92.52) | 63.24 (51.97, 76.11) | 61.75 (50.75, 76.02) | 59.07 (47.25, 72.89) | 41.51 (31.31, 54.39) | 33.27 (23.94, 44.93) |
| 2021 | 1.52 (1.18, 1.91) | 4.47 (3.52, 5.53) | 10.69 (8.63, 12.75) | 19.65 (15.49, 24.32) | 30.71 (24.59, 37.64) | 43.04 (35.13, 52.7) | 52.12 (42.9, 63.86) | 62.05 (51.62, 76.12) | 74.57 (60.61, 89.48) | 76.1 (62.73, 90.9) | 73.6 (62.21, 86.99) | 73.81 (62.66, 87.39) | 60.78 (50.45, 72.42) | 61.76 (50.83, 74.18) | 61.92 (48.83, 74.46) | 48.22 (37.04, 59.58) | 39.24 (26.76, 51.13) |

**Table S4** The age-standardized mortality rate (ASMR) per 100 000 people for cervical cancer by age group and region in 1991, 2001, 2011 and 2021.

|  | 15-19 | 20-24 | 25-29 | 30-34 | 35-39 | 40-44 | 45-49 | 50-54 | 55-59 | 60-64 | 65-69 | 70-74 | 75-79 | 80-84 | 85-89 | 90-94 | 95+ |
| --- | --- | --- | --- | --- | --- | --- | --- | --- | --- | --- | --- | --- | --- | --- | --- | --- | --- |
| **Global** | | |  |  |  |  |  |  |  |  |  |  |  |  |  |  |  |
| 1991 | 0.27 (0.24, 0.31) | 0.72 (0.63, 0.83) | 1.75 (1.58, 1.94) | 4.02 (3.66, 4.39) | 7.64 (6.99, 8.3) | 13.18 (12.03, 14.46) | 18.68 (17.13, 20.43) | 24.5 (22.2, 26.87) | 28.86 (26.5, 31.53) | 29.59 (27.15, 32.15) | 31.46 (29.31, 33.99) | 34.88 (32.19, 37.77) | 35.42 (32.88, 37.62) | 36.78 (32.75, 39.6) | 41.65 (35.87, 45.18) | 43.17 (35.71, 47.53) | 45.62 (35.08, 51.34) |
| 2001 | 0.26 (0.24, 0.3) | 0.76 (0.67, 0.86) | 1.72 (1.58, 1.88) | 3.49 (3.22, 3.74) | 6.69 (6.18, 7.15) | 11.24 (10.53, 12.04) | 14.84 (13.91, 15.83) | 19.56 (18.37, 20.7) | 24.69 (23.06, 26.33) | 25.37 (23.59, 27.15) | 28.24 (26.22, 30.23) | 30.53 (28.41, 32.49) | 30.76 (28.37, 32.59) | 33.21 (29.28, 36.45) | 37.64 (31.47, 41.34) | 38.94 (31.43, 42.97) | 39.5 (29.81, 44.82) |
| 2011 | 0.24 (0.21, 0.27) | 0.66 (0.58, 0.74) | 1.49 (1.36, 1.64) | 3.09 (2.83, 3.38) | 5.54 (5.08, 6) | 9.24 (8.64, 9.93) | 12.88 (12.07, 13.75) | 17.05 (15.92, 18.23) | 20.03 (18.68, 21.25) | 21.57 (20.12, 22.87) | 24.63 (22.83, 26.2) | 27.43 (25.43, 29.17) | 27.77 (25.17, 29.57) | 30.72 (26.89, 33.24) | 33.29 (27.62, 36.58) | 33.9 (26.7, 37.82) | 37.06 (27.42, 42.22) |
| 2021 | 0.21 (0.18, 0.26) | 0.62 (0.54, 0.74) | 1.4 (1.23, 1.56) | 2.86 (2.54, 3.22) | 5.33 (4.8, 5.95) | 9.19 (8.33, 10.19) | 12.27 (11.1, 13.59) | 15.9 (14.34, 17.61) | 19.81 (17.87, 21.75) | 20.52 (18.81, 22.36) | 22.45 (20.44, 24.51) | 24.91 (22.62, 27.14) | 25.61 (22.74, 27.94) | 27.95 (23.76, 30.69) | 30.19 (24.47, 33.77) | 31.28 (24.03, 35.23) | 33.88 (24.38, 39.28) |
| **High SDI** | |  |  |  |  |  |  |  |  |  |  |  |  |  |  |  |  |
| 1991 | 0.05 (0.05, 0.06) | 0.23 (0.22, 0.25) | 0.9 (0.86, 0.93) | 2.28 (2.2, 2.37) | 4.11 (3.95, 4.28) | 5.92 (5.74, 6.1) | 7.71 (7.46, 7.99) | 9.69 (9.31, 10.09) | 12.31 (11.91, 12.75) | 14.23 (13.63, 14.73) | 17.76 (16.91, 18.42) | 20.5 (19.3, 21.38) | 25.33 (23.22, 26.64) | 29.34 (25.6, 31.63) | 34.45 (28.07, 37.98) | 39.97 (31.78, 44.87) | 43.26 (32.45, 49.39) |
| 2001 | 0.04 (0.04, 0.04) | 0.17 (0.16, 0.18) | 0.62 (0.6, 0.64) | 1.58 (1.53, 1.63) | 3.06 (2.96, 3.18) | 4.84 (4.69, 5.02) | 6.48 (6.3, 6.68) | 7.95 (7.73, 8.2) | 9.36 (9.07, 9.65) | 9.86 (9.4, 10.27) | 12.32 (11.61, 12.89) | 15.76 (14.67, 16.45) | 18.83 (17.24, 19.92) | 22.39 (19.12, 24.43) | 29.63 (23.46, 32.73) | 32.68 (25.12, 36.95) | 33.18 (24.17, 38.18) |
| 2011 | 0.03 (0.03, 0.03) | 0.14 (0.13, 0.15) | 0.54 (0.52, 0.56) | 1.33 (1.3, 1.38) | 2.42 (2.34, 2.5) | 3.86 (3.74, 3.98) | 5.27 (5.08, 5.44) | 6.75 (6.54, 7) | 8.14 (7.86, 8.42) | 8.47 (7.99, 8.77) | 9.88 (9.2, 10.34) | 11.72 (10.7, 12.37) | 14.26 (12.67, 15.21) | 18.33 (14.99, 20.25) | 23.7 (18.18, 26.75) | 26.49 (19.6, 30.19) | 30.15 (21.55, 35.07) |
| 2021 | 0.02 (0.02, 0.02) | 0.11 (0.1, 0.11) | 0.44 (0.42, 0.46) | 1.14 (1.09, 1.2) | 2.01 (1.92, 2.11) | 3.14 (3.01, 3.29) | 4.34 (4.17, 4.53) | 5.69 (5.45, 5.94) | 6.96 (6.62, 7.3) | 7.46 (7.01, 7.86) | 8.86 (8.15, 9.42) | 10.47 (9.41, 11.15) | 12.27 (10.67, 13.24) | 15.2 (12.08, 17.07) | 19.57 (14.47, 22.38) | 25.85 (18.84, 29.96) | 29.19 (20.05, 34.51) |
| **High-middle SDI** | | |  |  |  |  |  |  |  |  |  |  |  |  |  |  |  |
| 1991 | 0.12 (0.11, 0.14) | 0.37 (0.32, 0.44) | 1.07 (0.96, 1.21) | 2.75 (2.51, 3.05) | 5.23 (4.74, 5.77) | 8.53 (7.77, 9.44) | 11.14 (10.19, 12.27) | 14.62 (13.51, 16.02) | 17.86 (16.52, 19.68) | 19.98 (18.72, 21.54) | 24.61 (23.07, 26.21) | 28.92 (27.15, 31.38) | 33.22 (30.98, 35.33) | 33.02 (29.65, 35.78) | 31.49 (27.45, 34.42) | 35.57 (30.03, 39.28) | 42.18 (33.14, 47.1) |
| 2001 | 0.1 (0.09, 0.12) | 0.37 (0.33, 0.41) | 1.05 (0.97, 1.13) | 2.42 (2.25, 2.63) | 4.64 (4.28, 5.03) | 7.93 (7.4, 8.47) | 10.51 (9.76, 11.29) | 13.16 (12.24, 14.12) | 15.56 (14.44, 16.98) | 16.95 (15.98, 17.99) | 19.35 (18.22, 20.46) | 24.42 (22.92, 25.93) | 27.42 (25.41, 29.04) | 29.2 (25.9, 31.76) | 31.46 (27.21, 34.23) | 33.79 (28.51, 37.25) | 35.12 (28.27, 39.4) |
| 2011 | 0.08 (0.07, 0.09) | 0.28 (0.25, 0.31) | 0.93 (0.86, 0.99) | 2.32 (2.17, 2.5) | 4.05 (3.74, 4.37) | 6.52 (5.95, 7.13) | 9.33 (8.49, 10.22) | 12 (11.13, 12.88) | 14.5 (13.26, 15.61) | 15.31 (14.21, 16.45) | 17.26 (15.9, 18.62) | 20.98 (19.31, 22.33) | 23.27 (20.94, 25.04) | 27.12 (23.72, 29.35) | 28.99 (24.31, 32.1) | 31.18 (25.13, 34.82) | 35.68 (27.53, 40.57) |
| 2021 | 0.06 (0.05, 0.07) | 0.21 (0.18, 0.25) | 0.7 (0.61, 0.79) | 1.89 (1.65, 2.13) | 3.74 (3.26, 4.26) | 6.08 (5.35, 6.88) | 7.97 (6.77, 9.26) | 10.54 (8.76, 12.48) | 12.88 (10.89, 15.05) | 13.8 (12.14, 15.92) | 15.95 (13.9, 18.38) | 18.37 (15.85, 21.02) | 20.75 (17.41, 24.03) | 23.3 (19.45, 26.35) | 25.28 (20.23, 29.18) | 27.3 (21.28, 31.34) | 31.82 (23.25, 37.29) |
| **Middle SDI** | | |  |  |  |  |  |  |  |  |  |  |  |  |  |  |  |
| 1991 | 0.24 (0.21, 0.26) | 0.61 (0.53, 0.69) | 1.53 (1.36, 1.72) | 3.89 (3.53, 4.25) | 7.4 (6.67, 8.17) | 13.16 (12.04, 14.44) | 18.14 (16.69, 19.58) | 25.07 (22.71, 27.43) | 30.51 (27.79, 33.61) | 31.27 (28.62, 34.06) | 34.7 (31.52, 37.89) | 39.93 (36.23, 44.41) | 42.6 (38.41, 46.67) | 47.61 (42.48, 51.74) | 66.08 (58.53, 72.22) | 63.22 (54.08, 70.21) | 59.88 (47.07, 69.64) |
| 2001 | 0.2 (0.18, 0.22) | 0.55 (0.49, 0.61) | 1.4 (1.26, 1.54) | 3.22 (2.96, 3.5) | 6.24 (5.79, 6.72) | 10.97 (10.25, 11.67) | 13.85 (12.92, 14.74) | 20.5 (18.86, 21.95) | 25.97 (24.01, 27.55) | 27.16 (25.25, 29.14) | 31.99 (29.73, 34.16) | 36.36 (33.55, 38.99) | 38.64 (35.22, 41.63) | 44.66 (39.62, 48.56) | 54.64 (46.18, 60.47) | 63.84 (53.05, 70.69) | 67.9 (53.14, 77.29) |
| 2011 | 0.17 (0.15, 0.19) | 0.45 (0.4, 0.49) | 1.13 (1.04, 1.21) | 2.62 (2.44, 2.81) | 4.97 (4.62, 5.33) | 8.65 (8.08, 9.27) | 12.5 (11.6, 13.37) | 16.95 (15.79, 18.1) | 20.51 (18.97, 22.1) | 22.55 (20.88, 23.97) | 26.62 (24.63, 28.43) | 31.51 (28.9, 33.91) | 34.41 (31.16, 37.05) | 40.63 (35.14, 44.21) | 47.1 (39.4, 52.17) | 49.66 (39.93, 55.3) | 57.84 (44.61, 65.53) |
| 2021 | 0.13 (0.11, 0.15) | 0.38 (0.33, 0.43) | 1.04 (0.94, 1.16) | 2.44 (2.19, 2.7) | 4.66 (4.2, 5.17) | 8.37 (7.61, 9.24) | 11.54 (10.36, 12.86) | 15.12 (13.38, 17.2) | 19.59 (17.17, 22.16) | 21.12 (18.88, 23.39) | 24.18 (21.51, 27.22) | 27.91 (24.49, 31.62) | 30.08 (25.75, 33.76) | 34.22 (29.14, 38.38) | 38.77 (30.95, 44.22) | 39.98 (30.94, 45.67) | 44.38 (32.7, 52.49) |
| **Low-middle SDI** | | |  |  |  |  |  |  |  |  |  |  |  |  |  |  |  |
| 1991 | 0.4 (0.32, 0.48) | 1.06 (0.87, 1.32) | 2.42 (2.06, 2.84) | 5.43 (4.62, 6.33) | 10.73 (9.06, 12.45) | 20.58 (17.63, 24.09) | 29.32 (25.37, 33.87) | 40.72 (34.51, 47.01) | 47.18 (40.29, 54.76) | 49.33 (40.8, 57.71) | 47.8 (39.9, 55.81) | 50.81 (41.5, 60.11) | 45.3 (38.39, 53.15) | 45.19 (38.17, 52.88) | 50.64 (42.24, 60.64) | 46.49 (38.31, 55.25) | 46.53 (35.79, 55.78) |
| 2001 | 0.38 (0.32, 0.46) | 1.11 (0.93, 1.31) | 2.37 (2.06, 2.71) | 4.7 (4.11, 5.26) | 9.45 (8.16, 10.84) | 16.03 (14.22, 18.1) | 22.81 (20.35, 25.63) | 30.83 (27.39, 34.26) | 38.23 (33.85, 43) | 39.43 (34.19, 44.89) | 43.76 (38.19, 49.9) | 45.38 (38.78, 52.41) | 43.33 (38.44, 49.51) | 43.8 (37.95, 51.71) | 48.43 (40.28, 57.26) | 42.88 (34.27, 51.51) | 44.65 (32.36, 54.28) |
| 2011 | 0.32 (0.27, 0.39) | 0.97 (0.81, 1.15) | 2.01 (1.78, 2.27) | 3.93 (3.47, 4.45) | 7.18 (6.4, 8.22) | 12.88 (11.64, 14.41) | 18.48 (16.78, 20.53) | 25.36 (22.7, 27.72) | 30.34 (27.23, 33.76) | 34.7 (31.17, 38.32) | 37.44 (33.24, 41.88) | 41.28 (36.52, 46.32) | 39.7 (35.26, 44.07) | 44.15 (38.84, 50.04) | 46.77 (39.41, 53.61) | 42.03 (33.54, 49.87) | 43.25 (31.14, 51.51) |
| 2021 | 0.26 (0.2, 0.34) | 0.78 (0.64, 0.97) | 1.7 (1.45, 1.97) | 3.56 (3.04, 4.25) | 6.75 (5.84, 7.79) | 12.25 (10.72, 13.98) | 16.87 (14.86, 19.26) | 23.5 (20.51, 26.65) | 31.01 (26.78, 35.45) | 31.17 (27.59, 35.02) | 34.7 (30.8, 38.75) | 40.25 (35.25, 45.46) | 37.53 (33.04, 41.83) | 42.82 (36.82, 48.96) | 47.05 (39.5, 53.57) | 43.01 (34.53, 49.48) | 42.99 (30.28, 51.15) |
| **Low SDI** | | |  |  |  |  |  |  |  |  |  |  |  |  |  |  |  |
| 1991 | 0.66 (0.52, 0.84) | 1.85 (1.45, 2.39) | 4.31 (3.52, 5.37) | 8.42 (6.79, 10.32) | 17.65 (14.37, 21.52) | 31.7 (26.02, 38.3) | 47.89 (39.73, 58.38) | 67.5 (56.11, 82.09) | 80.48 (66.95, 99.36) | 87.23 (71.33, 108.72) | 88.27 (72.97, 108.45) | 90.22 (73.53, 112.54) | 76.75 (62.72, 96.45) | 78.05 (62.99, 96.73) | 84.81 (66.76, 106.82) | 63.22 (47.32, 83.46) | 53.15 (38.2, 72.28) |
| 2001 | 0.62 (0.51, 0.78) | 1.81 (1.47, 2.3) | 4.07 (3.43, 4.92) | 7.44 (6.18, 9.06) | 15.24 (12.59, 18.12) | 26.77 (22.47, 32) | 40.52 (33.8, 48.13) | 54.49 (46.1, 64.69) | 68.92 (59.15, 82.67) | 74.84 (63.29, 89.14) | 80.34 (68.05, 97.21) | 84.81 (71.77, 101.49) | 74.69 (62.26, 89.1) | 74.78 (61.73, 93.17) | 77.47 (61.82, 99.98) | 55.95 (43.6, 74.72) | 49.72 (36.04, 70.86) |
| 2011 | 0.5 (0.41, 0.62) | 1.53 (1.24, 1.93) | 3.31 (2.75, 4.04) | 6.15 (4.93, 7.46) | 11.65 (9.6, 14.46) | 20.78 (17.35, 25.23) | 30.99 (26.12, 37.71) | 44.77 (37.78, 53.97) | 54.48 (45.38, 64.35) | 60.83 (51.17, 72.25) | 67.03 (57.1, 77.81) | 76.04 (64.28, 89.15) | 66.95 (54.96, 81.44) | 73.2 (60.55, 90.73) | 77 (62.06, 95.24) | 58.84 (44.41, 77.4) | 51.52 (37.4, 69.71) |
| 2021 | 0.44 (0.34, 0.56) | 1.35 (1.07, 1.68) | 2.94 (2.38, 3.54) | 5.67 (4.54, 7.09) | 10.71 (8.62, 13.33) | 18.91 (15.36, 22.99) | 27.9 (23.02, 34.38) | 38.93 (32.22, 47.36) | 51.76 (42.08, 61.89) | 57.07 (47.24, 68.17) | 61.15 (51.65, 72.28) | 69.12 (58.43, 81.38) | 63.05 (52.38, 75.19) | 72.2 (59.69, 86.78) | 80.08 (63.39, 96.66) | 67.84 (52.25, 83.57) | 60.39 (41.74, 79) |

**Table S5** The age-standardized DALY rate (ASDR) per 100 000 people for cervical cancer by age group and region in 1991, 2001, 2011 and 2021.

|  | 15-19 | 20-24 | 25-29 | 30-34 | 35-39 | 40-44 | 45-49 | 50-54 | 55-59 | 60-64 | 65-69 | 70-74 | 75-79 | 80-84 | 85-89 | 90-94 | 95+ |
| --- | --- | --- | --- | --- | --- | --- | --- | --- | --- | --- | --- | --- | --- | --- | --- | --- | --- |
| **Global** | | | | | | | | | | | | | | | | | |
| 1991 | 20.29 (17.63, 23.23) | 49.9 (43.86, 57.45) | 113.51 (102.3, 126.2) | 241.05 (219.9, 263.04) | 416.15 (381.33, 452.41) | 647.04 (589.59, 709.42) | 818.91 (750.68, 893.09) | 952.16 (861.95, 1042.78) | 983.34 (903.04, 1073.73) | 869.26 (798.04, 944.23) | 778.41 (725.25, 842.3) | 709.45 (655.09, 767.57) | 574.21 (533.38, 610.37) | 467.54 (415.73, 503.58) | 420.91 (362.31, 456.95) | 378.89 (313.84, 417.21) | 376.75 (289.36, 423.45) |
| 2001 | 19.62 (17.57, 22.25) | 52.6 (46.45, 59.6) | 111.86 (102, 121.77) | 209.43 (192.98, 225.35) | 365.27 (337.36, 391.15) | 552.79 (519.07, 590.46) | 652.96 (612.18, 697.35) | 763.05 (714.13, 809.01) | 842.65 (788.39, 900.75) | 745.18 (692.4, 797.04) | 698.4 (648.67, 745.79) | 620.4 (577.07, 659.1) | 500.39 (462.07, 529.6) | 423.09 (372.74, 463.5) | 378.91 (316.59, 415.48) | 341.74 (275.65, 377.04) | 323.64 (245.58, 366.04) |
| 2011 | 17.75 (15.52, 20.4) | 45.64 (40.31, 51.68) | 97.52 (88.33, 107.29) | 186.63 (171.12, 204.88) | 303.69 (279.6, 329.92) | 456.58 (425.65, 491.21) | 569.14 (532.76, 609.28) | 666.9 (623.41, 714.29) | 686.01 (640.08, 728.98) | 636.62 (594.43, 674.43) | 610.05 (565.66, 648.71) | 557.49 (517.97, 594.56) | 451.27 (408.75, 480.93) | 390.5 (340.87, 423.02) | 335.95 (278.63, 368.87) | 297.89 (234.7, 332.4) | 302.78 (225.17, 345.77) |
| 2021 | 15.81 (13.25, 19.27) | 43.55 (37.39, 51.41) | 91.29 (80.46, 101.69) | 173.48 (153.87, 195.42) | 293.67 (264.29, 325.56) | 454.9 (412.22, 502.66) | 543.09 (488.42, 602.13) | 623.13 (562.01, 689.12) | 680.46 (615.02, 746.81) | 606.6 (556.57, 659.74) | 557.84 (507.57, 608.2) | 508.71 (460.87, 552.76) | 416.86 (370.68, 454.66) | 355.28 (302.64, 389.83) | 304.61 (246.56, 340.06) | 274.83 (210.45, 310.34) | 278.16 (200.78, 322.5) |
| **High SDI** | | | | | | | | | | | | | | | | | |
| 1991 | 4.1 (3.81, 4.42) | 17.27 (16.26, 18.3) | 62.61 (59.57, 65.82) | 145.29 (138.96, 151.69) | 233.67 (223.14, 246.33) | 299.57 (289.45, 309.26) | 346.39 (333.73, 359.93) | 383.84 (368.73, 400.98) | 425.95 (411.61, 440.83) | 423.38 (405.5, 439.32) | 443.6 (422.84, 460.67) | 421.82 (396.66, 440.84) | 410.92 (378.03, 432.7) | 373.78 (326.73, 403.08) | 348.36 (282.69, 384.1) | 351.03 (280.16, 394.49) | 357.97 (268.33, 409.05) |
| 2001 | 3.1 (2.91, 3.34) | 12.76 (12.09, 13.46) | 43.35 (41.46, 45.24) | 100.97 (96.93, 106.13) | 174.62 (167.29, 182.77) | 245.56 (236.98, 256.2) | 292.01 (282.9, 301.38) | 315.94 (306.72, 326.36) | 325.58 (314.3, 336.08) | 294.73 (280.47, 306.69) | 308.9 (291.08, 323.12) | 323.11 (300.04, 338.14) | 307.68 (282.03, 326.33) | 286.64 (244.69, 312.29) | 298.03 (236.6, 330.14) | 287.17 (220.96, 325) | 272.42 (199.4, 313.09) |
| 2011 | 2.18 (2.03, 2.37) | 10.41 (9.84, 10.98) | 38.17 (36.43, 40.26) | 86.21 (82.29, 90.52) | 139.4 (133.39, 145.75) | 197.6 (190.03, 205.62) | 238.81 (229.75, 248.04) | 269.39 (259.81, 279.3) | 284.28 (274.93, 294.33) | 254.38 (240.16, 263.52) | 248.97 (231.49, 260.29) | 241.62 (220.29, 255.25) | 233.62 (206.72, 250.4) | 233.69 (191.25, 257.5) | 239.21 (183.72, 269.2) | 233.43 (172.52, 266.32) | 247.11 (177.1, 286.58) |
| 2021 | 1.68 (1.55, 1.85) | 8.01 (7.48, 8.59) | 31.21 (29.49, 33.09) | 74.54 (70.52, 79.33) | 117.09 (110.41, 124.3) | 161.88 (154.6, 169.86) | 197.85 (189.1, 207.38) | 228.28 (217.96, 238.59) | 243.81 (231.8, 255.99) | 224.75 (211.03, 236.38) | 224.06 (206.51, 237.21) | 216.26 (194.27, 230.42) | 201.56 (174.49, 217.34) | 194.3 (154.76, 218.29) | 197.38 (146.03, 225.65) | 227.59 (164.77, 263.84) | 238.52 (163.82, 282.52) |
| **High-middle SDI** | | | | | | | | | | | | | | | | | |
| 1991 | 9.01 (7.87, 10.39) | 25.79 (22.32, 30.82) | 69.45 (63.02, 78.16) | 165.1 (151.08, 183.2) | 285.83 (258.77, 316.05) | 420.8 (383.16, 465.53) | 490.15 (448, 538.13) | 569.72 (526.01, 624.42) | 610.24 (565.06, 671.89) | 587.48 (550.86, 634.34) | 610.26 (570.43, 649.52) | 588 (551.02, 636.89) | 536.89 (500.88, 571.1) | 419.99 (376.44, 454.93) | 318.33 (278.03, 346.81) | 312.21 (263.75, 344.64) | 351.14 (276.41, 390.71) |
| 2001 | 7.87 (6.98, 8.85) | 25.74 (23.16, 28.61) | 68.86 (63, 74.21) | 146.14 (134.65, 159.2) | 254.58 (235.07, 276.22) | 391.89 (364.17, 420.61) | 464.65 (431.29, 499.35) | 515.93 (480.44, 553.69) | 532.75 (494.2, 580.72) | 499 (469.16, 530.64) | 478.86 (449.28, 505.98) | 495.37 (465.34, 525.93) | 446.62 (413.46, 473.49) | 371.96 (330.72, 405.23) | 316.54 (273.6, 343.93) | 296.62 (250.31, 326.7) | 291.07 (234.11, 325.95) |
| 2011 | 6.12 (5.48, 6.79) | 20.1 (17.95, 22.49) | 61.57 (56.48, 66.28) | 142.1 (132.69, 153.74) | 225.11 (206.29, 243.76) | 325.61 (296.52, 355.75) | 416.31 (376.59, 457.38) | 472.7 (437.67, 507.27) | 499.44 (457.1, 537.56) | 455.17 (422.67, 487.62) | 429.02 (394.81, 462) | 425.99 (392.13, 452.94) | 377.83 (339.56, 405.98) | 344.55 (301.51, 373.3) | 292.78 (244.87, 324.43) | 274.01 (220.73, 306.41) | 294.99 (227.21, 335.97) |
| 2021 | 4.65 (3.95, 5.45) | 15.38 (13.19, 18.13) | 46.68 (40.91, 52.62) | 116.63 (101.93, 132.14) | 209.93 (183.89, 239.32) | 306.05 (268.61, 346.25) | 357.15 (304.89, 415.54) | 417.43 (347.75, 493.57) | 446.03 (379.48, 522.4) | 410.62 (361.23, 472.05) | 398.1 (348.26, 459.07) | 376.2 (325.25, 431.34) | 337.59 (284.85, 389.12) | 295.6 (247.41, 333.09) | 254.91 (204.26, 293.47) | 240.07 (186.95, 276.15) | 263.9 (192.79, 308.1) |
| **Middle SDI** | | | | | | | | | | | | | | | | | |
| 1991 | 17.63 (15.65, 19.58) | 42.28 (37.08, 48.26) | 98.73 (87.85, 111.29) | 232.02 (209.57, 253.56) | 401.82 (363.73, 442.43) | 645.21 (587.7, 708.65) | 794.99 (733.82, 857.49) | 973.86 (884.17, 1065.35) | 1039.2 (945.99, 1143.68) | 917.7 (839.78, 997.04) | 856.47 (776.36, 933.17) | 809.47 (735.44, 897.71) | 691.11 (622.8, 755.85) | 602.88 (539.01, 655.73) | 666.91 (589.6, 728.5) | 554.53 (473.12, 616.82) | 490.96 (387.1, 570.51) |
| 2001 | 14.75 (13.47, 16.19) | 38.59 (34.3, 42.45) | 91.11 (81.79, 99.92) | 193.21 (177.44, 209.61) | 340.94 (315.9, 367.93) | 540.34 (503.44, 574.12) | 609.59 (567.99, 647.33) | 799.71 (736.69, 855.39) | 887.51 (818.69, 943.33) | 797.64 (740.39, 855) | 790.7 (735.84, 842.29) | 738.34 (682.19, 791.38) | 627.34 (571.61, 674.74) | 566.89 (503.18, 618.06) | 550.31 (464.11, 608.3) | 559.22 (466.09, 620.03) | 556.87 (436.51, 633.14) |
| 2011 | 12.89 (11.62, 14.36) | 31.29 (28.12, 34.41) | 73.98 (68.35, 80.18) | 158.62 (147.03, 170.17) | 273.16 (253.68, 293.32) | 427.88 (398.91, 460.16) | 553.27 (512.93, 591.09) | 663.61 (618.02, 706.4) | 703.34 (651.98, 761.45) | 665.92 (618.79, 710.64) | 659.54 (608.5, 703.35) | 639.74 (586.46, 687.64) | 557.81 (505.89, 601.27) | 515.55 (446.49, 559.98) | 474.78 (398.88, 526.16) | 435.26 (350.85, 484.5) | 472.32 (364.57, 534.83) |
| 2021 | 9.65 (8.4, 11.47) | 27 (23.46, 30.35) | 68.89 (62.12, 75.92) | 149.01 (133.3, 164.68) | 258.16 (232.64, 286.61) | 415.74 (375.92, 459.74) | 511.98 (459.77, 571.42) | 594.25 (522.35, 675.53) | 674.77 (593.7, 761.68) | 625.49 (560.75, 693.39) | 601.4 (533.41, 675.81) | 569.77 (500.78, 643.75) | 488.82 (419.18, 548.81) | 434.32 (370.38, 487.61) | 390.79 (311.87, 444.97) | 350.65 (272.04, 401.6) | 364.79 (269.31, 431.26) |
| **Low-middle SDI** | | | | | | | | | | | | | | | | | |
| 1991 | 29.47 (23.27, 35.25) | 72.86 (59.79, 91.03) | 155.78 (132.55, 182.22) | 322.09 (273.55, 373.77) | 578.99 (489.2, 673.44) | 1002.67 (859.43, 1174.26) | 1278.7 (1106.99, 1476.64) | 1576.62 (1336.1, 1818.6) | 1601.26 (1366.99, 1855.93) | 1444.05 (1197.46, 1687.2) | 1177.93 (982.83, 1373.53) | 1030.26 (841.98, 1216.55) | 734.99 (622.85, 862.38) | 574.3 (485.08, 672.28) | 511.67 (426.63, 613.21) | 407.37 (334.59, 483.17) | 376.93 (290.09, 452.23) |
| 2001 | 28.27 (23.36, 34.1) | 76.36 (63.95, 90.71) | 152.91 (133.28, 173.76) | 279.24 (243.91, 313.05) | 511.46 (441.91, 586.02) | 783.39 (694.37, 885.24) | 997.26 (887.55, 1117.96) | 1196.14 (1061.92, 1325.89) | 1298.87 (1150.12, 1459.64) | 1152.79 (1001.25, 1309.79) | 1079.01 (940.72, 1229.65) | 921.11 (787.01, 1064.48) | 703.8 (623.75, 804.36) | 557.3 (481.88, 658.52) | 488.05 (405.14, 578.17) | 375.73 (299.81, 451.53) | 352.59 (257.64, 427.22) |
| 2011 | 23.88 (20.02, 28.93) | 67.09 (56.42, 80.1) | 129.68 (115.05, 146.84) | 234.71 (207.43, 265.83) | 389.93 (347.12, 447.65) | 630.79 (569.22, 703.05) | 809.32 (734.74, 898.14) | 985.86 (883.08, 1079.21) | 1031.83 (924.48, 1148.09) | 1016.91 (912.1, 1123.81) | 923.17 (818.77, 1034.83) | 836.55 (740.68, 939.44) | 644.23 (571.8, 714.31) | 561.61 (493.86, 637.06) | 472.09 (397, 542.14) | 368.35 (293.74, 437.21) | 340.84 (247.18, 404.4) |
| 2021 | 19.07 (15.19, 25.08) | 54.4 (44.32, 67.45) | 110.49 (94.31, 128.32) | 213.5 (181.6, 254.39) | 368.08 (317.7, 424.1) | 601.74 (527.49, 686.99) | 741.11 (651.91, 846.88) | 914.44 (798.63, 1037.77) | 1057.86 (912.96, 1210.76) | 916.2 (809.99, 1026.74) | 856.66 (759.25, 956.08) | 818.63 (716.43, 923.55) | 609.43 (536.22, 679.29) | 544.39 (467.25, 622.95) | 475.61 (399.11, 542.66) | 376.96 (302.68, 432.72) | 350.15 (247.33, 415.06) |
| **Low SDI** | | | | | | | | | | | | | | | | | |
| 1991 | 48.4 (38.66, 61.88) | 127.04 (99.9, 164.36) | 276.07 (226.3, 343.13) | 497.17 (400.86, 609.31) | 949.02 (771.41, 1157.66) | 1541.88 (1265.49, 1860.62) | 2085.63 (1730.3, 2540.7) | 2610.17 (2168.23, 3171.17) | 2729.23 (2270.25, 3361) | 2550.55 (2092.1, 3178.26) | 2173.31 (1793.56, 2670.47) | 1829.05 (1490.32, 2277.69) | 1248.14 (1020.8, 1567) | 995.49 (803.93, 1230.57) | 858.15 (674.88, 1080.86) | 553.65 (415.04, 729.4) | 441.43 (317.48, 599.32) |
| 2001 | 45.98 (37.43, 57.89) | 124.37 (100.89, 158.35) | 260.99 (220.57, 315.14) | 440.01 (365.16, 534.31) | 821.37 (678.73, 979.14) | 1302.88 (1093.01, 1556.07) | 1767.24 (1471.66, 2094.65) | 2111.03 (1785.57, 2506.07) | 2337.72 (2005.25, 2802.93) | 2186.66 (1850.08, 2602.41) | 1979.12 (1678.54, 2398.91) | 1719.73 (1452.41, 2056.1) | 1212.73 (1010.84, 1448.21) | 953.28 (786.73, 1183.38) | 783.46 (625.53, 1012.14) | 490.36 (383.14, 653.44) | 411.92 (298.86, 587.72) |
| 2011 | 37.33 (29.94, 45.9) | 105.75 (85.76, 132.79) | 212.73 (176.33, 259.75) | 364.75 (293.73, 445.36) | 629.1 (518.06, 777.28) | 1012.96 (846.94, 1229.72) | 1353.38 (1139.52, 1642.75) | 1735.12 (1465.17, 2092.31) | 1849.38 (1541.43, 2187.76) | 1780.39 (1498.55, 2112.65) | 1649.46 (1406.58, 1914.17) | 1540.1 (1299.93, 1806.08) | 1087.78 (892.24, 1325.11) | 931.89 (771.86, 1156.5) | 777.2 (627.26, 961.59) | 515.39 (389.09, 676.37) | 426.04 (309.52, 575.56) |
| 2021 | 32.7 (25.38, 41.43) | 93.11 (74.02, 115.48) | 189.74 (153.21, 227.39) | 337.11 (269.14, 421.58) | 579.94 (465.17, 721.69) | 923.69 (751.54, 1122.54) | 1220.83 (1005.64, 1505.35) | 1510.49 (1251.49, 1835.98) | 1760.57 (1433.32, 2107.56) | 1673.29 (1385.54, 1998.07) | 1507.86 (1274.85, 1781.3) | 1404.79 (1186.1, 1658.28) | 1024.46 (850.98, 1224.58) | 918.92 (761.5, 1105.08) | 810.24 (640.31, 979.02) | 594.3 (457.36, 731.69) | 505.83 (349.9, 661.16) |

**Table S6: Estimated Direct Economic Burden of Cervical Cancer (Discounted to 2021, in million USD)**

| Location | Annual Growth Rate in Health Expenditure (CAGR) | 1990 Economic Burden (95% UI) | 2021 Economic Burden (95% UI) | 1990–2021 Cumulative Burden (95% UI) |
| --- | --- | --- | --- | --- |
| Afghanistan | 0.0819 | 0.01 (0.01, 0.02) | 0.64 (0.33, 0.96) | 4.89 (2.60, 7.45) |
| Albania | 0.0875 | 0.00 (0.00, 0.00) | 0.10 (0.07, 0.15) | 1.00 (0.67, 1.41) |
| Algeria | 0.0497 | 0.08 (0.06, 0.10) | 1.59 (1.23, 2.06) | 15.70 (12.40, 19.68) |
| Andorra | 0.0461 | 0.00 (0.00, 0.00) | 0.00 (0.00, 0.01) | 0.05 (0.04, 0.08) |
| Angola | 0.0972 | 0.03 (0.02, 0.04) | 4.35 (2.84, 6.42) | 28.16 (19.49, 40.24) |
| Antigua and Barbuda | 0.0340 | 0.00 (0.00, 0.00) | 0.01 (0.01, 0.02) | 0.19 (0.17, 0.21) |
| Argentina | 0.0309 | 0.62 (0.57, 0.66) | 7.07 (6.44, 7.81) | 92.84 (85.10, 100.97) |
| Armenia | 0.1602 | 0.00 (0.00, 0.00) | 0.28 (0.25, 0.32) | 2.04 (1.87, 2.22) |
| Australia | 0.0667 | 0.28 (0.26, 0.31) | 4.78 (4.27, 5.29) | 50.55 (45.85, 55.44) |
| Austria | 0.0447 | 0.17 (0.16, 0.18) | 0.98 (0.87, 1.08) | 15.05 (13.75, 16.28) |
| Azerbaijan | 0.1353 | 0.00 (0.00, 0.01) | 0.83 (0.63, 1.05) | 5.69 (4.48, 7.07) |
| Bahamas | 0.0419 | 0.01 (0.00, 0.01) | 0.08 (0.07, 0.11) | 0.95 (0.81, 1.11) |
| Bahrain | 0.0418 | 0.00 (0.00, 0.00) | 0.02 (0.02, 0.03) | 0.25 (0.20, 0.32) |
| Bangladesh | 0.0951 | 0.08 (0.06, 0.11) | 5.77 (3.94, 8.35) | 41.08 (28.85, 58.25) |
| Barbados | 0.0341 | 0.01 (0.01, 0.01) | 0.09 (0.07, 0.12) | 1.24 (1.08, 1.42) |
| Belarus | 0.1066 | 0.02 (0.02, 0.02) | 1.03 (0.82, 1.29) | 9.31 (8.34, 10.39) |
| Belgium | 0.0499 | 0.15 (0.13, 0.16) | 1.39 (1.25, 1.55) | 18.20 (16.55, 19.96) |
| Belize | 0.0352 | 0.00 (0.00, 0.00) | 0.04 (0.04, 0.05) | 0.43 (0.39, 0.47) |
| Benin | 0.0396 | 0.03 (0.02, 0.04) | 0.64 (0.45, 0.84) | 6.36 (4.71, 8.25) |
| Bhutan | 0.0764 | 0.00 (0.00, 0.00) | 0.04 (0.03, 0.06) | 0.34 (0.23, 0.48) |
| Bolivia (Plurinational State of) | 0.0936 | 0.02 (0.02, 0.03) | 1.81 (1.24, 2.57) | 13.71 (9.69, 18.69) |
| Bosnia and Herzegovina | 0.0870 | 0.01 (0.01, 0.01) | 0.26 (0.19, 0.34) | 2.82 (2.26, 3.40) |
| Botswana | 0.0388 | 0.02 (0.01, 0.02) | 0.28 (0.19, 0.46) | 3.17 (2.13, 5.09) |
| Brazil | 0.0462 | 1.48 (1.43, 1.54) | 31.14 (29.37, 32.72) | 312.49 (299.81, 324.06) |
| Brunei Darussalam | 0.0114 | 0.01 (0.01, 0.01) | 0.05 (0.04, 0.07) | 0.72 (0.58, 0.89) |
| Bulgaria | 0.1137 | 0.02 (0.02, 0.03) | 1.61 (1.31, 1.90) | 13.42 (11.80, 15.19) |
| Burkina Faso | 0.0974 | 0.02 (0.01, 0.02) | 1.41 (1.03, 1.85) | 10.30 (7.56, 13.43) |
| Burundi | 0.0691 | 0.02 (0.01, 0.03) | 0.59 (0.41, 0.83) | 5.12 (3.70, 7.08) |
| Cabo Verde | 0.0739 | 0.00 (0.00, 0.00) | 0.05 (0.04, 0.07) | 0.48 (0.39, 0.61) |
| Cambodia | 0.0822 | 0.02 (0.01, 0.02) | 0.87 (0.63, 1.21) | 6.91 (5.16, 9.19) |
| Cameroon | 0.0399 | 0.10 (0.08, 0.12) | 2.38 (1.50, 3.38) | 24.11 (15.98, 33.04) |
| Canada | 0.0500 | 0.61 (0.56, 0.66) | 10.73 (9.68, 11.70) | 117.48 (106.45, 128.47) |
| Central African Republic | 0.0742 | 0.01 (0.01, 0.02) | 0.51 (0.33, 0.74) | 4.47 (2.87, 6.49) |
| Chad | 0.0649 | 0.02 (0.02, 0.03) | 1.04 (0.73, 1.39) | 8.46 (6.19, 11.21) |
| Chile | 0.0711 | 0.14 (0.13, 0.16) | 3.43 (3.08, 3.80) | 32.88 (30.04, 35.93) |
| China | 0.1334 | 0.92 (0.74, 1.14) | 256.14 (185.10, 332.94) | 1601.98 (1245.77, 1977.51) |
| Colombia | 0.0617 | 0.20 (0.18, 0.22) | 6.94 (5.68, 8.40) | 63.30 (57.19, 69.87) |
| Comoros | 0.0441 | 0.00 (0.00, 0.01) | 0.08 (0.06, 0.12) | 0.86 (0.59, 1.20) |
| Congo | 0.0499 | 0.03 (0.02, 0.04) | 0.84 (0.47, 1.26) | 8.12 (4.83, 11.52) |
| Cook Islands | 0.0841 | 0.00 (0.00, 0.00) | 0.00 (0.00, 0.00) | 0.01 (0.01, 0.01) |
| Costa Rica | 0.0639 | 0.02 (0.02, 0.03) | 0.75 (0.65, 0.87) | 6.59 (5.93, 7.27) |
| Croatia | 0.0603 | 0.05 (0.04, 0.05) | 0.52 (0.40, 0.65) | 6.57 (5.41, 7.81) |
| Cuba | 0.0992 | 0.06 (0.05, 0.06) | 3.53 (2.93, 4.16) | 29.63 (26.73, 32.70) |
| Cyprus | 0.0628 | 0.00 (0.00, 0.00) | 0.09 (0.07, 0.11) | 0.91 (0.74, 1.09) |
| Czechia | 0.0922 | 0.04 (0.04, 0.05) | 1.07 (0.91, 1.27) | 11.17 (9.87, 12.64) |
| Cote d'Ivoire | 0.0438 | 0.04 (0.03, 0.05) | 1.04 (0.68, 1.47) | 10.53 (7.10, 14.48) |
| Democratic People's Republic of Korea | 0.0399 | 0.16 (0.11, 0.22) | 2.65 (1.81, 3.75) | 30.42 (21.08, 42.08) |
| Democratic Republic of the Congo | 0.0151 | 0.37 (0.26, 0.50) | 3.60 (2.37, 5.09) | 44.49 (30.97, 60.75) |
| Denmark | 0.0420 | 0.19 (0.17, 0.20) | 0.83 (0.75, 0.91) | 13.10 (11.96, 14.26) |
| Djibouti | 0.0387 | 0.00 (0.00, 0.00) | 0.09 (0.06, 0.14) | 0.88 (0.52, 1.36) |
| Dominica | 0.0369 | 0.00 (0.00, 0.00) | 0.01 (0.01, 0.01) | 0.15 (0.12, 0.19) |
| Dominican Republic | 0.0564 | 0.03 (0.03, 0.04) | 1.01 (0.73, 1.33) | 10.15 (7.70, 12.99) |
| Ecuador | 0.1115 | 0.02 (0.02, 0.02) | 3.06 (2.34, 3.89) | 21.41 (18.24, 24.88) |
| Egypt | 0.0419 | 0.04 (0.03, 0.05) | 1.23 (0.89, 1.62) | 12.37 (10.00, 15.10) |
| El Salvador | 0.0546 | 0.03 (0.03, 0.04) | 0.98 (0.74, 1.25) | 9.78 (8.46, 11.14) |
| Equatorial Guinea | 0.1124 | 0.00 (0.00, 0.00) | 0.23 (0.12, 0.37) | 1.35 (0.74, 2.19) |
| Eritrea | 0.0303 | 0.05 (0.03, 0.06) | 0.56 (0.35, 0.83) | 6.30 (3.98, 9.09) |
| Estonia | 0.1079 | 0.01 (0.01, 0.01) | 0.32 (0.27, 0.37) | 2.83 (2.53, 3.17) |
| Eswatini | 0.0611 | 0.01 (0.00, 0.01) | 0.24 (0.13, 0.39) | 2.56 (1.46, 3.96) |
| Ethiopia | 0.0770 | 0.15 (0.11, 0.22) | 4.08 (2.98, 6.08) | 34.21 (25.89, 49.77) |
| Fiji | 0.0733 | 0.01 (0.01, 0.01) | 0.26 (0.18, 0.35) | 2.41 (1.82, 3.03) |
| Finland | 0.0486 | 0.05 (0.04, 0.05) | 0.46 (0.40, 0.51) | 5.86 (5.23, 6.50) |
| France | 0.0376 | 0.90 (0.84, 0.97) | 6.54 (5.89, 7.18) | 95.72 (87.18, 104.02) |
| Gabon | 0.0322 | 0.02 (0.01, 0.02) | 0.21 (0.13, 0.32) | 2.51 (1.69, 3.57) |
| Gambia | -0.0423 | 0.03 (0.02, 0.04) | 0.07 (0.04, 0.09) | 1.45 (0.99, 1.93) |
| Georgia | 0.1058 | 0.01 (0.01, 0.01) | 0.29 (0.25, 0.34) | 2.55 (2.26, 2.86) |
| Germany | 0.0434 | 1.54 (1.45, 1.65) | 10.10 (9.04, 11.02) | 136.20 (125.14, 147.01) |
| Ghana | 0.0984 | 0.03 (0.03, 0.05) | 4.13 (2.82, 5.44) | 28.92 (20.37, 37.66) |
| Greece | 0.0319 | 0.16 (0.15, 0.17) | 0.89 (0.81, 0.96) | 13.44 (12.43, 14.36) |
| Grenada | 0.0322 | 0.00 (0.00, 0.00) | 0.02 (0.02, 0.02) | 0.26 (0.23, 0.29) |
| Guatemala | 0.0733 | 0.03 (0.03, 0.03) | 2.34 (2.00, 2.75) | 21.24 (19.71, 22.91) |
| Guinea | 0.0410 | 0.04 (0.03, 0.06) | 0.71 (0.49, 0.97) | 7.79 (5.70, 10.23) |
| Guinea-Bissau | 0.0756 | 0.00 (0.00, 0.00) | 0.19 (0.13, 0.25) | 1.51 (1.07, 1.96) |
| Guyana | 0.1023 | 0.00 (0.00, 0.00) | 0.10 (0.07, 0.13) | 0.81 (0.64, 1.02) |
| Haiti | 0.0250 | 0.10 (0.07, 0.13) | 0.91 (0.62, 1.28) | 11.72 (8.15, 15.97) |
| Honduras | 0.0608 | 0.02 (0.01, 0.02) | 0.87 (0.52, 1.28) | 7.36 (4.63, 10.62) |
| Hungary | 0.0675 | 0.12 (0.10, 0.14) | 1.79 (1.46, 2.20) | 21.75 (18.45, 25.62) |
| Iceland | 0.0410 | 0.00 (0.00, 0.00) | 0.03 (0.02, 0.03) | 0.32 (0.28, 0.36) |
| India | 0.0686 | 2.19 (1.82, 2.55) | 65.92 (56.31, 75.89) | 568.10 (499.18, 639.17) |
| Indonesia | 0.1009 | 0.24 (0.19, 0.31) | 23.69 (17.23, 31.46) | 175.18 (130.13, 226.01) |
| Iran (Islamic Republic of) | 0.0508 | 0.03 (0.03, 0.04) | 0.74 (0.64, 0.84) | 7.62 (6.77, 8.50) |
| Iraq | 0.1254 | 0.00 (0.00, 0.00) | 0.93 (0.67, 1.29) | 5.86 (4.24, 8.08) |
| Ireland | 0.0665 | 0.03 (0.03, 0.03) | 0.61 (0.54, 0.68) | 7.58 (6.78, 8.42) |
| Israel | 0.0490 | 0.02 (0.02, 0.03) | 0.42 (0.37, 0.47) | 5.03 (4.52, 5.54) |
| Italy | 0.0332 | 0.47 (0.44, 0.49) | 6.99 (6.31, 7.54) | 92.14 (84.13, 98.44) |
| Jamaica | 0.0407 | 0.03 (0.03, 0.03) | 0.41 (0.30, 0.55) | 4.83 (4.07, 5.71) |
| Japan | 0.0159 | 3.70 (3.51, 3.86) | 19.13 (17.38, 20.41) | 308.41 (284.24, 326.31) |
| Jordan | 0.0308 | 0.01 (0.01, 0.01) | 0.11 (0.08, 0.15) | 1.17 (0.90, 1.51) |
| Kazakhstan | 0.1026 | 0.03 (0.03, 0.04) | 1.87 (1.58, 2.17) | 15.88 (14.23, 17.60) |
| Kenya | 0.0674 | 0.04 (0.03, 0.06) | 2.55 (1.79, 3.47) | 21.66 (15.74, 30.68) |
| Kiribati | 0.0560 | 0.00 (0.00, 0.00) | 0.02 (0.01, 0.03) | 0.18 (0.13, 0.24) |
| Kuwait | 0.0650 | 0.00 (0.00, 0.00) | 0.08 (0.07, 0.10) | 0.66 (0.58, 0.76) |
| Kyrgyzstan | 0.0934 | 0.01 (0.01, 0.01) | 0.36 (0.28, 0.45) | 2.93 (2.43, 3.51) |
| Lao People's Democratic Republic | 0.0504 | 0.02 (0.01, 0.02) | 0.24 (0.17, 0.31) | 2.55 (1.88, 3.35) |
| Latvia | 0.1053 | 0.01 (0.01, 0.01) | 0.22 (0.19, 0.25) | 1.97 (1.77, 2.17) |
| Lebanon | -0.0037 | 0.03 (0.02, 0.03) | 0.08 (0.06, 0.10) | 1.47 (1.16, 1.83) |
| Lesotho | 0.0818 | 0.01 (0.00, 0.01) | 0.47 (0.27, 0.68) | 4.23 (2.59, 6.02) |
| Liberia | 0.1185 | 0.00 (0.00, 0.00) | 0.34 (0.23, 0.47) | 2.06 (1.42, 2.79) |
| Libya | 0.0054 | 0.04 (0.03, 0.05) | 0.36 (0.25, 0.50) | 4.96 (3.82, 6.47) |
| Lithuania | 0.1011 | 0.02 (0.02, 0.02) | 0.61 (0.52, 0.70) | 5.52 (4.97, 6.12) |
| Luxembourg | 0.0422 | 0.01 (0.01, 0.01) | 0.05 (0.05, 0.06) | 0.77 (0.70, 0.84) |
| Madagascar | 0.0173 | 0.12 (0.09, 0.16) | 1.17 (0.72, 1.67) | 14.30 (9.37, 19.33) |
| Malawi | 0.0706 | 0.03 (0.02, 0.04) | 1.30 (0.85, 1.84) | 10.94 (7.80, 14.62) |
| Malaysia | 0.0662 | 0.09 (0.07, 0.10) | 4.00 (3.10, 4.72) | 31.32 (25.54, 36.33) |
| Maldives | 0.0781 | 0.00 (0.00, 0.00) | 0.01 (0.01, 0.01) | 0.10 (0.08, 0.12) |
| Mali | 0.0348 | 0.07 (0.05, 0.08) | 0.95 (0.69, 1.28) | 10.37 (7.84, 13.28) |
| Malta | 0.0776 | 0.00 (0.00, 0.00) | 0.02 (0.02, 0.03) | 0.21 (0.18, 0.23) |
| Marshall Islands | 0.0420 | 0.00 (0.00, 0.00) | 0.01 (0.00, 0.01) | 0.08 (0.05, 0.10) |
| Mauritania | 0.0642 | 0.01 (0.01, 0.01) | 0.32 (0.23, 0.44) | 2.64 (1.92, 3.49) |
| Mauritius | 0.0769 | 0.00 (0.00, 0.00) | 0.14 (0.12, 0.15) | 1.22 (1.13, 1.33) |
| Mexico | 0.0329 | 2.01 (1.96, 2.06) | 17.83 (14.83, 20.86) | 225.66 (215.94, 235.48) |
| Micronesia (Federated States of) | 0.0417 | 0.00 (0.00, 0.00) | 0.01 (0.01, 0.01) | 0.11 (0.08, 0.15) |
| Monaco | 0.0440 | 0.00 (0.00, 0.00) | 0.01 (0.01, 0.01) | 0.10 (0.07, 0.13) |
| Mongolia | 0.1341 | 0.00 (0.00, 0.00) | 0.41 (0.31, 0.54) | 2.69 (1.97, 3.59) |
| Montenegro | 0.0619 | 0.00 (0.00, 0.00) | 0.06 (0.05, 0.08) | 0.78 (0.63, 0.95) |
| Morocco | 0.0606 | 0.06 (0.04, 0.08) | 2.04 (1.37, 2.80) | 19.07 (13.35, 25.76) |
| Mozambique | 0.0929 | 0.04 (0.03, 0.06) | 3.78 (2.30, 6.18) | 27.49 (17.37, 44.80) |
| Myanmar | 0.1235 | 0.05 (0.04, 0.07) | 5.47 (3.89, 7.52) | 38.38 (27.95, 52.25) |
| Namibia | 0.0326 | 0.01 (0.01, 0.02) | 0.24 (0.15, 0.35) | 2.66 (1.77, 3.73) |
| Nauru | 0.0747 | 0.00 (0.00, 0.00) | 0.00 (0.00, 0.00) | 0.02 (0.01, 0.03) |
| Nepal | 0.1132 | 0.01 (0.01, 0.02) | 1.30 (0.87, 1.73) | 8.34 (5.89, 11.11) |
| Netherlands | 0.0489 | 0.19 (0.17, 0.20) | 1.90 (1.70, 2.13) | 24.61 (22.24, 27.00) |
| New Zealand | 0.0714 | 0.02 (0.02, 0.02) | 0.24 (0.22, 0.27) | 2.54 (2.31, 2.78) |
| Nicaragua | 0.0640 | 0.02 (0.02, 0.02) | 0.66 (0.51, 0.85) | 6.47 (5.42, 7.71) |
| Niger | 0.0498 | 0.02 (0.02, 0.03) | 0.72 (0.50, 1.04) | 6.56 (4.74, 9.16) |
| Nigeria | 0.0787 | 0.20 (0.14, 0.28) | 13.16 (8.18, 19.56) | 100.99 (63.75, 149.06) |
| Niue | 0.0975 | 0.00 (0.00, 0.00) | 0.00 (0.00, 0.00) | 0.00 (0.00, 0.00) |
| North Macedonia | 0.0579 | 0.01 (0.01, 0.01) | 0.19 (0.14, 0.25) | 2.33 (1.92, 2.78) |
| Norway | 0.0504 | 0.08 (0.07, 0.08) | 0.82 (0.76, 0.89) | 9.66 (8.99, 10.29) |
| Oman | 0.0462 | 0.00 (0.00, 0.01) | 0.06 (0.04, 0.08) | 0.62 (0.48, 0.79) |
| Pakistan | 0.0430 | 0.12 (0.09, 0.14) | 3.14 (2.20, 4.34) | 31.31 (23.35, 40.97) |
| Palau | 0.0501 | 0.00 (0.00, 0.00) | 0.01 (0.01, 0.01) | 0.08 (0.07, 0.11) |
| Panama | 0.0749 | 0.02 (0.02, 0.02) | 0.79 (0.63, 0.96) | 6.68 (5.96, 7.48) |
| Papua New Guinea | 0.0686 | 0.01 (0.01, 0.02) | 0.68 (0.49, 1.16) | 5.54 (4.03, 9.08) |
| Paraguay | 0.0718 | 0.01 (0.01, 0.02) | 0.81 (0.59, 1.11) | 7.02 (5.52, 8.81) |
| Peru | 0.0790 | 0.10 (0.08, 0.12) | 6.88 (4.90, 9.12) | 52.35 (40.66, 65.64) |
| Philippines | 0.0814 | 0.08 (0.06, 0.09) | 5.52 (4.15, 7.09) | 42.77 (37.01, 48.84) |
| Poland | 0.0847 | 0.20 (0.19, 0.20) | 4.48 (3.96, 4.99) | 48.13 (45.49, 50.68) |
| Portugal | 0.0451 | 0.11 (0.10, 0.12) | 1.06 (0.95, 1.17) | 14.50 (13.15, 15.88) |
| Qatar | 0.0546 | 0.00 (0.00, 0.00) | 0.04 (0.03, 0.05) | 0.30 (0.23, 0.40) |
| Republic of Korea | 0.0875 | 0.14 (0.12, 0.17) | 5.15 (4.05, 6.72) | 46.95 (38.68, 57.02) |
| Republic of Moldova | 0.1422 | 0.00 (0.00, 0.00) | 0.42 (0.37, 0.48) | 2.84 (2.60, 3.10) |
| Romania | 0.1233 | 0.07 (0.07, 0.08) | 6.65 (5.71, 7.60) | 52.55 (47.70, 57.71) |
| Russian Federation | 0.1166 | 0.29 (0.28, 0.30) | 35.11 (31.06, 38.94) | 247.65 (237.25, 257.78) |
| Rwanda | 0.1024 | 0.03 (0.02, 0.03) | 1.59 (1.02, 2.32) | 11.14 (7.70, 15.52) |
| Saint Kitts and Nevis | 0.0443 | 0.00 (0.00, 0.00) | 0.01 (0.01, 0.02) | 0.15 (0.13, 0.17) |
| Saint Lucia | 0.0377 | 0.00 (0.00, 0.00) | 0.03 (0.02, 0.03) | 0.30 (0.26, 0.34) |
| Saint Vincent and the Grenadines | 0.0496 | 0.00 (0.00, 0.00) | 0.02 (0.02, 0.03) | 0.25 (0.22, 0.28) |
| Samoa | 0.0639 | 0.00 (0.00, 0.00) | 0.03 (0.02, 0.03) | 0.23 (0.16, 0.31) |
| San Marino | 0.0266 | 0.00 (0.00, 0.00) | 0.00 (0.00, 0.00) | 0.02 (0.01, 0.03) |
| Sao Tome and Principe | 0.0604 | 0.00 (0.00, 0.00) | 0.03 (0.02, 0.04) | 0.26 (0.19, 0.34) |
| Saudi Arabia | 0.0547 | 0.01 (0.01, 0.02) | 0.68 (0.48, 0.95) | 5.97 (4.53, 7.98) |
| Senegal | 0.0508 | 0.04 (0.03, 0.05) | 1.12 (0.80, 1.50) | 10.67 (8.01, 13.87) |
| Serbia | 0.1343 | 0.01 (0.01, 0.02) | 1.74 (1.36, 2.21) | 13.11 (10.75, 15.64) |
| Seychelles | 0.0330 | 0.00 (0.00, 0.00) | 0.02 (0.01, 0.02) | 0.21 (0.18, 0.25) |
| Sierra Leone | 0.0367 | 0.01 (0.01, 0.02) | 0.33 (0.24, 0.45) | 3.35 (2.42, 4.41) |
| Singapore | 0.0799 | 0.02 (0.02, 0.03) | 0.83 (0.75, 0.91) | 7.48 (6.76, 8.24) |
| Slovakia | 0.0996 | 0.02 (0.02, 0.02) | 0.85 (0.63, 1.09) | 7.39 (5.93, 8.90) |
| Slovenia | 0.0599 | 0.03 (0.02, 0.03) | 0.33 (0.26, 0.39) | 4.44 (3.90, 5.01) |
| Solomon Islands | 0.0343 | 0.00 (0.00, 0.00) | 0.04 (0.03, 0.06) | 0.39 (0.27, 0.56) |
| Somalia | 0.0199 | 0.24 (0.16, 0.33) | 2.45 (1.65, 3.67) | 30.56 (20.72, 44.42) |
| South Africa | 0.0409 | 0.46 (0.39, 0.58) | 11.69 (10.20, 13.32) | 120.83 (109.41, 133.02) |
| South Sudan | 0.1198 | 0.01 (0.01, 0.01) | 1.46 (0.98, 2.14) | 8.62 (5.58, 12.81) |
| Spain | 0.0495 | 0.40 (0.37, 0.44) | 4.46 (3.98, 4.97) | 56.84 (51.32, 62.35) |
| Sri Lanka | 0.0575 | 0.03 (0.02, 0.03) | 0.69 (0.43, 0.97) | 7.26 (5.67, 9.02) |
| Sudan | 0.0377 | 0.03 (0.02, 0.05) | 0.49 (0.28, 0.74) | 5.27 (3.24, 7.98) |
| Suriname | 0.0334 | 0.01 (0.01, 0.01) | 0.10 (0.07, 0.12) | 1.17 (0.94, 1.42) |
| Sweden | 0.0463 | 0.13 (0.12, 0.14) | 1.19 (1.01, 1.36) | 16.70 (15.33, 18.12) |
| Switzerland | 0.0525 | 0.12 (0.11, 0.13) | 0.90 (0.79, 1.00) | 12.88 (11.64, 14.10) |
| Syrian Arab Republic | -0.0199 | 0.04 (0.03, 0.05) | 0.09 (0.06, 0.13) | 2.03 (1.52, 2.62) |
| Tajikistan | 0.1249 | 0.00 (0.00, 0.00) | 0.19 (0.13, 0.29) | 1.24 (0.91, 1.69) |
| Thailand | 0.0865 | 0.25 (0.20, 0.30) | 13.63 (10.35, 17.68) | 116.56 (93.61, 143.56) |
| Timor-Leste | 0.1373 | 0.00 (0.00, 0.00) | 0.05 (0.03, 0.06) | 0.27 (0.20, 0.36) |
| Togo | 0.0767 | 0.01 (0.01, 0.01) | 0.63 (0.41, 0.86) | 4.87 (3.37, 6.44) |
| Tonga | 0.0931 | 0.00 (0.00, 0.00) | 0.02 (0.02, 0.03) | 0.18 (0.14, 0.25) |
| Trinidad and Tobago | 0.0745 | 0.01 (0.01, 0.01) | 0.41 (0.31, 0.54) | 3.73 (3.24, 4.28) |
| Tunisia | 0.0405 | 0.02 (0.01, 0.02) | 0.25 (0.17, 0.34) | 2.89 (2.04, 3.91) |
| Turkey | 0.0320 | 0.20 (0.15, 0.26) | 1.83 (1.40, 2.35) | 23.24 (18.69, 28.59) |
| Turkmenistan | 0.0970 | 0.00 (0.00, 0.01) | 0.42 (0.31, 0.56) | 3.29 (2.76, 3.89) |
| Tuvalu | 0.0525 | 0.00 (0.00, 0.00) | 0.00 (0.00, 0.00) | 0.02 (0.02, 0.03) |
| Uganda | 0.0460 | 0.09 (0.06, 0.12) | 2.34 (1.59, 3.14) | 22.45 (16.21, 29.36) |
| Ukraine | 0.1258 | 0.07 (0.07, 0.08) | 2.70 (1.70, 3.97) | 24.70 (20.95, 29.04) |
| United Arab Emirates | 0.0555 | 0.01 (0.00, 0.01) | 0.28 (0.21, 0.37) | 2.46 (1.83, 3.25) |
| United Kingdom | 0.0432 | 1.91 (1.86, 1.96) | 7.47 (7.12, 7.80) | 112.68 (108.57, 116.65) |
| United Republic of Tanzania | 0.0494 | 0.16 (0.12, 0.21) | 3.53 (2.39, 4.81) | 34.49 (25.03, 46.31) |
| United States of America | 0.0468 | 7.11 (6.92, 7.28) | 65.19 (62.21, 67.90) | 828.01 (798.47, 850.43) |
| Uruguay | 0.0452 | 0.06 (0.05, 0.06) | 0.70 (0.63, 0.78) | 8.29 (7.55, 9.03) |
| Uzbekistan | 0.0733 | 0.03 (0.02, 0.03) | 1.40 (1.13, 1.68) | 11.11 (9.76, 12.55) |
| Vanuatu | 0.0480 | 0.00 (0.00, 0.00) | 0.02 (0.01, 0.03) | 0.18 (0.13, 0.25) |
| Venezuela (Bolivarian Republic of) | 0.0608 | 0.24 (0.23, 0.26) | 10.15 (7.28, 13.38) | 89.24 (76.50, 103.50) |
| Viet Nam | 0.1048 | 0.05 (0.04, 0.07) | 6.85 (5.15, 9.10) | 47.23 (35.98, 61.32) |
| Yemen | 0.0234 | 0.02 (0.01, 0.04) | 0.27 (0.17, 0.40) | 3.03 (1.96, 4.74) |
| Zambia | 0.0766 | 0.05 (0.03, 0.06) | 2.54 (1.55, 4.77) | 19.55 (12.90, 35.78) |
| Zimbabwe | -0.0239 | 0.42 (0.31, 0.54) | 1.50 (1.00, 2.12) | 31.26 (22.20, 41.56) |
| Global | 0.0641 | 32.97 (29.94, 36.30) | 813.03 (645.14, 1002.04) | 7210.55 (6142.68, 8395.87) |

**Fig.S1** The factors affecting the average annual percentage changes (AAPCs) in ASIR, ASMR, ASDR of cervical cancer from 1991 to 2021 at the national level. (a) ASIR in 1991 and AAPC in ASIR. (b) ASMR in 1991 and AAPC in ASMR. (c) ASDR in 1991 and AAPC in ASDR. (d) SDI in 2021 and AAPC in ASIR. (e) SDI in 2021 and AAPC in ASMR. (f) SDI in 2021 and AAPC in ASDR. The grey line represents an adaptive association fitted with Loess regression based on all data points. The ρ indices and P-values were derived using Spearman’s rank analysis.

**(a)**

**
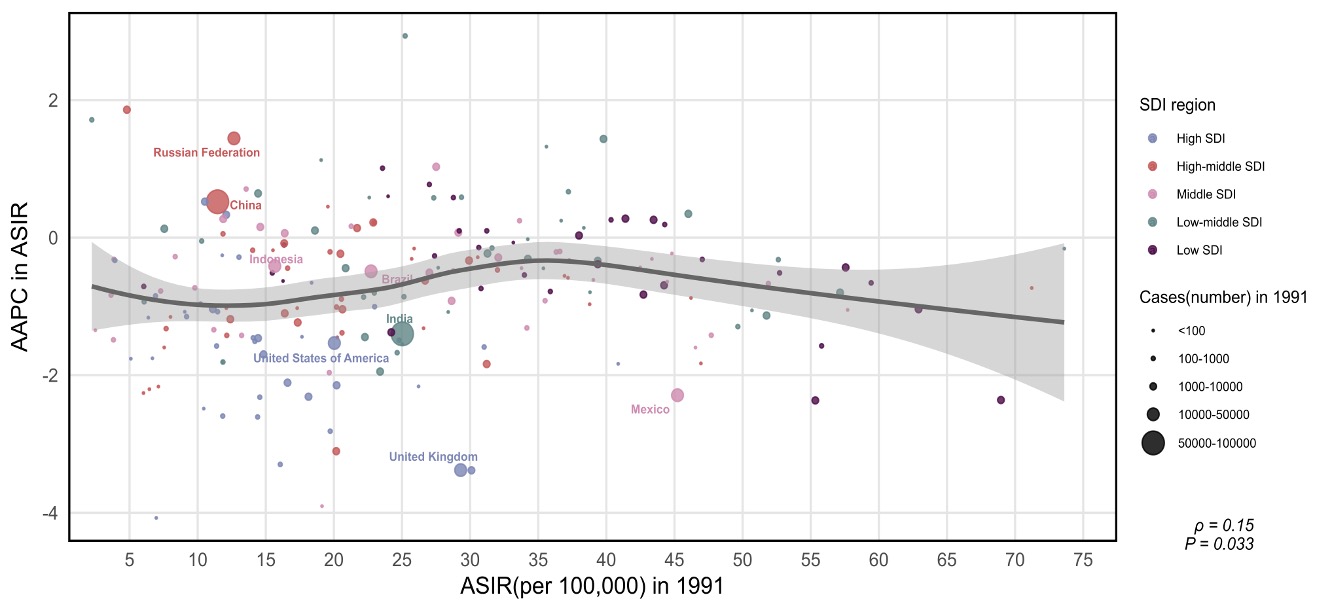
**

**（b）
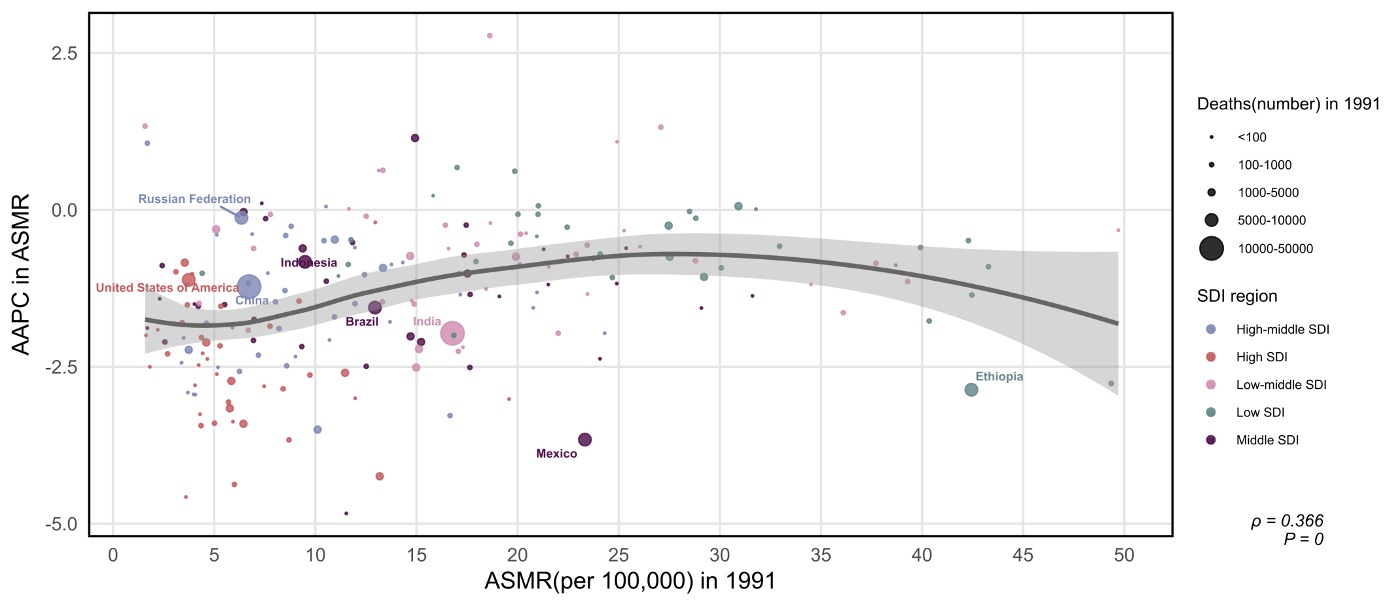
**

**（c）**

**
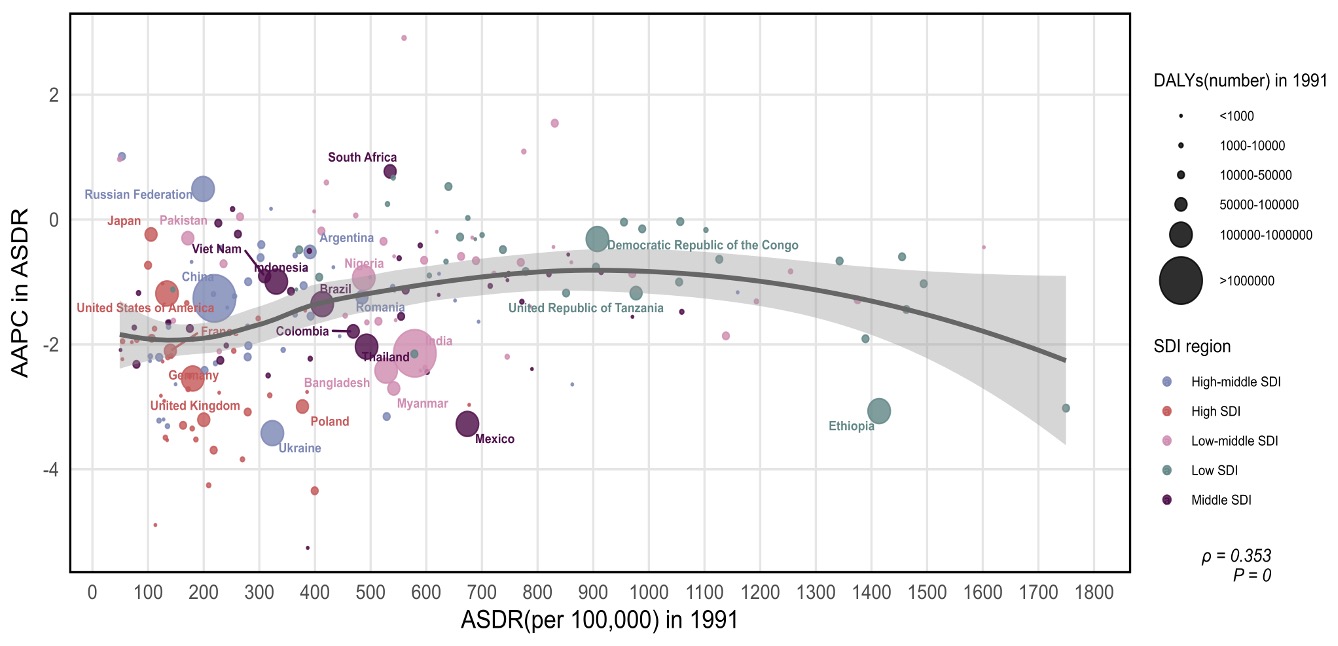
**

**（d）**

**
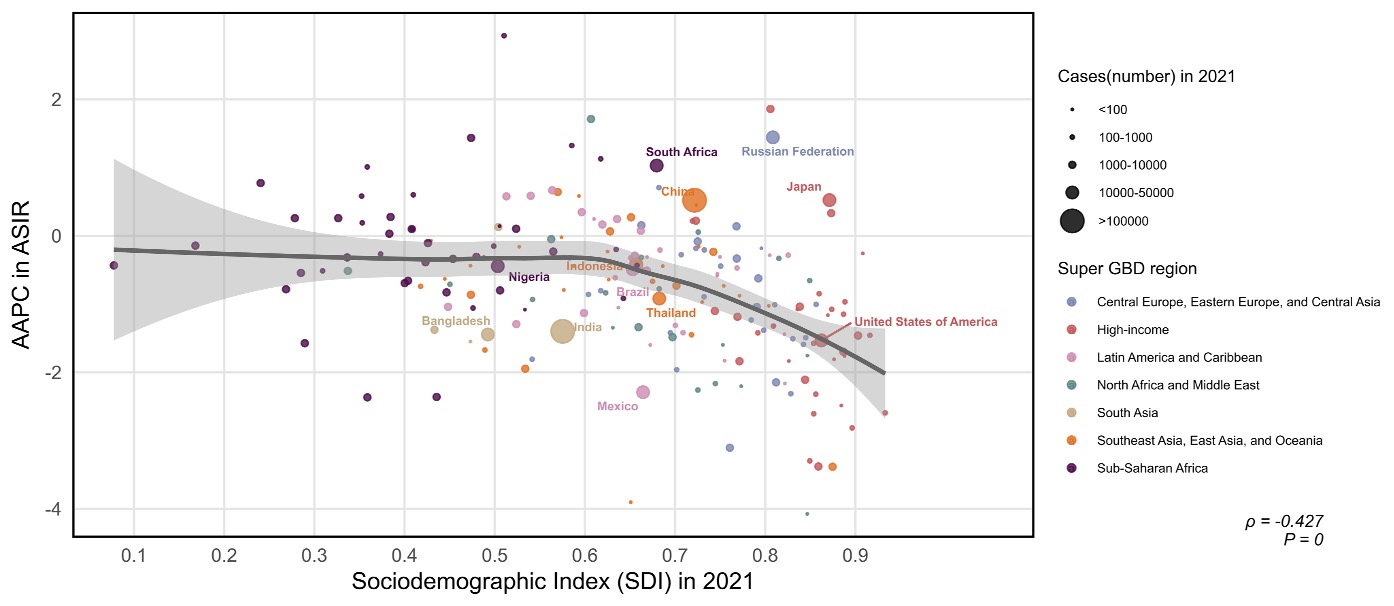
**

**（e）**

**
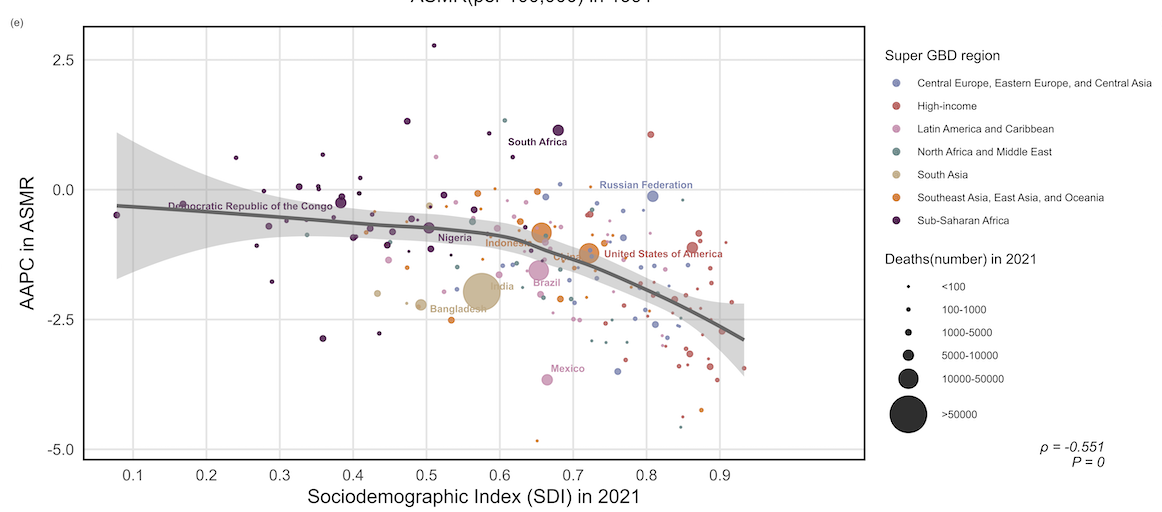
**

**（f）**

**
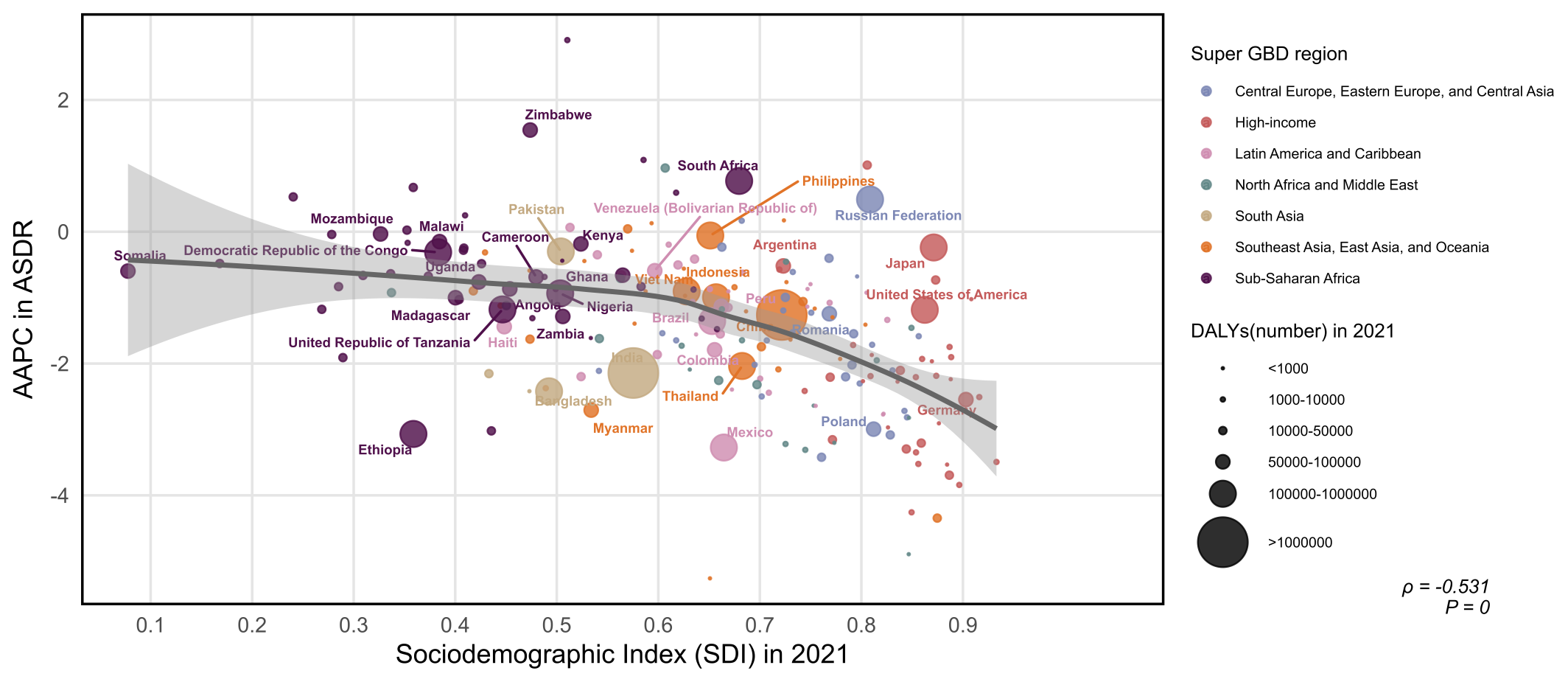
**

**Fig.S2** The correlation between AAPCs and UHC Service Coverage Index in 2021 at the national level. (a) Incidence. (b) Mortality. (c) Disability-adjusted life years (DALYs). The grey line represents an adaptive association fitted with Loess regression based on all data points. The ρ indices and P-values were derived using Spearman’s rank analysis.


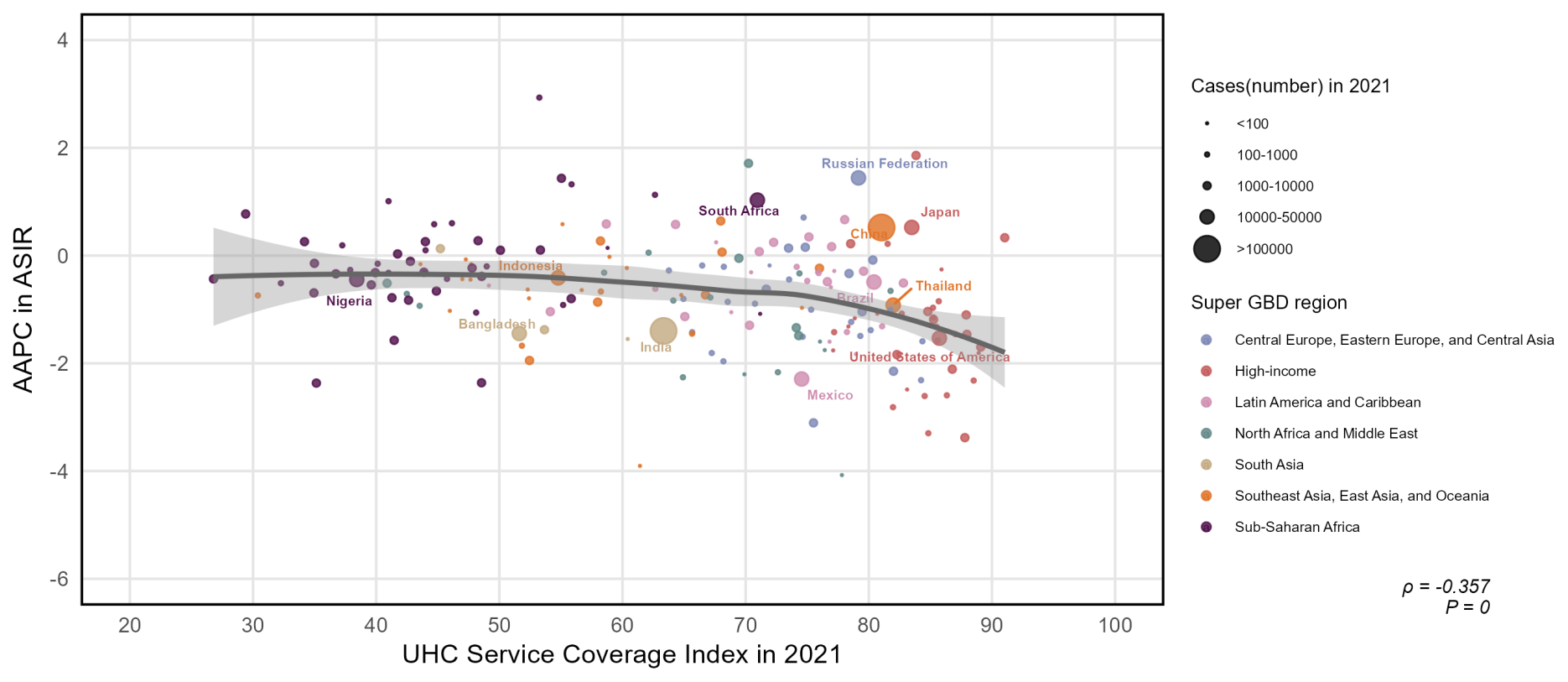


**(a)**


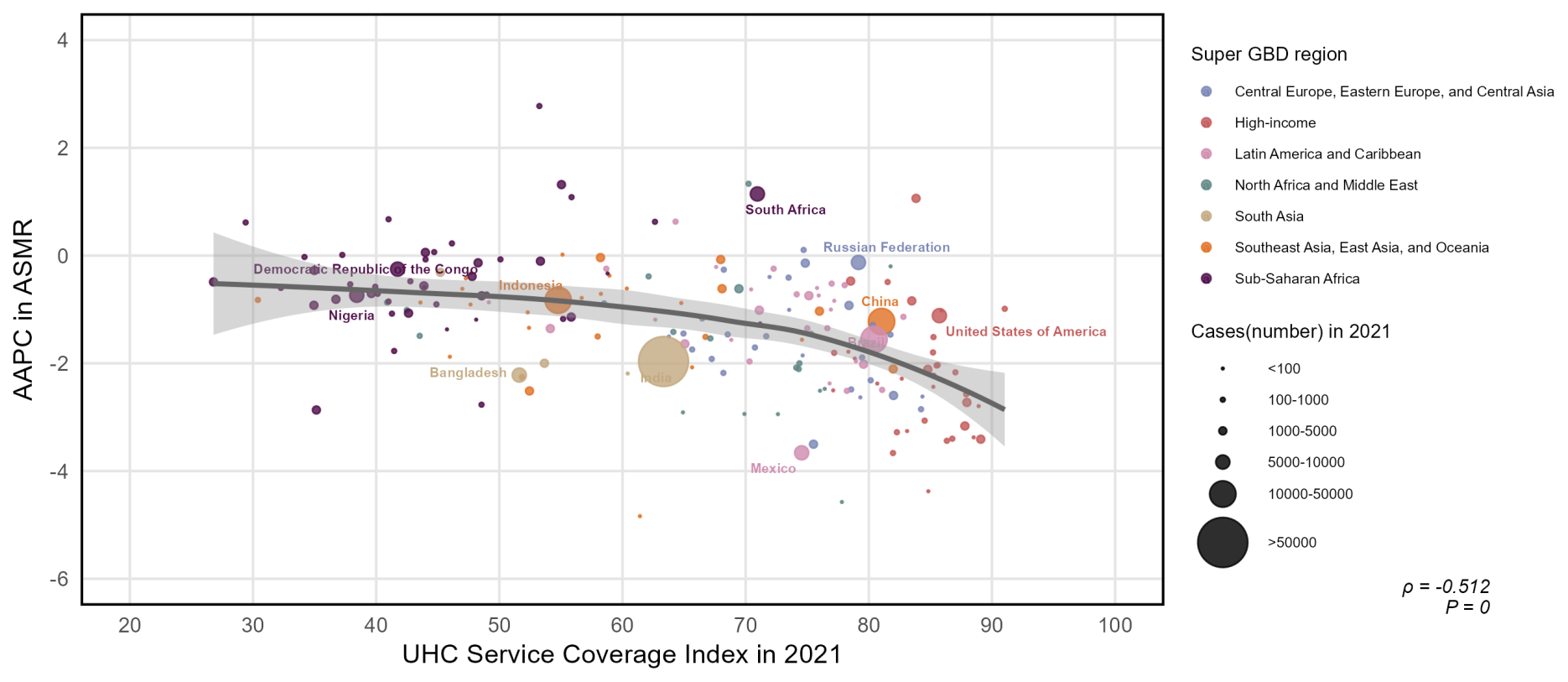


**(b)**


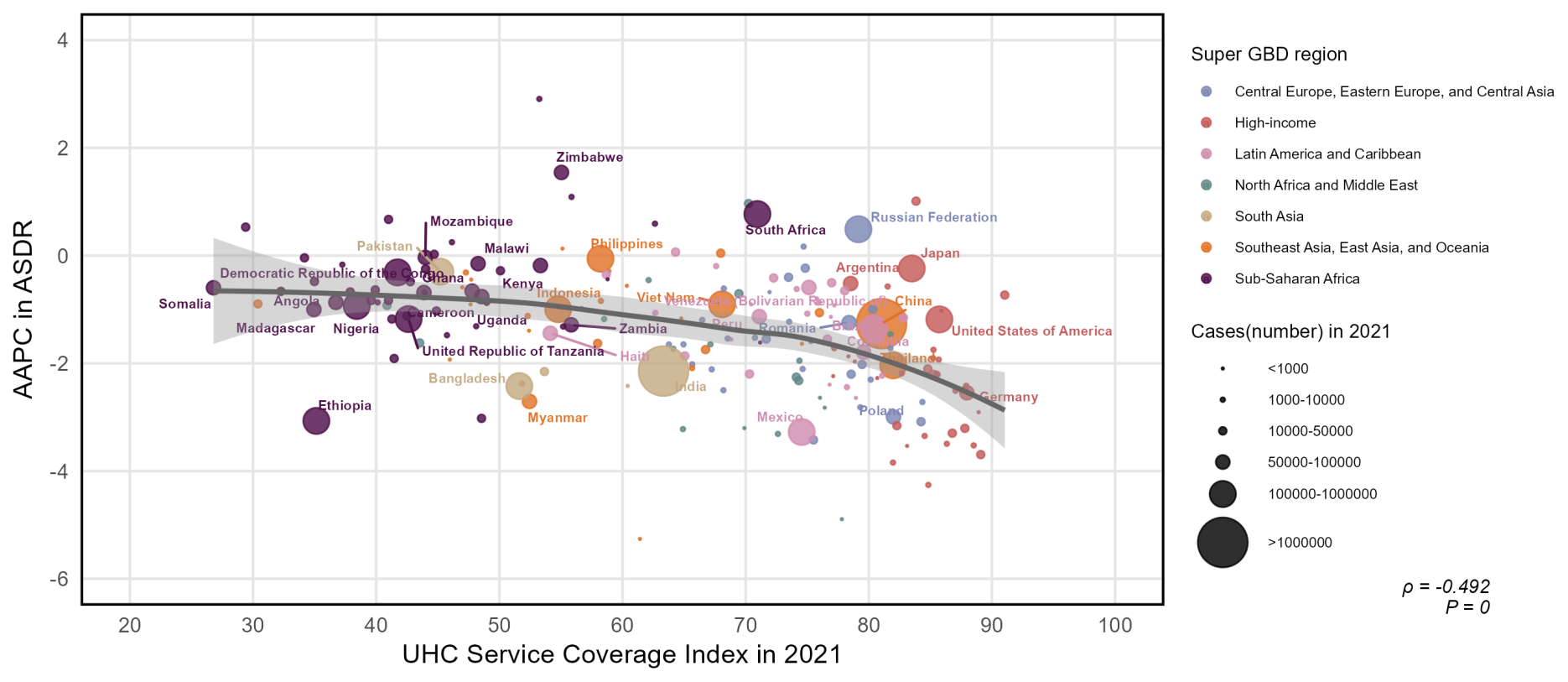


**(c)**

**Fig.S3** The trend in age-standardized rates (ASRs) of cervical cancer along with the sociodemographic index (SDI) across 21 Global Burden of Disease (GBD) regions from 2014 to 2021. The ρ indices and P-values were derived using Spearman’s rank analysis. (a) Incidence. (b) Mortality.


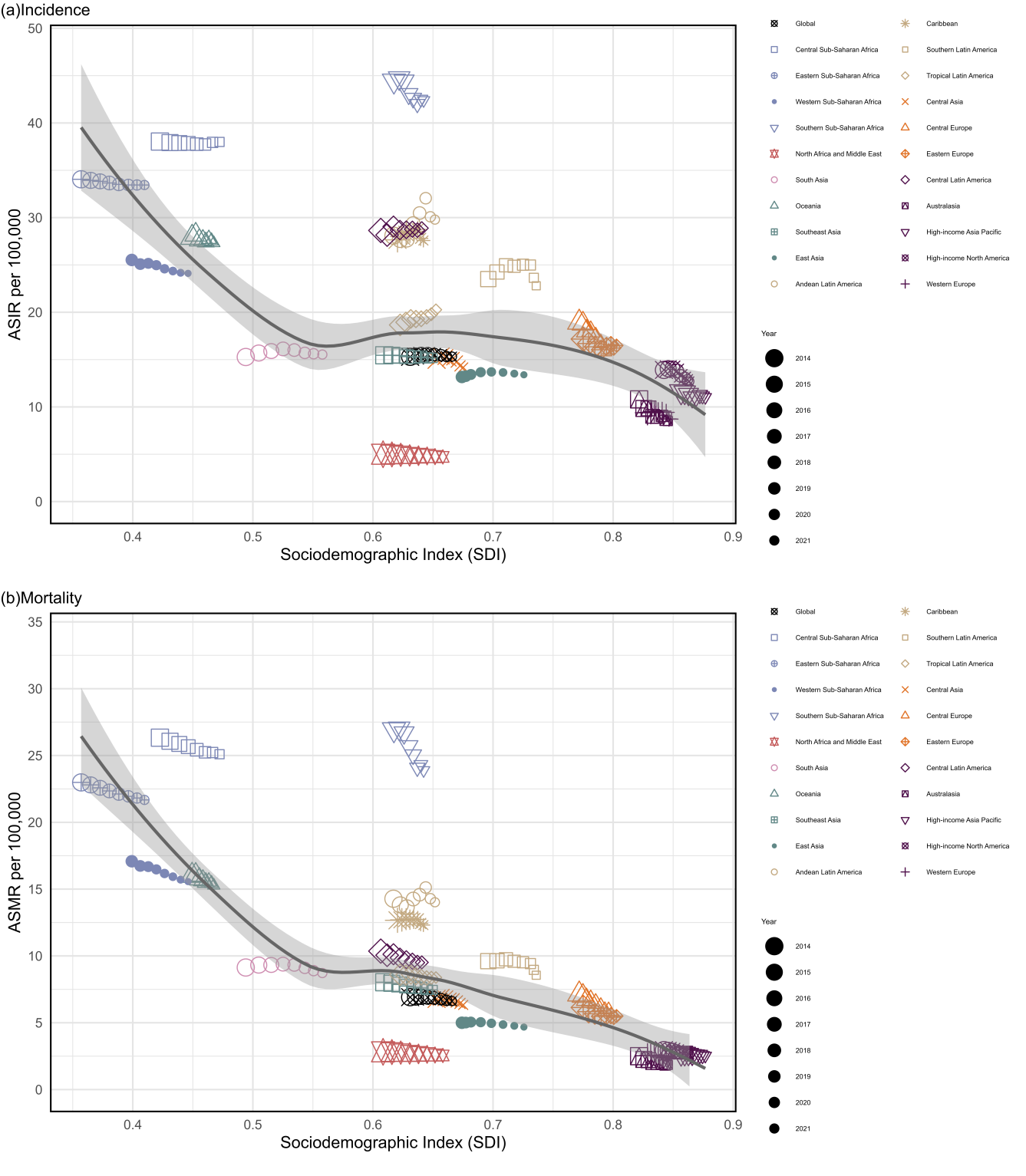

Supplement: Supplementary file 1 [file Table_1.docx]
